# Neurons Refine the *Caenorhabditis elegans* Body Plan by Directing Axial Patterning by Wnts

**DOI:** 10.1371/journal.pbio.1001465

**Published:** 2013-01-08

**Authors:** Katarzyna Modzelewska, Amara Lauritzen, Stefan Hasenoeder, Louise Brown, John Georgiou, Nadeem Moghal

**Affiliations:** 1 Department of Oncological Sciences, Huntsman Cancer Institute, University of Utah, Salt Lake City, Utah, United States of America; 2 Campbell Family Cancer Research Institute, Ontario Cancer Institute, Princess Margaret Hospital, University Health Network, Toronto, Ontario, Canada; 3 Department of Medical Biophysics, University of Toronto, Toronto, Ontario, Canada; 4 Samuel Lunenfeld Research Institute, Mount Sinai Hospital, Toronto, Ontario, Canada; University of Cambridge, United Kingdom

## Abstract

Metazoans display remarkable conservation of gene families, including growth factors, yet somehow these genes are used in different ways to generate tremendous morphological diversity. While variations in the magnitude and spatio-temporal aspects of signaling by a growth factor can generate different body patterns, how these signaling variations are organized and coordinated during development is unclear. Basic body plans are organized by the end of gastrulation and are refined as limbs, organs, and nervous systems co-develop. Despite their proximity to developing tissues, neurons are primarily thought to act after development, on behavior. Here, we show that in *Caenorhabditis elegans*, the axonal projections of neurons regulate tissue progenitor responses to Wnts so that certain organs develop with the correct morphology at the right axial positions. We find that foreshortening of the posteriorly directed axons of the two canal-associated neurons (CANs) disrupts mid-body vulval morphology, and produces ectopic vulval tissue in the posterior epidermis, in a Wnt-dependent manner. We also provide evidence that suggests that the posterior CAN axons modulate the location and strength of Wnt signaling along the anterior–posterior axis by employing a Ror family Wnt receptor to bind posteriorly derived Wnts, and hence, refine their distributions. Surprisingly, despite high levels of Ror expression in many other cells, these cells cannot substitute for the CAN axons in patterning the epidermis, nor can cells expressing a secreted Wnt inhibitor, SFRP-1. Thus, unmyelinated axon tracts are critical for patterning the *C. elegans* body. Our findings suggest that the evolution of neurons not only improved metazoans by increasing behavioral complexity, but also by expanding the diversity of developmental patterns generated by growth factors such as Wnts.

## Abstract 2

In *Caenorhabditis elegans*, the axons of specific neurons help direct the location and strength of Wnt signaling that patterns the epidermis.

## Author Summary

How a limited number of conserved growth factors such as Wnts generate diverse bodies throughout the animal kingdom is a fundamental question in developmental and evolutionary biology. Diversity is thought to arise in part through variations in the strength and location of growth factor signaling. How the signaling properties of growth factors are precisely tuned at specific locations to generate distinct tissue patterns is not well understood. Here, we present evidence that the axons of two specific neurons that span the anterior–posterior axis help pattern the epidermis of the nematode *Caenorhabditis elegans*. When the posteriorly directed axons of these neurons fail to grow to their normal length, the symmetry of the mid-body vulva is altered, and additional vulval tissue inappropriately forms in the posterior epidermis. We further present evidence that these neurons direct epidermal patterning by binding and sequestering posteriorly derived Wnts, thereby refining the strength and location of Wnt signaling along the anterior–posterior axis. We postulate that the evolution of neurons not only improved animals by endowing them with complex behaviors, but also by helping expand the diversity of body patterns generated by growth factors.

## Introduction

Metazoan body plans display great morphological diversity in their overall organization and detailed patterns. Yet remarkably, these plans are generated from a small number of conserved growth factors that are reused many times during development to refine the plans in distinct ways. Thus, determining how a limited number of growth factors create different body patterns is key to understanding this diversity. It is known that variations in the magnitude and spatio-temporal aspects of signaling by growth factors and their effectors generate different cellular responses and body patterns [1]–[3]. However, the mechanisms by which a developing animal organizes and coordinates the correct amplitudes of signaling at the right times and places are not well understood.

Wnts comprise one of the oldest families of growth factors, and have conserved functions in organizing and refining body plans. In many metazoan embryos, Wnts are initially expressed from one pole, with their diffusion generating polarity and early patterning of the primary body axis [4]. During and after gastrulation, Wnt gradients, often derived from the posterior body, continue to establish basic identities in the body plan, and then refine these fates to create specific tissue and organ patterns [4]–[6]. As animals develop, it becomes increasingly challenging to spatially organize Wnt activity into the appropriate high and low signaling domains throughout the body. Furthermore, this organization must be coordinated between the multiple Wnts and other growth factors that pattern tissues and organs. Perhaps as a reflection of these challenges, numerous secreted and membrane-associated inhibitors have been identified that help modulate Wnt gradient activity [7]–[10]. However, our knowledge of what a Wnt gradient must look like to pattern a specific limb or organ is very limited, as is our understanding of the distinct roles of the different antagonists.

**Figure 1:**
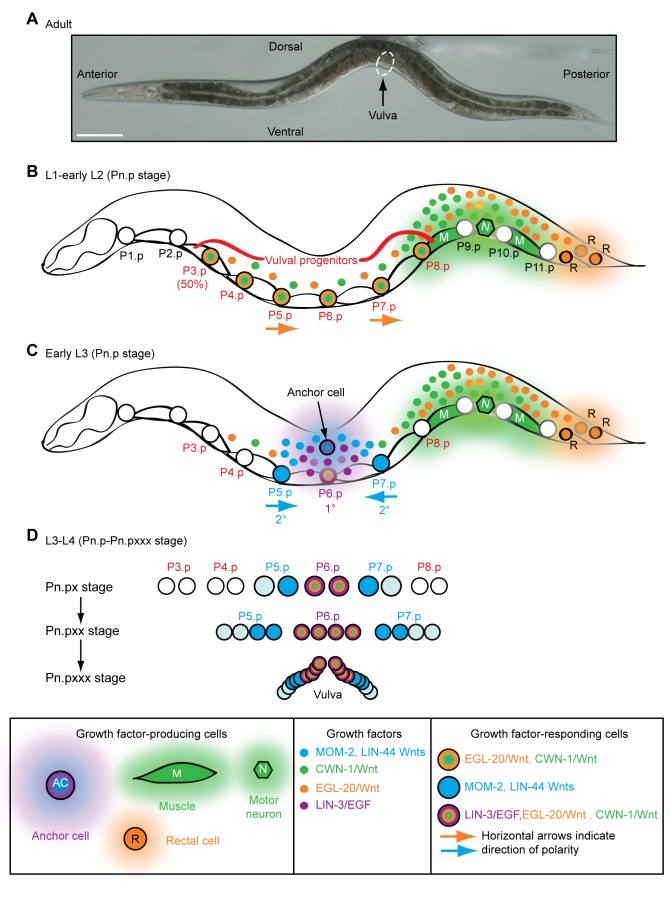
Wnt signaling and epidermal patterning in *C. elegans*. (A) A wild-type *C. elegans* adult hermaphrodite. Scale bar is 100 µm. (B) During the L2 larval stage, LIN-3/EGF from pre-anchor cell/ventral uterine precursor cells (not shown) cooperates with a gradient of EGL-20/Wnt (orange) from rectal cells and CWN-1/Wnt (green) from posterior muscle and neurons to cause six epidermal cells to become vulval progenitors (P3.p–P8.p). 50% of the time, P3.p does not receive sufficient Wnt signaling and adopts the “F” fate (also known as the 4° fate) and fuses with a hypodermal syncytium called hyp7. EGL-20/Wnt also polarizes P5.p and P7.p so that they face posteriorly (horizontal arrows). The epidermal cells normally touch each other, but are drawn apart to facilitate depiction of muscle and neurons. (C) At the end of the L2 larval stage, anchor cell-produced MOM-2 and LIN-44 Wnts (blue) reorient P7.p towards the anterior (horizontal arrows). During the L3 larval stage, LIN-3/EGF (purple) from the anchor cell induces the 1° vulval fate in P6.p, which is facilitated by EGL-20 and CWN-1 Wnts. P5.p and P7.p adopt 2° vulval fates because of the activation of LIN-12/Notch via a lateral signal from P6.p. (D) During the L3–L4 larval stages, vulval progenitor cells (Pn.p) divide to generate Pn.px cells, with P5.p–P7.p undergoing two additional rounds of cell division (to ultimately make Pn.pxxx cells). Because of the opposite polarities of P5.p and P7.p, their asymmetrically dividing progeny generate mirror image patterns. By the early L4 stage, a 22-cell vulva is generated. The Pn.px progeny of P3.p, P4.p, and P8.p fuse with hyp7 (3° fate).

In *Caenorhabditis elegans*, the generation of a vulva in the middle of the anterior–posterior axis has become a paradigm for understanding how Wnt and EGF family growth factors generate specific patterns at precise locations (reviewed in [11]) (Figure 1A). Vulval organogenesis begins with the mid-body generation of six vulval progenitors from 11 blast cells. These progenitors (P3.p–P8.p) are specified by two posteriorly derived Wnt gradients (EGL-20 and CWN-1 [orange and green, respectively, in Figure 1]) [7],[12]–[15], with EGL-20 also polarizing some of the progenitors to face towards the posterior (e.g., P5.p and P7.p) [16] (Figure 1B). Later, mid-body-produced Wnts (LIN-44 and MOM-2 [blue in Figure 1]) reverse P7.p polarity so that P5.p and P7.p face each other and subsequently divide with mirror image symmetry (Figure 1C and 1D) [16],[17]. With the help of the posterior Wnts (EGL-20 and CWN-1), centrally produced EGF (purple in Figure 1) instructs P6.p to adopt a 1° fate, divide three times, and form the vulval lumen that attaches to the uterus (Figure 1C and 1D) [7],[11],[18]–[20]. In parallel, P6.p activates Notch signaling in adjacent P5.p and P7.p to induce 2° fates, which, after three rounds of division, create the symmetrical sides of the vulva that attach the organ to the epidermis (Figure 1C and 1D). Insufficient signaling alters vulval patterning and reduces the amount of vulval tissue [11],[21]. Excessive signaling also alters vulval patterning and, if it occurs at certain axial positions, generates ectopic, non-functional vulvae, which can interfere with normal positioning of muscles and neurons that promote egg-laying [19],[21]–[24].

To understand how growth factors such as Wnts generate specific fates at precise positions, we looked for mutations that affected placement of vulval tissue along the anterior–posterior axis. We were intrigued by mutations in the *vab-8* gene, which affect vulval development through an unknown mechanism and are primarily known for disrupting the migration and axon outgrowth of a few neurons [25],[26]. While nervous systems co-develop with tissues and organs [6], with only rare exceptions, their importance in refining body plans has been unexplored. In flies, through unknown mechanisms, motor neurons contribute to abdominal and flight muscle patterning [27],[28], and in mammals, by secreting VEGF, sensory nerves direct arterial patterning in skin [29]. In addition, we previously discovered that in *C. elegans*, motor neuron excitation stimulates vulval fate signaling [30]. Thus, we were interested in exploring the possibility that neurons might refine patterning by widely used growth factors such as Wnts.

Here we show that *C. elegans* has evolved a neuronal-based mechanism to refine the amplitude and spatial signaling properties of the posterior-derived Wnt gradients that pattern the epidermis. Two canal-associated neurons (CANs), whose axons span the anterior–posterior axis, ensure that a vulva is generated with the correct morphology and only at the mid-body. When outgrowth of the posterior CAN axon is severely shortened, Wnt signaling is increased along the anterior–posterior axis, especially in the posterior body. This deregulated signaling alters the symmetry of the normal mid-body vulva, and causes ectopic vulval tissue to form in the posterior epidermis. Finally, we provide evidence that although the Ror/CAM-1 Wnt receptor is widely expressed, its expression in the CAN axons is part of a unique Wnt-sequestration mechanism that ultimately directs the locations and strength of Wnt signaling necessary for proper epidermal patterning.

## Results

### Mutations Affecting Neuronal Migration and Axon Outgrowth Affect the Symmetry and Axial Position of Vulval Tissue

**Figure 2:**
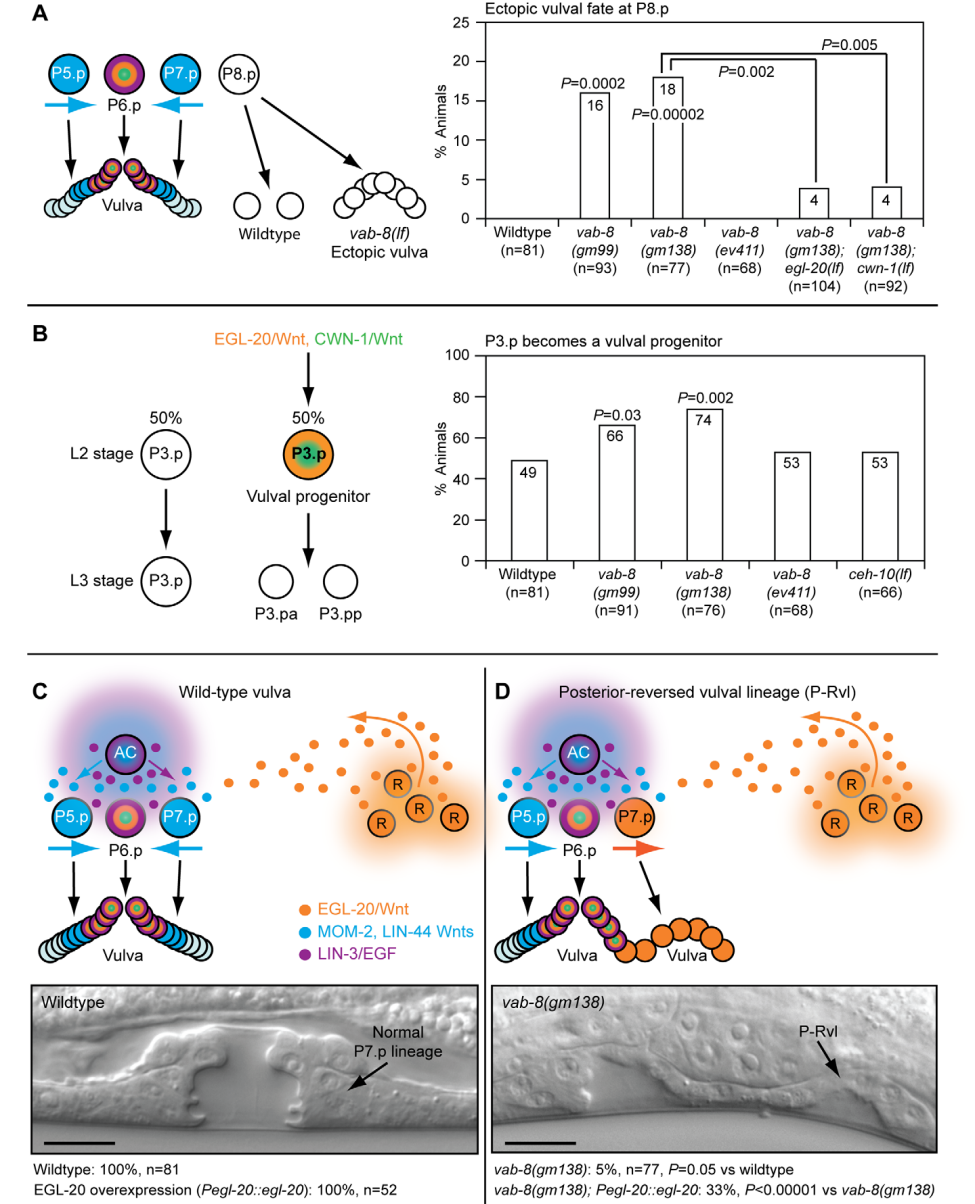
*vab-8* mutations that affect neuronal cell body positioning and axon outgrowth cause epidermal patterning defects. (A) *vab-8* mutations cause P8.p to adopt a vulval fate. In wild-type animals, P8.p divides once, but never forms vulval tissue. (B) *vab-8* mutations increase the frequency of P3.p becoming a vulval progenitor. 50% of the time, P3.p receives sufficient Wnt signaling to become a vulval progenitor and divides once. (C) Upper panel depicts wild-type signaling by Wnts and EGF that promotes vulval development with mirror image symmetry. MOM-2 and LIN-44 Wnts dominate over EGL-20/Wnt to polarize P7.p towards the anterior. Lower panel shows a wild-type 22-cell vulva with normal symmetry at the mid-L4 stage. (D) Upper panel depicts abnormal Wnt signaling in *vab-8* mutants that causes the formation of vulval tissue with a P-Rvl phenotype. EGL-20/Wnt dominates over MOM-2 and LIN-44 Wnts, preventing P7.p from reorienting towards the anterior. Lower panel shows a P-Rvl vulva at the mid-L4 stage. In (C) and (D), EGL-20/Wnt was overexpressed from its native promoter with the *muIs49* transgene. Scale bar is 10 µm. Colors depict Wnt signaling as in Figure 1. *p-*Values were calculated using a two-tailed Fisher's exact test versus wild-type animals (A and B) or as otherwise indicated (A and D).

*vab-8* encodes a long isoform, VAB-8L, and several short isoforms collectively called VAB-8S [31]. These proteins act in a few neurons to promote their posterior-directed migration and axon outgrowth [31],[32]. Both isoforms possess C-terminal coiled-coil domains, with VAB-8L having an additional N-terminal kinesin domain. In wild-type animals, only the three central vulval progenitors (P5.p–P7.p) adopt vulval fates. However, in *vab-8(gm99)* and *vab-8(gm138)* mutants, which lack both isoforms and have anteriorly displaced neurons, the posterior P8.p progenitor also acquired a vulval fate (Figure 2A). In the anterior epidermis of wild-type animals, Wnt signaling is limiting in P3.p because of its distance from the posterior Wnts; therefore, it becomes a progenitor only 50% of the time (Figure 1B and 2B). *vab-8* mutations did not cause ectopic vulval fates in anterior P3.p or P4.p (*n = *170), but they increased the frequency with which P3.p became a vulval progenitor (Figure 2B). In the mid-body, at low frequency, *vab-8* mutations disrupted the mirror image symmetry of the vulva. Initially, during the generation of the vulval progenitors in wild-type animals, posterior-derived EGL-20/Wnt causes P5.p and P7.p to polarize and face towards the posterior (Figure 1B) [16]. Later, centrally produced MOM-2 and LIN-44 Wnts maintain P5.p polarity towards the posterior, but cause P7.p to reorient and face towards the anterior (Figures 1C and 2C) [16],[17]. *vab-8* mutations caused a “posterior-reversed vulval lineage” (P-Rvl) phenotype, in which P7.p remained polarized towards the posterior EGL-20/Wnt signal, so that its subsequent cell divisions caused a second vulval invagination (Figure 2D).

To study how VAB-8-regulated cells affect epidermal development, we used mutant backgrounds permitting sensitive quantification of the effects of *vab-8* mutations. A loss-of-function *egfr/let-23(lf)* mutation severely diminishes the 1° fate response in P6.p such that fewer than the normal three progenitors adopt vulval fates, causing an “underinduced” or “vulvaless” phenotype. This mutant phenotype can be suppressed by activating the EGFR pathway downstream of the receptor (e.g., by a loss-of-function mutation in the Ras/LET-60 inhibitor *gap-1* [33]) or by mutations that increase signaling by the parallel Wnt pathway (e.g., a loss-of-function mutation in the Wnt pathway inhibitor *axin/pry-1* [19]) (Table 1). By counting total vulval progeny, the extent to which any progenitor adopts a vulval fate can be quantified. *vab-8(gm99)* and *vab-8(gm138)* mutations suppressed the underinduced phenotypes of loss-of-function *egf/lin-3* and *egfr/let-23* alleles and of a dominant-negative *ras/let-60(dn)* mutation (Table 1). Since increasing EGFR activity above wild-type levels does not suppress the *ras/let-60(dn)* mutation [30], VAB-8-regulated cells must modulate a signal that is distinct from EGF. Based on the posterior bias in the induction of ectopic vulval fates, the P-Rvl phenotype (which can result from overactive EGL-20 signaling), and the increased P3.p progenitor frequency (which is normally regulated by EGL-20 and CWN-1), this signal(s) could include one of the posteriorly enriched Wnts such as EGL-20 or CWN-1.

**Table 1:** Mutations impairing CAN neuron migration and axon outgrowth affect epidermal development.

Genotype	Vulval Fates^a^	*n* b	*p-*Value^c^
Wild-type	3.00	47	
*gap-1(lf)*	3.00	20	
*pry-1(lf)*	3.42^d^	57	
*vab-8(gm99)*	3.19	21	
*vab-8(gm138)*	3.22	77	
*vab-8(ev411)*	3.00	68	
*ceh-10(lf)*	3.08	87	
*sfrp-1(lf)*	3.00	53	
*let-23(lf)*	0.52	24	
*let-23(lf); gap-1(lf)*	4.00	40	<0.00001 versus *let-23(lf)*
*pry-1(lf); let-23(lf)*	4.00	25	<0.00001 versus *let-23(lf)*
*let-23(lf); vab-8(gm99)*	1.85	24	0.0002 versus *let-23(lf)*
*let-23(lf); vab-8(gm138)*	1.95	20	0.0001 versus *let-23(lf)*
*let-23(lf); vab-8(ev411)*	0.66	40	0.54 versus *let-23(lf)*
*let-23(lf); ceh-10(lf)*	0.64	21	0.65 versus *let-23(lf)*
*let-23(lf); sfrp-1(lf)*	0.05	21	
*lin-3(lf)*	0.91	46	
*lin-3(lf); vab-8(gm99)*	2.09	20	0.002 versus *lin-3(lf)*
*lin-3(lf); vab-8(gm138)*	2.12	46	<0.00001 versus *lin-3(lf)*
*lin-3(lf); vab-8(ev411)*	0.80	27	0.64 versus *lin-3(lf)*
*ceh-10(lf); lin-3(lf)*	0.85	34	0.78 versus *lin-3(lf)*
*let-60(dn)/+* e	1.07	21	
*let-60(dn)/+; vab-8(gm99)* e	2.30	20	0.0005 versus *let-60(dn)*
*let-60(dn)/+; vab-8(gm138)* e	2.48	20	0.00005 versus *let-60(dn)*

a Vulval fates: number of vulval progenitor cells adopting vulval fates.

b *n*: number of animals assayed.

c *p-*Values were calculated using a two-tailed Student's *t* test.

d This number of vulval fates is an approximation, since in this background it is difficult to count the exact number of vulval cells descended from P9.p and P10.p.

e The *let-60* mutation was linked to *unc-24(lf)* and balanced by *dpy-20(lf)*.

*dn*, dominant-negative; *lf*, loss-of-function.

### The CAN Neurons Regulate Epidermal Patterning

**Figure 3:**
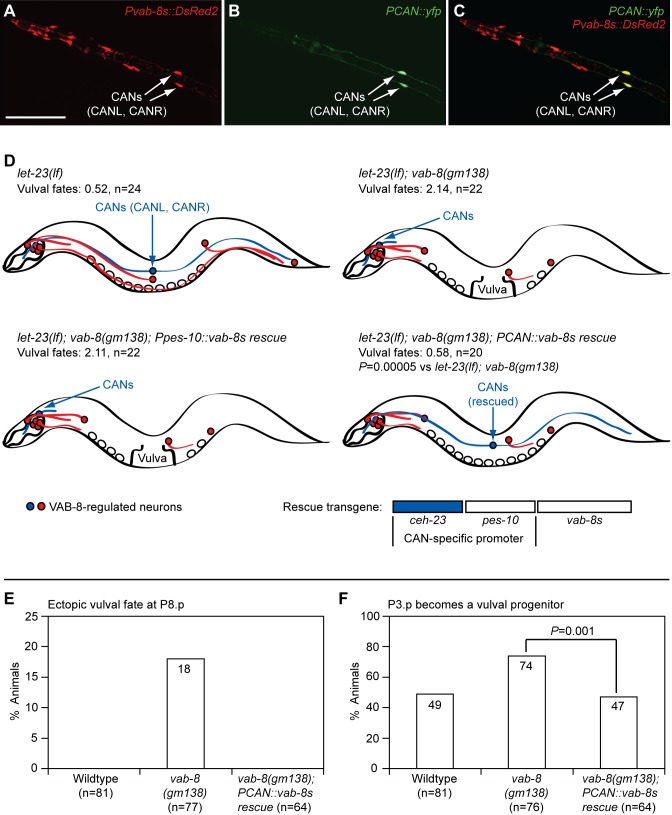
VAB-8 acts in the CAN neurons to regulate epidermal patterning. (A–C) Expression of *vab-8s* and *CAN* promoters from the *akEx923* transgenic array in wild-type L2 stage animals. Scale bar is 50 µm. The CANs are a pair of neurons, CANL and CANR, located on the left and right side of each animal, respectively. (A) DsRed2 channel. (B) YFP channel. (C) Merged images from (A) and (B). (D) CAN-specific expression of VAB-8S restores inhibition to vulval fate signaling in *vab-8* mutants. *vab-8* mutations displace cell bodies and affect axon outgrowth of a subset of neurons including the CANs (blue). VAB-8S was restored to *vab-8* mutants with either the CAN-specific promoter (*PCAN*) or the control minimal *pes-10* promoter (*Ppes-10*). Vulval fates: number of vulval progenitor cells adopting vulval fates. Wild-type is 3.00. *p-*Value was calculated using a two-tailed Student's *t* test. Transgenic arrays were *dyEx24* (*Ppes-10::vab-8s*) or *dyEx20* (*PCAN::vab-8s*). CAN-specific expression of VAB-8S from the *dyEx20* transgenic array rescues patterning defects in the posterior and anterior epidermis (E and F). *p-*Value was calculated using a two-tailed Fisher's exact test.

In contrast to mutations that disrupt both VAB-8 isoforms, the *vab-8(ev411)* mutation, which only affects VAB-8L [31], did not cause ectopic vulval fates in P8.p, did not increase the frequency of P3.p becoming a vulval progenitor, and did not suppress the underinduced phenotypes of *egf/lin-3* or *egfr/let-23* mutants (Figure 2A and 2B; Table 1). Thus, VAB-8S is sufficient to inhibit vulval fates. To identify cells whose position might influence epidermal development, we examined promoter activity in the smallest genomic fragment previously shown to encode functional VAB-8S activity [31]. This promoter drove reporter expression in eight head neurons and the pair of CANs (CANL and CANR) (Figure 3A). To determine whether any of these neurons regulate epidermal development, we used this promoter to restore VAB-8S (*Pvab-8s::vab-8s*) to all of these cells in *egfr/let-23(lf); vab-8(gm138)* double mutants. VAB-8S expression in these cells fully restored the *egfr/let-23(lf)* vulvaless phenotype (Table S1), suggesting that the positioning of one or more of these cells inhibits vulval fate signaling in P6.p. We then focused on the CANs, since (1) of the ten neurons, only the CAN axons span the entire anterior–posterior axis, (2) *vab-8* null mutations severely displace CAN cell bodies and shorten posterior axons [26], and (3) the *vab-8(ev411)* mutation, which does not affect epidermal development, does not affect CAN cell body position, and only weakly affects CAN axon outgrowth [26]. Since VAB-8 acts cell autonomously in the CANs to promote their posterior migration and axon outgrowth [31],[32], we used a previously described CAN-specific enhancer [34] to create an expression vector to specifically restore VAB-8S and proper positioning only to the CANs. This vector drove YFP expression only in the CANs, which was also the only site of co-localization with *vab-8s*-driven *DsRed2* (Figure 3B and 3C), confirming the cell-specificity of this vector. When expressed from the CAN-specific promoter (*PCAN::vab-8s*), but not the control minimal *pes-10* promoter, VAB-8S also restored full inhibition to vulval fate signaling in P6.p in *egfr/let-23(lf); vab-8(gm138)* double mutants (Figure 3D; Table S1). This transgene also fully suppressed the other *vab-8(gm138)* epidermal phenotypes, including the ectopic vulval fates at P8.p (Figure 3E), the increased frequency of P3.p becoming a vulval progenitor (Figure 3F), and the P-Rvl phenotype (0%, *n = *64). Thus, VAB-8 acts in the CANs to regulate epidermal development.

Since the CANs have been implicated in the control of osmotic balance [35],[36], we ruled out the CANs indirectly modulating epidermal development through regulation of animal physiology. Experiments involving direct osmotic stress, disruption of the osmoregulatory system, and mutation of the osmotic-stress-responsive *p38 mapk/pmk-1* did not support this model (Text S1; Tables S2 and S3; Figure S1).

### The CANs Inhibit EGL-20/Wnt Activity

**Figure 4:**
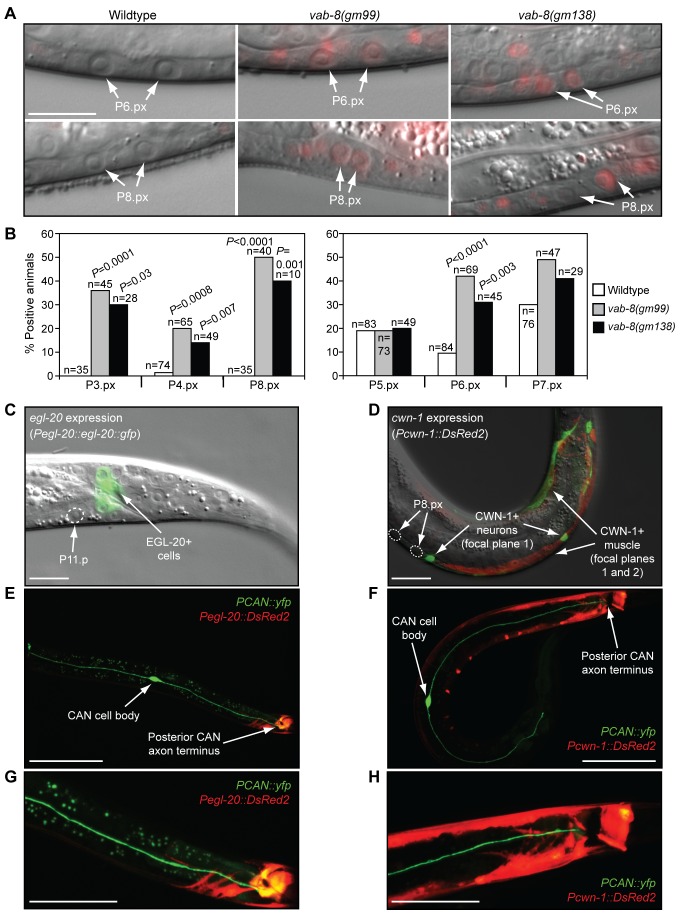
CAN neurons inhibit Wnt signaling in epidermal progenitors. (A) Pn.px stage animals showing *syIs187* mCherry Wnt reporter activity. Scale bar is 20 µm. (B) Quantification of reporter data. Reporter activity was lower in P5.px–P7.px cells than in P3.px, P4.px, and P8.px cells, so data were collected using a higher brightness setting. *p-*Values were calculated using a two-tailed Fisher's exact test versus wild-type animals. (C and D) Location of *egl-20/wnt-* and *cwn-1/wnt*-expressing cells relative to epidermal cells in L3, Pn.px stage animals. Scale bar is 10 µm. (C) *muIs49[Pegl-20::egl-20::gfp]* transgenic animal. (D) *dyEx10[Pcwn-1::DsRed2]* transgenic animal. To simultaneously visualize neurons and muscle, images were taken in different focal planes, differentially colored either green or red, and merged. (E–H) Location of Wnt-producing cells relative to CAN neurons. Only one CAN cell body is visible. (G and H) Blow-up of (E) and (F), respectively. Scale bars are 50 µm (E and F) and 25 µm (G and H). Transgenic arrays were *akEx906* (E and G) and *akEx908* (F and H).

Since our genetic and phenotypic analyses suggested the CANs inhibit Wnt activity, we directly examined whether CAN displacement might increase Wnt activity in epidermal progenitors [16]. An mCherry-based Wnt reporter has been described that specifically reflects Wnt signaling in epidermal progenitors beginning after their first division (Pn.px stage) and extending through their second division (Pn.pxx) (Figure 1D) [16]. In *axin/pry-1* Wnt inhibitor mutants, both the frequency and intensity of reporter activity was increased in these cells (Figure S2A–S2C). Although *vab-8(gm99)* and *vab-8(gm138)* mutations did not increase Wnt reporter intensity as dramatically as an *axin/pry-1* mutation, they did cause more animals to show reporter activity in P3.px, P4.px, P6.px, and P8.x progeny (Figure 4A and 4B). These data suggest that the CANs dampen Wnt signaling along much of the anterior–posterior axis, and that deregulation of this signaling might account for the epidermal patterning defects observed in *vab-8* mutants.

In wild-type animals, mutation or RNA interference (RNAi) of individual Wnt genes did not reduce the normal frequency of vulval fates (Table 2). However, mutation of *egl-20/wnt* or RNAi of *cwn-1/wnt* or *mom-2/wnt* abrogated the ability of a *vab-8(lf)* mutation to suppress the underinduced phenotype of *egfr/let-23(lf)* mutants, suggesting one or more of these Wnts is a target of the CANs (Table 2). Sensitivity to the levels of these Wnts was specific, as mutations in the *cwn-2* and *lin-44* Wnt genes did not cause comparable effects (Tables 2 and S4; Text S1). Individual mutations in either *egl-20/wnt* or *cwn-1/wnt* also strongly reduced ectopic vulval fates at P8.p, suggesting that one or more of these Wnts are targeted by the CANs (Figure 2A).

**Table 2:** CAN displacement causes epidermal phenotypes that require signaling by specific Wnts and that resemble phenotypes caused by mutations in the Wnt-binding Ror/CAM-1 receptor.

Genotype	Vulval Fates^a^	*n* b	*p-*Value^c^
Wild-type	3.00	47	
*egl-20(lf)*	3.00	58	
*cwn-2(lf)*	3.00	25	
*lin-44(lf)*	3.00	20	
Wild-type; *vector RNAi*	3.00	20	
Wild-type; *cwn-1 RNAi*	3.00	20	
Wild-type; *mom-2 RNAi*	3.00	20	
*let-23(lf); vab-8(gm138)*	1.95	20	0.0001 versus *let-23(lf)*
*let-23(lf); egl-20(lf); vab-8(gm138)*	0.68	20	0.001 versus *let-23(lf); vab-8(gm138)*
*lin-44(lf); let-23(lf); vab-8(gm138)*	2.15	20	0.62 versus *let-23(lf); vab-8(gm138)*
*let-23(lf); cwn-2(lf); vab-8(gm138)*	1.30	25	0.07 versus *let-23(lf); vab-8(gm138)*
*let-23(lf); vab-8(gm138); vector RNAi*	1.45	20	
*let-23(lf); vab-8(gm138); cwn-1 RNAi*	0.40	31	0.005 versus *let-23(lf); vab-8(gm138); vector RNAi*
*let-23(lf); vab-8(gm138); mom-2 RNAi*	0.63	23	0.04 versus *let-23(lf); vab-8(gm138); vector RNAi*
*let-23(lf); syEx1024[Phs::egl-20]*	0.13	20	
*let-23(lf); syEx1024[Phs::egl-20]* heat shock^d^	1.70	20	<0.00001 versus non-transgenic heat shock
Control non-transgenic sibling^e^	0.00	20	
Control non-transgenic sibling heat shockd,e	0.05	20	
*cam-1(gm122)*	3.04	51	
*cam-1(sa692)*	3.03	74	
*cam-1(ks52)*	3.00	62	
*lin-3(lf)*	0.91	46	
*cam-1(gm122); lin-3(lf)*	1.76	44	0.0004 versus *lin-3(lf)*
*cam-1(sa692); lin-3(lf)*	2.08	31	<0.00001 versus *lin-3(lf)*
*cam-1(ks52); lin-3(lf)*	1.11	27	0.47 versus *lin-3(lf)*

a Vulval fates: number of vulval progenitor cells adopting vulval fates. Wild-type is 3.00.

b *n*: number of animals assayed.

c *p-*Values were calculated using a two-tailed Student's *t* test.

d Mixed-stage animals were heat-shocked at 33°C for 45 min and scored 16 h later.

e Control animals were of the same genotype as the transgenic animals, but lacked the extrachromosomal transgenic array.

*lf*, loss-of-function.

During vulval development, *egl-20/wnt* expression was detected in four rectal cells (K, F, B, and U) just posterior to P11.p (Figure 4C) [15]. *cwn-1/wnt* was strongly expressed in posterior body-wall muscle and a subset of posterior motor neurons (Figure 4D) [13],[37]. These cells were posterior to the P8.px progeny (Figure 4D), but weakly expressing neurons and muscle extended anteriorly to P2.p. Strikingly, the CANs also expressed CWN-1/Wnt (Figure S3) [38], and their posterior axons traveled parallel to the epidermal progenitors and the other CWN-1-producing cells, and terminated at the sources of EGL-20/Wnt (Figure 4E–4H). Thus, the CANs appear well-positioned to regulate epidermal progenitor responses to these Wnts.

To further investigate the possibility that the CANs target EGL-20/Wnt, we examined hermaphrodite-specific neuron (HSN) migration in *vab-8* mutants. The two HSNs are born in the posterior of the embryo, close to the EGL-20/Wnt-producing cells, and migrate to the mid-body position of the prospective vulva [39] (Figure S4A and S4B). *egl-20/wnt* mutations prevent this migration, while mutations in other Wnt genes have little effect [40],[41]. Conversely, increased EGL-20/Wnt activity causes anterior overmigration of the HSNs [23], implying that an EGL-20 gradient drives correct mid-body placement of the HSNs. Consistent with prior reports [26],[35],[42], we found that in *vab-8* mutants HSNs overmigrated anteriorly (Figure S4C), suggesting EGL-20/Wnt activity is increased along the anterior–posterior axis. This phenotype was enhanced by increasing EGL-20/Wnt levels with an integrated transgenic array that carries additional copies of the genomic *egl-20* locus (Figure S4D and S4E) [23],[40]. Since laser ablation of the CAN precursors also causes HSN overmigration [35], the effect of *vab-8* mutation on HSN migration is likely mediated by the CANs. The integrated *egl-20/wnt* transgenic array also cooperated with the *vab-8(gm138)* mutation to increase the incidence of the P-Rvl phenotype (Figure 2C and 2D), and to cause an embryonic posterior P11 to P12 blast fate transformation, which can occur when either EGF [43] or Wnt signaling activity is abnormally high (Figure S4F and S4G). The P11 fate transformation in *vab-8* mutants is also at least partly mediated by the CANs, since CAN-specific restoration of VAB-8S significantly rescued this phenotype (Figure S4G).

Since elevated EGL-20/Wnt activity specifically causes a P-Rvl phenotype, and increased activity of either EGL-20 or CWN-1/Wnt could, in principle, account for the increased conversion of P3.p into a vulval progenitor [7],[14],[16], we asked whether elevation of one of these Wnts could also account for how *vab-8* mutations suppress the underinduced phenotype of an *egfr/let-23(lf)* mutation. When expressed from a heat-shock-regulated transgene (*Phs::egl-20*), EGL-20/Wnt suppressed the *egfr/let-23(lf)* mutation comparably to *vab-8* mutations (Table 2). However, this transgenic array did not induce ectopic vulval fates at P8.p in wild-type animals (*n = *52). This may be due to a lack of sufficiently high EGL-20/Wnt expression in the posterior epidermis and/or a need to increase signaling by additional Wnts such as CWN-1.

In agreement with the idea that Wnt signaling sufficient for the induction of ectopic vulval fates is still limiting after heat shock of just EGL-20, mutation of the intracellular Wnt inhibitor *axin/pry-1* caused very high Wnt reporter activity in vulval progenitor progeny, fully suppressed the underinduced phenotype of *egfr/let-23* mutants, and caused ectopic vulval fates (Table 1; Figure S2) [19]. This high level of Wnt signaling also caused P9.p and P10.p to become vulval progenitors, which, along with P8.p, formed most of the ectopic vulval tissue (Figure S2D and S2E). Together, our combined data suggest that EGL-20/Wnt is one CAN target, and that additional Wnts, including CWN-1, may also be inhibited by the CANs.

Anterior displacement of CAN cell bodies and foreshortening of the posterior axons caused a gradual post-embryonic withering of the posterior half of the animal [42], which placed some epidermal progenitors closer to the anus, where EGL-20/Wnt is produced (Figure S5A), and reduced posterior body volume (Figure S6A–S6C). We ruled out these physical changes as being the major mechanism by which the CANs regulate epidermal development (see Text S1). In *vab-8* mutants, Wnt reporter activity in vulval progenitors did not strictly correlate with their distance from the anus (Figure S5B); *dpy-17(lf); dpy-20(lf)* cuticular mutants had normal CAN positioning and axon outgrowth, but even shorter epidermal progenitor distances to the anus and smaller posterior body volumes than *vab-8* mutants (including *vab-8* mutants with ectopic vulval fates), yet did not show epidermal phenotypes (Figures S5C–S5F, S6D, and S6E; Table S3); *vab-8* mutations and ablation of the CAN precursors promoted embryonic EGL-20/Wnt-dependent HSN overmigration prior to any tail withering (Figure S4B–S4E) [26],[35],[42]; and the CANs also prevented overexpressed EGL-20/Wnt from causing embryonic P11 to P12 fate transformations, before tail withering occurred (Figure S4G).

### The Posterior CAN Axon Inhibits Wnt Activity

**Figure 5:**
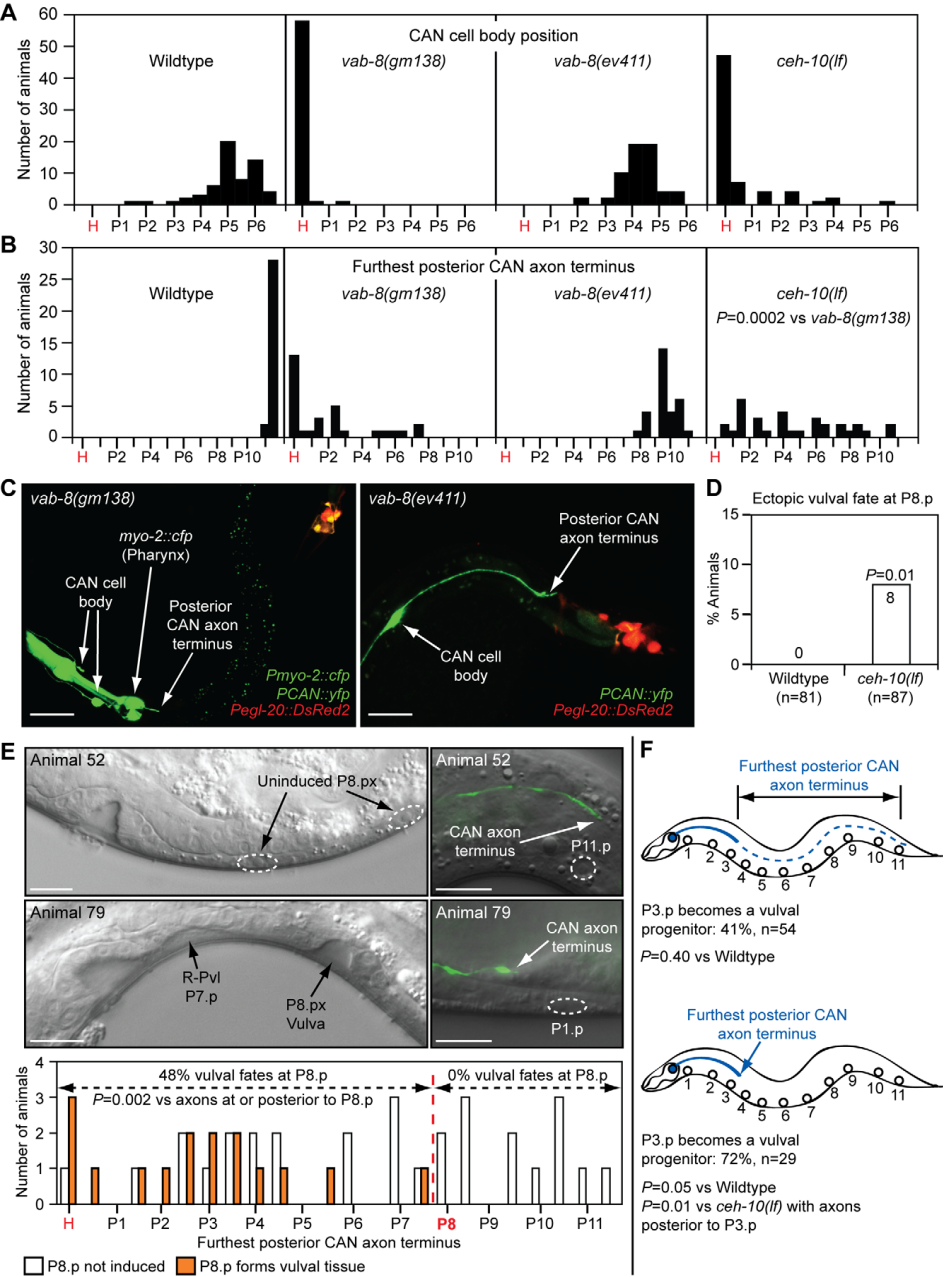
Posterior CAN axons regulate axial positioning of vulval fates and P3.p vulval progenitor frequency. Distributions of positions of CAN cell bodies (A) and furthest posterior CAN axon termini (B) in different mutants. CAN neurons were visualized in L3, Pn.px stage (A and B) or L4 stage (E) animals with the *kyIs4[Pceh-23::gfp]* transgene. *x*-Axis indicates Pn.p or Pn.px positions. *p-*Value was calculated using a two-tailed Mann-Whitney *U* test. (C) Position of CAN cell bodies and posterior axon termini relative to *egl-20/wnt*-expressing cells in L2 stage *vab-8* mutants. CANs and *egl-20/wnt*-expressing cells were marked with the *akEx906* transgenic array. The bright green signal in the head/pharynx is from the coinjected *Pmyo-2::cfp* injection marker. Scale bar is 25 µm. (D) Frequency with which P8.p adopts a vulval fate in the total *ceh-10* mutant population. (E) Correlation between the position of the furthest posterior CAN axon terminus and induction of ectopic vulval fates at P8.p in *ceh-10(lf)* mutants. For this study, an emphasis was placed on picking smaller animals to ensure that sufficient numbers of animals with short posterior CAN axons were obtained for statistical analysis. Thus, the combined frequency of ectopic vulval fates in this study is not an estimate of the actual frequency in the total population as conducted in (D). Top panels show animal 52, with normal epidermal development and normal position of furthest posterior CAN axon terminus. Bottom panels show animal 79, with an R-Pvl phenotype at P7.p and ectopic vulval fate at P8.p, and severely foreshortened furthest posterior CAN axon terminus. Scale bars are 10 µm. (F) Correlation between position of furthest posterior CAN axon terminus and frequency with which P3.p becomes a vulval progenitor in *ceh-10(lf)* mutants. In (D–F), *p-*Values were calculated using a two-tailed Fisher's exact test versus wild-type animals (D) or as otherwise indicated (E and F). H, head/pharyngeal region.

While in wild-type animals the median CAN cell body position along the anterior–posterior axis was near the P5.px progeny, and most posterior CAN axons terminated near P11.p and the EGL-20/Wnt-producing rectal cells (Figures 4E, 4G, 5A, and 5B), in *vab-8(gm138)* mutants, both the CAN cell bodies and posterior axon termini were severely displaced anteriorly (Figure 5A–5C). Given the normal proximity of the posterior CAN axon terminus to the EGL-20/Wnt-producing cells, the axons may secrete a short-range signal that inhibits EGL-20 production. However, severe mutation of *vab-8* did not increase *egl-20/wnt* RNA levels (Figure S7), and the *vab-8(ev411)* mutation, which mildly shifted the posterior CAN axon terminus away from the EGL-20/Wnt-producing cells (Figure 5B and 5C), did not cause epidermal phenotypes (Figure 2A and 2B; Table 1).

We next examined how a *ceh-10* homeobox gene mutation that has intermediate effects on posterior CAN axon outgrowth affects epidermal development [35]. Although in *ceh-10* mutants the median CAN cell body position was anteriorly shifted to the head, similarly as in *vab-8(gm138)* mutants (Figure 5A) [35], the posterior CAN axon extended further than in *vab-8* mutants, to a median position of P4.px rather than P1.p (Figure 5B). Mutation of *ceh-10* did not increase the frequency of P3.p becoming a vulval progenitor (Figure 2B), and did not suppress the P6.p-based underinduced phenotypes of *egf/lin-3* or *egfr/let-23* mutations (Table 1). However, *ceh-10* mutants had a low incidence of ectopic vulval fates in P8.p (Figure 5D). These data suggest that a rarer aspect of the *ceh-10* mutant phenotype, such as severe shortening of the posterior CAN axon rather than displacement of the cell body, may promote vulval fate signaling along the anterior–posterior axis.

To determine the relationship between the posterior CAN axon terminus and vulval fate signaling, we examined both parameters in individual *ceh-10* mutants. In animals where the furthest posterior CAN axon terminus of the pair of neurons was at or posterior to P8.p progeny, P8.p did not adopt a vulval fate (Figure 5E, e.g., animal 52). By contrast, in animals where the furthest posterior CAN axon terminus was anterior to P8.p progeny, P8.p acquired ectopic vulval fates, with some animals also showing a P-Rvl phenotype in P7.p (Figure 5E, e.g., animal 79). We also identified rare *ceh-10* mutants that demonstrated a separation between tail withering and increased vulval fate signaling. In animals 79, 74, and 45, the Wnt-responding P8.px progenitors that gave rise to ectopic vulvae were further away from the EGL-20/Wnt sources (>135 µm) than the non-Wnt-responding P8.px progenitors in animals not having ectopic vulval induction (135 µm) (Figure S8). However, in these three animals, the posterior CAN axon never extended beyond P3.px, demonstrating that productive vulval fate signaling is always correlated with severe posterior axon shortening.

To determine whether a similar relationship also exists between the posterior CAN axon terminus and the signaling involved in converting anterior P3.p into a vulval progenitor, we also examined these two parameters in *ceh-10* mutants. Analogous to the study at P8.p, we found that when the longest of the pair of posterior CAN axons reached only the P3.p position, the frequency of P3.p becoming a progenitor increased from the normal ∼50% to 72% (Figure 5F). This increased frequency is similar to that in *vab-8* mutants, which have a median furthest posterior axon terminus position of P1.p. In contrast, when the longest posterior CAN axon terminated past P3.p, the frequency of P3.p becoming a progenitor was 41%, which was not statistically different from that of wild-type animals. Together, these data indicate that foreshortening of the posterior CAN axons can cause epidermal progenitors to show evidence of enhanced responses to Wnt signaling.

Although anterior and posterior epidermal progenitor responses to Wnts are inversely correlated with the length of the posterior CAN axon, the striking expression of CWN-1/Wnt in the CAN neurons suggests the possibility that when anteriorly displaced, the cell bodies of these neurons might promote Wnt signaling in anterior epidermal cells such as P3.p. Consistent with this model, we found that the increased frequency of P3.p becoming a vulval progenitor in *vab-8* mutants was more dependent on *cwn-1* than *egl-20/wnt* activity (Figure S9A). If this strong dependence on CWN-1/Wnt was largely due to increased proximity of CWN-1-producing CAN cell bodies to P3.p, it would be expected that CAN cell bodies would be consistently closer to P3.p when P3.p becomes a vulval progenitor. We found 17 *ceh-10* mutants to test this hypothesis. In these mutants, at least one of the cell bodies of the pair of CAN neurons was displaced closer to P3.p, away from its normal median position of P5.p (Figure S9B). However, regardless of whether P3.p had or had not become a vulval progenitor, the cell body that was closest to P3.p (among the pair of CAN neurons) was similarly close to P3.p (Figure S9C, left panel). Also arguing against a positive role for the CAN cell bodies in promoting Wnt signaling in P3.p, we found that when P3.p became a vulval progenitor, the cell body that was furthest from P3.p (among the pair of CAN neurons) was significantly further away from P3.p than in cases where P3.p did not become a progenitor (Figure S9C, right panel). In fact, in one case, we found an animal where the two CAN cell bodies were directly over P3.p, yet P3.p still failed to become a vulval progenitor (Figure S9B, animal 92). Notably, in this animal, both posterior CAN axons terminated past the P8.p progeny. The most significant correlation we noticed in animals with at least one of the two CAN cell bodies displaced closer to P3.p was that when P3.p became a vulval progenitor, fewer posterior CAN axons reached P3.p (Figure S9B). Collectively, these results indicate that the key role of the CANs in epidermal development is to inhibit Wnt signaling, and that the posterior CAN axons must extend a certain distance to confer this inhibition.

### The CAN Axons Use the Ror/CAM-1 Wnt Receptor to Regulate Wnt Signaling in Epidermal Progenitors

**Figure 6:**
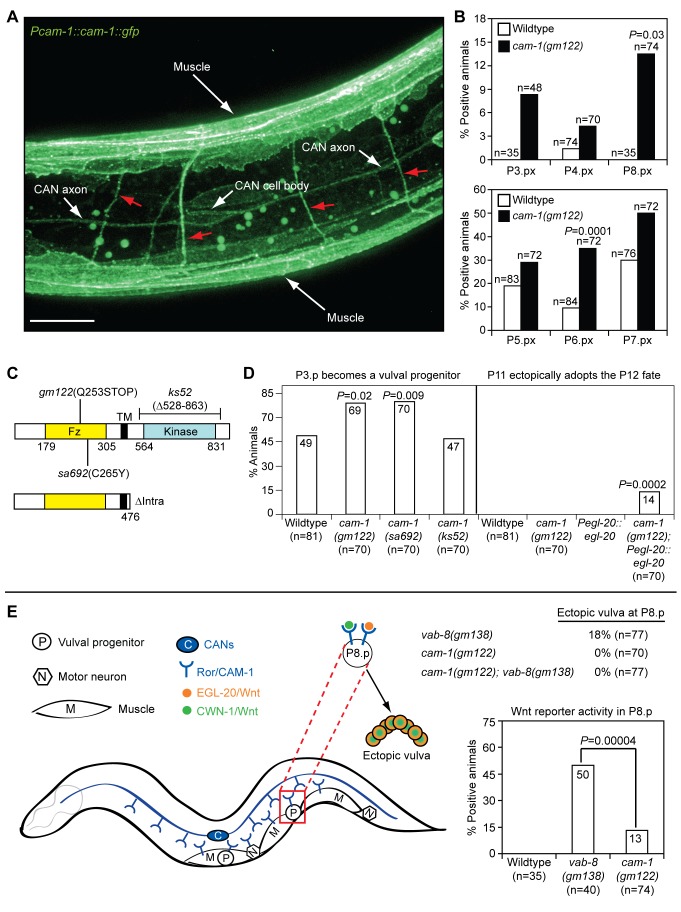
Ror/CAM-1 has negative and positive effects on vulval fate signaling. (A) Ror/CAM-1 is broadly expressed. L2 stage animal with the *cwIs6* transgenic array, which expresses a rescuing Ror/CAM-1 translational fusion to GFP from the *cam-1* promoter. Red arrows indicate axons of non-CAN neurons. Scale bar is 10 µm. (B) Ror/CAM-1 inhibits Wnt signaling in epidermal progenitors. Quantification of *syIs187* Wnt reporter transgene activity in Pn.px stage *ror/cam-1* mutants. Pn.px signals were scored as in Figure 4B. (C) Schematic description of *ror/cam-1* mutations and the *cam-1* transgenic mutant (ΔIntra) used in this study. Numbers indicate amino acid positions. Fz, Frizzled, Wnt-binding extracellular domain; TM, transmembrane domain. (D) Ror/CAM-1 inhibits P3.p from becoming a vulval progenitor in larvae, and helps restrict the Wnt-dependent P12 fate to only the P12 blast cell in embryos. *Pegl-20::egl-20* is an integrated transgenic array that introduces extra functional copies of *egl-20/wnt* fused to *gfp* into the genome (see Figures S4 and S7). (E) Ror/CAM-1 is required for foreshortening of the posterior CAN axon to promote a vulval fate in the posterior P8.p progenitor. Model depicting negative and positive roles of Ror/CAM-1 on Wnt signaling in epidermal progenitors. *p-*Values were calculated using a two-tailed Fisher's exact test versus wild-type animals (B and D) or as otherwise indicated (E).

The *C. elegans* genome encodes one diffusible Wnt antagonist, SFRP-1 [38]. However, SFRP-1 does not mediate the effects of the CANs on epidermal development: its expression is largely restricted to anterior body wall muscle [38], and unlike CAN-displacing *vab-8* mutations, mutation of *sfrp-1* did not suppress the underinduced phenotype of *egfr/let-23(lf)* mutants (Table 1), and did not cause a P-Rvl phenotype or ectopic vulval fates at P8.p (*n = *166). However, the only other known extracellularly acting Wnt inhibitor in *C. elegans*, the transmembrane Wnt-binding Ror/CAM-1 tyrosine kinase, could mediate the effects of the CAN axons on Wnt signaling. Ror/CAM-1 is widely expressed in muscle, the vulval progenitors, and many neurons, including the CANs (Figure 6A) [7],[37],[44]–[46]. While in certain contexts, such as EGL-20/Wnt-mediated polarization of P7.p, it transduces Wnt signals [16], in other contexts such as inhibition of HSN migration, P3.p vulval progenitor cell specification, and the induction of vulval fates, it appears to antagonize Wnt signaling [7],[47]. Based on its ability to physically bind Wnts such as EGL-20 and CWN-1 (potential targets of *vab-8* mutations), and to interfere with vulval fate signaling in the P6.p epidermal progenitor when overexpressed in non-epidermal cells, it has been proposed that Ror/CAM-1 inhibits Wnt signaling by sequestering Wnts away from Wnt-responding cells [7]. While this model is plausible, how such a broadly expressed antagonist might refine Wnt gradients to allow specific migratory and tissue patterns is unclear.

To explore the possibility that Ror/CAM-1 might largely act from specific neurons such as the CANs to refine the EGL-20 and CWN-1 Wnt gradients that pattern the epidermis, we first examined the extent to which a *cam-1* null mutation, *gm122* (Figure 6C), phenocopies the effects of *vab-8* mutations. Similar to mutation of *vab-8*, *ror/cam-1* mutation elevated Wnt reporter activity in P6.px and P8.px progeny (Figure 6B). Also similar to mutation of *vab-8*, *ror/cam-1* mutation increased the frequency of P3.p becoming a vulval progenitor, cooperated with EGL-20/Wnt overexpression to cause embryonic P11 to P12 fate transformations, and suppressed the underinduced P6.p-based phenotype of an *egf/lin-3* mutation (Figure 6D; Table 2) [7]. Notably, mutation of *ror/cam-1* did not perturb primary vulval symmetry, induce ectopic vulval fates in P8.p (Figure 6E), or increase Wnt reporter activity to the same degree as *vab-8* mutations. These discrepancies may be due to dual negative and positive functions of Ror/CAM-1 in epidermal development. Within certain epidermal progenitors such as P7.p, Ror/CAM-1 may transduce Wnt signals, while in the CANs, it may sequester Wnts to refine the posteriorly derived Wnt gradients to which the vulval progenitors respond. Since Ror/CAM-1 mediates the polarizing effect of EGL-20/Wnt on P7.p [16], the P-Rvl phenotype, which stems from this polarization, cannot be manifested in *ror/cam-1* mutants. Furthermore, we found that Ror/CAM-1 is required for P8.p to adopt a vulval fate when the posterior CAN axons are foreshortened by *vab-8* mutations (Figure 6E). Further consistent with the model that Ror/CAM-1 positively transduces Wnt signals in P8.p, the increased frequency of Wnt reporter activity in P8.p progeny was significantly lower in *cam-1* mutants than in *vab-8* mutants (Figure 6E). The mildly elevated P8.px Wnt reporter activity in *ror/cam-1* mutants must therefore reflect activation of other Wnt receptors that are not sufficient to drive a vulval fate.

**Figure 7:**
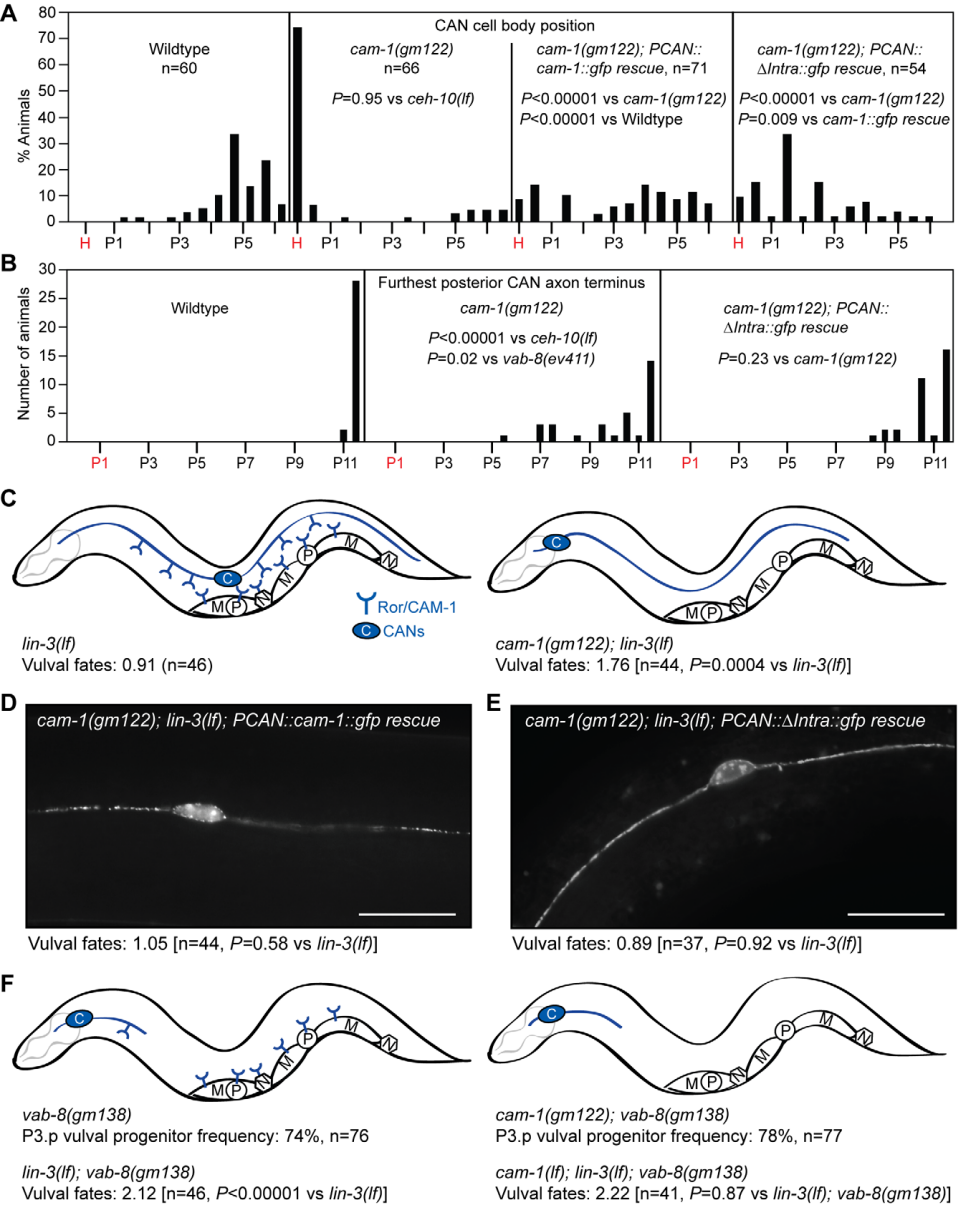
The CANs use the extracellular Wnt-binding domain of Ror/CAM-1 to direct epidermal patterning. (A and B) Distributions of positions of CAN cell bodies and furthest posterior CAN axon termini in *ror/cam-1* mutants and in *cam-1* mutants expressing wild-type or intracellular-domain-deleted CAM-1(ΔIntra) only in the CANs. In non-*ror/cam-1* rescue experiments, CANs were visualized with the *kyIs4[Pceh-23::gfp]* transgene. In *ror/cam-1* rescue experiments, CANs were visualized by expression of GFP-tagged Ror/CAM-1 in the CANs. In *ror/cam-1* rescue experiments, strains also harbored an *egf*/*lin-3(lf)* mutation. *x*-Axis indicates Pn.p or Pn.px positions. H, head/pharyngeal region. *p-*Values were calculated using a two-tailed Mann-Whitney *U* test. (C) Ror/CAM-1 inhibits vulval fate signaling in central vulval progenitors. (D) Transgenic CAN-specific expression of Ror/CAM-1::GFP restores inhibition of vulval development in *ror/cam-1* mutants. (E) Transgenic CAN-specific expression of a Ror/CAM-1::GFP mutant lacking the intracellular domain (ΔIntra, see Figure 6C) also restores inhibition of vulval development in *ror/cam-1* mutants. In (D) and (E), scale bar is 20 µm. (F) Under physiologic conditions, the majority of Ror/CAM-1 inhibition of vulval fate signaling is mediated by the CANs. If the CAN cell bodies are anteriorly displaced and the posterior axons are severely foreshortened, loss of Ror/CAM-1 activity from all cells does not further increase P3.p progenitor frequency or the amount of vulval development in sensitized backgrounds. Drawings depict the cellular distribution of Ror/CAM-1 as described in Figure 6E. M, muscle cells; N, neurons; P, vulval progenitors. In (C–E), vulval fates: number of vulval progenitor cells adopting vulval fates. Wild-type is 3.00. *p-*Values were calculated using a two-tailed Student's *t* test. The *PCAN::cam-1::gfp* and *PCAN::*Δ*Intra::gfp* rescuing transgenic arrays are *dyEx44* and *dyEx45*, respectively.

Since Ror/CAM-1 has been reported to affect CAN cell body positioning and axon outgrowth [35], we evaluated whether *cam-1* mutations might increase posteriorly derived Wnt signaling by affecting the location of the CANs and their axon termini. As has been previously reported, we found that *ror/cam-1* mutations caused anterior displacement of the CAN cell bodies that was as severe as that caused by *ceh-10* mutation [35] (Figures 5A and 7A). However, since *ceh-10* mutants do not have as strong Wnt phenotypes as *ror/cam-1* mutants, the CAN cell body displacement in *cam-1* mutants cannot explain their increased Wnt signaling. Also, in *ror/cam-1* mutants, outgrowth of the posterior CAN axon was only mildly affected, with the median end point being even more posterior than in *vab-8(ev411)* and *ceh-10(lf)* mutants that have no or weaker Wnt phenotypes (Figures 5B and 7B). Thus, Ror/CAM-1 does not inhibit Wnt signaling by regulating posterior CAN axon outgrowth, but could mediate an inhibitory effect of the extended axons.

To test whether Ror/CAM-1 expression in the CANs is sufficient to restore some aspect of normal inhibition of Wnt activity in epidermal progenitors, we transgenically expressed CAM-1 only in the CANs of *cam-1(null); egf/lin-3(lf)* double mutants, which have elevated Wnt signaling in the epidermal progenitors. Cell-specificity was confirmed by tagging the cDNA with *gfp*, which does not interfere with CAM-1 biological activity [47], and verifying that CAM-1::GFP expression was detected only in the CANs. In general, it was difficult to obtain transgenic lines with detectable and stable levels of CAM-1::GFP expression. However, one transgenic line with CAN-specific expression was selected for further analysis (Figure 7D). In this line, despite similar levels of expression between animals, re-expression of Ror/CAM-1 did not fully restore proper migration to the CANs (Figure 7A). This partial rescue suggests that in these animals, the level of transgenic Ror/CAM-1 expression may be just below physiologic amounts. However, of those animals displaying normal migration of at least one of the two CAN cell bodies, CAN-expressed Ror/CAM-1 completely restored the parental *egf/lin-3(lf)* underinduced phenotype (Figure 7C and 7D). This result indicates that epidermal progenitors such as P6.p no longer receive abnormally high Wnt signaling when Ror/CAM-1 is expressed only in the CANs of *cam-1(null)* mutants.

Similar to *vab-8* mutants, *cam-1* mutants also show an embryonic HSN overmigration phenotype, which has been proposed to be due to overactive EGL-20/Wnt signaling [23]. To evaluate whether Ror/CAM-1 could also be part of the embryonic mechanism by which the CANs regulate Wnt-dependent responses, we examined another transgenic line with CAN-specific expression of Ror/CAM-1::GFP. These animals had a *ror/cam-1* mutation and also expressed an HSN GFP marker to visualize HSN migration. Similar to the other transgenic line described above, this line also only weakly rescued the CAN migration defect (only ∼30% of animals had CANs in their normal mid-body position). However, despite this incomplete functional activity, these transgenic animals still showed significant, but not wild-type, rescue of HSN migration (Figure S10). These data further support a CAN-specific role for Ror/CAM-1 in cell non-autonomously regulating responses to Wnts.

To explore the model that Ror/CAM-1 acts from the CANs to sequester Wnts, we examined the requirement of the intracellular kinase domain for inhibition of Wnt signaling in the epidermis. Prior work showed that in conjunction with other Wnt receptor mutations, a *ror/cam-1* null mutation or a *cam-1* mutation affecting only the extracellular Wnt-binding domain (*sa692*, Figure 6C), but not a *cam-1* deletion affecting only the kinase domain (*ks52*, Figure 6C), can induce ectopic vulval fates [7]. To determine whether similar requirements for the extracellular, but not intracellular, kinase domain extend to the regulation of epidermal progenitor responses assayed in this work, we also examined the effects of these domain-specific mutations. Consistent with the prior work and our model, we found that the *cam-1(sa692)* mutation, but not the *cam-1(ks52)* mutation, increased the frequency of P3.p becoming a vulval progenitor and suppressed an *egf/lin-3* mutation comparably to the *cam-1* null mutation (Figure 6D; Table 2). To determine whether the extracellular-domain-only requirement specifically extended to CAN-based Ror/CAM-1 inhibition of epidermal progenitor responses to Wnts, we repeated our transgenic rescue experiments with a *cam-1* construct that lacked the entire intracellular domain (ΔIntra, Figure 6C). Similar to with the wild-type construct, it was difficult to generate transgenic lines with detectable GFP expression. Our best-expressing transgenic line showed partial rescue of the CAN cell body migration defect, but not as much as the wild-type-expressing line (Figure 7A), and there was no rescue of the CAN axon outgrowth or the mild tail-withering defects (Figures 7B and S11). Despite its limited activity in promoting proper CAN migration, the ΔIntra construct fully restored inhibition to vulval development in *ror/cam-1(gm122); egf/lin-3(lf)* mutants (Figure 7E). Also consistent with our model, it has been suggested that the ΔIntra construct may have a higher Wnt-binding capacity than the wild-type receptor [47], which could explain its seemingly more potent ability to restore Wnt inhibition.

To complement our transgenic add-back experiments with a more physiologic assessment of the Ror/CAM-1 site of action in regulating Wnt responses in epidermal progenitors, we used a genetic approach. We scored two quantitative epidermal phenotypes that are equally manifested in *ror/cam-1* and *vab-8* single mutants. In this strategy, we used a *vab-8* mutation to maximally displace the posterior CAN axon terminus away from the epidermal progenitors. If Ror/CAM-1 acts mostly through other neurons and muscle, a *cam-1* mutation should still significantly increase Wnt signaling in *vab-8* mutants. On the other hand, if Ror/CAM-1 acts mostly through the CANs, a *cam-1* mutation should have no further effect on Wnt signaling. Both *ror/cam-1* and *vab-8* single mutations have the same effect on the frequency with which P3.p becomes a vulval progenitor, increasing this frequency from 50% to ∼70% (Figures 2B and 6D). However, double mutants showed no further increase in this frequency (Figure 7F). Similarly, both *ror/cam-1* and *vab-8* mutations suppressed the vulvaless phenotype of *egf/lin-3* mutants centered at P6.p to a similar extent (Tables 1 and 2), yet combining the two mutations did not further increase this suppression (Figure 7F).

### Ror/CAM-1 Regulates EGL-20/Wnt Localization to the Posterior CAN Axon

Although Ror/CAM-1 is expressed at much higher levels in muscle than the CANs (Figure 6A), our data surprisingly indicate that CAN-expressed CAM-1 plays a critical role in regulating epidermal progenitor responses to Wnts such as EGL-20. While the EGL-20/Wnt gradient has been visualized in early L1 stage larvae [12], its potential refinement by specific cell populations like the CANs has not been reported. To investigate whether the EGL-20/Wnt gradient might be affected by the posterior CAN axons, we examined the distribution of EGL-20 in the vicinity of these axons. To accomplish this, we employed two transgenes. One expressed functional EGL-20/Wnt as a fusion to protein A, under the control of its native promoter (*Pegl-20::egl-20::protein A*) [12]. This transgene rescues *egl-20/wnt* mutant phenotypes, but does not cause gain-of-function phenotypes, and, therefore, expresses EGL-20 at near physiologic levels [12]. The second transgene expressed GFP under the control of the *ceh-23* promoter (*Pceh-23::gfp*), which drives expression in the CANs, as well as a few other neurons [48].

**Figure 8:**
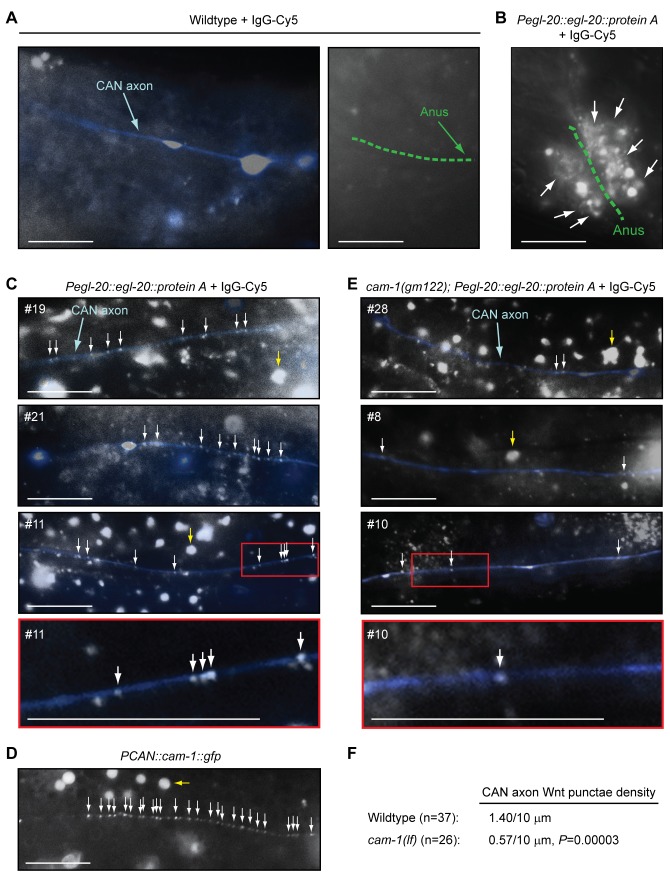
EGL-20/Wnt co-localizes with posterior CAN axons in vivo, in a Ror/CAM-1-dependent manner. CAN axons were visualized with the integrated *Pceh-23::gfp* transgene (*kyIs4*) (A–C and E), and EGL-20/Wnt was detected with the integrated *Pegl-20::egl-20::protein A* fusion transgene (*huIs60*) and injection of Cy5-conjugated rabbit IgG (IgG-Cy5) into living adult animals (B, C, and E). (A–C and E) The Cy5 channel is shown, as well as an overlay of the GFP channel, where the posterior CAN axon has been pseudo-colored blue. White arrows point to examples of EGL-20/Wnt punctae, and yellow arrows point to examples of variable background autofluorescence. In (C) and (E), numbers in the panels denote particular injected animals. Bottom panels are enlargements of red-boxed sections in the indicated animals. (D) Clustering of Ror/CAM-1 in the posterior CAN axon. Ror/CAM-1 was visualized as a functional translational GFP fusion from an extrachromosomal transgenic array (*PCAN::cam-1::gfp*; *dyEx44*) in animals that also carried an *egf/lin-3(lf)* mutation. Scale bars are 10 µm. (F) 37 independent measurements from 32 wild-type animals, and 26 independent measurements from 19 *ror/cam-1* mutants were used to calculate the mean EGL-20/Wnt density along the posterior CAN axon. *p-*Value was calculated using a two-tailed Mann-Whitney *U* test versus wild-type animals.

To visualize EGL-20/Wnt in living animals, we injected Cy5-labeled rabbit IgG, which has a high affinity for protein A, into double transgenic animals. This technique has been successfully used to label the extracellular portions of cell surface receptors, including protein-A-tagged receptors [49]. In single *Pceh-23::gfp* transgene control animals, IgG-Cy5 did not accumulate to a significant amount or label any cell surfaces (Figure 8A). In contrast, when injected into *Pegl-20::egl-20::protein A; Pceh-23::gfp* double transgenic animals, IgG-Cy5 labeled the surfaces of EGL-20/Wnt-producing rectal cells near the anus (Figure 8B), demonstrating specificity to the IgG-Cy5 signal. Cy5-labeled punctae were also enriched in other parts of the posterior body, but detection was variable, presumably because of variations in labeling between injected animals (e.g., Figure S12). However, the posterior enrichment of the Cy5-labeled punctae is consistent with the description of the EGL-20/Wnt gradient in fixed *Pegl-20::egl-20::protein A* L1 larvae stained with IgG-FITC [12]. Strikingly, Cy5 labeling was also detected along stretches of the posterior CAN axon, suggesting EGL-20/Wnt is bound to the axon (Figure 8C, three different animals are shown).

Since our data indicate that only the extracellular domain of Ror/CAM-1 is necessary to inhibit Wnt signaling from the CAN axons, and CAM-1 has been reported to bind Wnts such as EGL-20 in vitro [7], we tested whether CAM-1 mediates localization of EGL-20 to the posterior CAN axons. First, we observed that when driven by the CAN-specific promoter, Ror/CAM-1::GFP appeared as punctae along the CAN axon, as reported for other cells (Figure 8D) [50]–[52]. Notably, the Ror/CAM-1 punctae resembled the size and pattern of the EGL-20/Wnt punctae along the posterior CAN axon, suggesting EGL-20 might be bound to CAM-1. Next, we quantified the density of EGL-20/Wnt punctae along the posterior CAN axon in wild-type and *ror/cam-1* mutants. Mutation of *ror/cam-1* reduced the EGL-20/Wnt density along the axon, indicating that CAM-1 mediates some of the EGL-20 enrichment at the axon (Figure 8E and 8F). While in *ror/cam-1* mutants the measured EGL-20/Wnt density along the posterior CAN axon was not zero, it may have been lower than what we quantified because it is impossible to be absolutely certain what fraction of the punctae are actually bound to the axon. Nevertheless, these data indicate that EGL-20/Wnt is enriched at, or in very close proximity to, the posterior CAN axon in a Ror/CAM-1-dependent manner, and provide evidence for these axons affecting the distribution of EGL-20 in the extracellular space. These data are in accord with our functional analyses of the properties of the CAN axons, and provide a potential explanation for how the CAN axons exert their unique role in epidermal development.

## Discussion

Despite the identification of so many Wnt antagonists, our understanding of how Wnt gradients are established with precision and integrated with the activities of multiple growth factors is still quite limited. Here, we show that even to create an anatomically simple structure such as the *C. elegans* vulva, the diffusion of Wnts and their inhibitors does not provide sufficient spatial resolution of signaling. To overcome this problem, *C. elegans* has incorporated the unique axonal projections of specific neurons to set the exact strengths of Wnt signaling at precise locations. The CAN neurons use their posterior-directed axons and the Ror/CAM-1 Wnt receptor to regionally dampen the activity of posteriorly derived Wnt (EGL-20 and possibly CWN-1). This dampening prevents ectopic induction of vulval tissue in the posterior epidermis and ensures that other centrally produced Wnts specify proper symmetry in the primary vulva. However, this mechanism still permits the posterior-derived EGL-20 and CWN-1 Wnts to retain sufficient activity in the mid-body and anterior epidermis to specify the vulval progenitors, and cooperate with EGF/LIN-3 in promoting a 1° vulval fate in P6.p. Surprisingly, this type of regulation of epidermal pattering is unique to the Ror-expressing posterior CAN axons, and is not conferred by the other Ror/CAM-1-expressing cells along the anterior–posterior axis, or the SFRP-1-expressing cells. In fact, the posterior CAN axons are such potent regulators of Wnt signaling that foreshortening their normal length can dramatically alter an epidermal progenitor's response to Wnts. Given the unique role of the posterior CAN axons in regulating epidermal patterning, much of Ror/CAM-1 expression in other cells may be for more subtle control of epidermal patterning that we could not detect in our genetic experiments, or for inhibition of Wnt signaling for cell populations we did not assay, or could reflect other biological functions for CAM-1. For example, in muscle, Ror/CAM-1 is an important regulator of synapse strength [50]–[52], and in other cells, it plays important roles in cell polarity, cell migration, axon guidance, and neurite survival [16],[35],[37],[44]–[47],[53].

Although for technical reasons we could not quantify the EGL-20/Wnt gradient in the vicinity of the epidermal progenitors that respond to Wnt signaling, we could quantify other aspects of EGL-20 distribution in living adult animals. Notably, we detected EGL-20/Wnt punctae along the posterior CAN axon in a pattern similar to how Ror/CAM-1 clusters along the same axon, suggesting EGL-20 may bind to the axon via CAM-1. In support of this model, mutation of *ror/cam-1* reduced EGL-20/Wnt density along the posterior CAN axon. However, Ror/CAM-1 may not be the only factor determining where EGL-20 is distributed within the posterior body. Ror/CAM-1 is expressed at levels similar to the CANs in other neurons, and at much higher levels than the CANs in muscle (Figure 6). Yet, despite this expression pattern of Ror/CAM-1, EGL-20/Wnt does not appear to be proportionally concentrated at muscle (Figure S12), and appears to be somewhat enriched at the posterior CAN axon (Figure 8). Since Ror/CAM-1 has been reported to act in complexes with other Wnt-binding receptors [45],[46],[52], it is possible that part of the EGL-20-binding properties of the CAN axons arises from co-expression of other specific Wnt receptors or post-translational modifications of CAM-1 that affect its EGL-20-binding properties. Notably, in muscle, CAM-1 functions in a heteromeric complex with LIN-17/Frizzled to transduce CWN-2, but not EGL-20, Wnt signaling at the neuromuscular junction [52].

**Figure 9:**
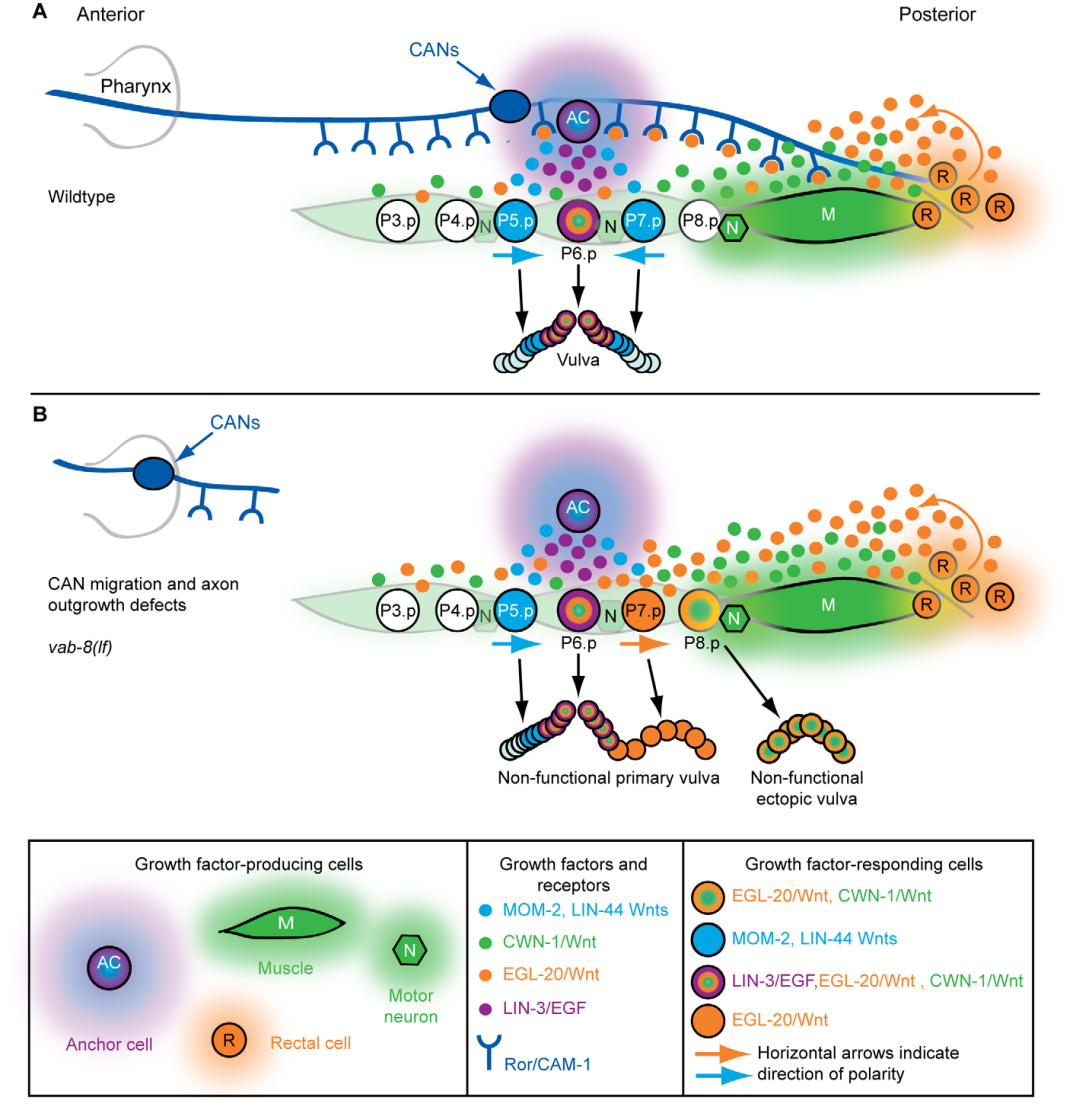
Model for neuronal control of axial patterning by Wnts. (A) In wild-type animals, EGL-20/Wnt (orange) (and possibly CWN-1/Wnt [green]) is sequestered by the Ror/CAM-expressing posterior CAN axons. At the end of the L2 larval stage, this sequestration allows MOM-2 and LIN-44 Wnts (blue) to reorient and polarize P7.p towards the anterior (horizontal arrows). During the L3 larval stage, anterior-facing P7.p divides with the mirror image pattern of P5.p, and P8.p does not receive sufficient Wnt or EGF signaling to adopt a vulval fate. (B) If the posterior CAN axons do not extend far enough into the posterior body, where EGL-20/Wnt is produced, an abnormally high concentration of EGL-20 occurs, which prevents anterior polarization of P7.p, and causes ectopic vulval tissue to form at P8.p.

Given the Ror/CAM-1-dependent localization of EGL-20/Wnt to the posterior CAN axon, the ability of CAM-1 to bind EGL-20 in vitro [7], and the requirement for only its Wnt-binding extracellular domain for CAM-1 to inhibit Wnt signaling from the CANs, it is possible that Wnt sequestration is part of the mechanism by which the CAN axons regulate epidermal patterning (Figure 9). Our data indicate that if Ror/CAM-1 is absent or if the posterior CAN axon does not grow to a sufficient length, the extracellular distribution of EGL-20/Wnt is altered. In these cases, in a simple model, the EGL-20/Wnt that is displaced from the CAN axons would be available to cause excess EGL-20 signaling in the epidermal progenitors, thereby perturbing normal Wnt patterning. Although only a small fraction of the total EGL-20/Wnt punctae appear to be bound to the posterior CAN axons, there are several possible scenarios in which this pool might be critical in determining the nature and strength of epidermal progenitor Wnt responses. First, many of the EGL-20/Wnt punctae that we detected at other locations might not be truly “free” and able to stimulate epidermal progenitors. They may be bound to other cells or trapped in the extracellular space. Thus, if the EGL-20/Wnt released from the posterior CAN axons is more diffusible than the other EGL-20, it may have a potent ability to change the pattern of epidermal progenitor responses to Wnts. Alternatively, the rectal cells may produce distinct forms of EGL-20/Wnt, with distinct abilities to bind and signal through different EGL-20 receptor complexes. Perhaps a form of EGL-20/Wnt that is specific for the epidermal progenitors is also preferentially sequestered by the CANs, endowing the CANs with a unique ability to regulate epidermal progenitor responses to EGL-20.

Although our data indicate a critical role during larval epidermal development for the posterior CAN axons in regulating Wnt responses, in certain settings, the CAN cell bodies may also be able to perform this function. Notably, EGL-20/Wnt-dependent HSN migration is completed prior to CAN axon outgrowth [54], yet embryonic laser ablation of the CAN precursor cells still causes HSN overmigration [35]. Similarly, genetic mutation of *ror/cam-1* also causes HSN overmigration [23], and we can partially rescue this phenotype by re-expressing Ror/CAM-1 specifically in the CANs (Figure S10). Together, these data suggest that Ror/CAM-1 might also be able to act from the CAN cell bodies to affect EGL-20/Wnt distributions, and hence the migration pattern of the HSNs.

Our data raise the possibility that besides their behavioral functions, another core property of neurons may be to act as unique spatial cues to refine developmental patterns created by growth factors such as Wnts. This refinement may be manifested at two levels. First, in a specific tissue in a given organism, a specific fixed pattern of neurons may increase the robustness of inducing a specific pattern. For example, in *C. elegans*, the CANs ensure that the normal epidermal pattern of a single vulva with mirror image symmetry in the mid-body is invariant. When the posterior CAN axons are severely foreshortened, additional epidermal patterns are observed. However, our data also indicate that the pattern of neuronal positioning and axon outgrowth can also affect the appearance of a tissue pattern. Shortening of the normal length of the posterior CAN axon causes a second vulva to inappropriately form in the posterior body and alters the normal symmetry of mid-body vulval tissue. Based on these data, we also suggest that at a broader level, more phenotypic diversity can be generated among tissues and organisms with neurons than without neurons. In a simplified model of aneural tissue patterning, Wnt gradients established by diffusion direct patterning, with distances between Wnt-producing and -responding cells mostly affecting the pattern. In contrast, in organisms with nervous systems, the layering of different intricate patterns of Wnt-sequestering neurons into developing tissues could expand the diversity of functional Wnt gradients, and hence the diversity of tissue patterns that are possible.

The ability of axons to refine Wnt signaling may be conserved, since Wnts are used extensively throughout metazoan development, and Ror kinases and other Wnt receptors are expressed in the nervous systems of diverse species [4],[5],[55],[56]. Furthermore, the close proximity of developing neural circuits and tissues is not unique to *C. elegans*. In vertebrates, neural crest cells begin to occupy the embryonic gut at the time of organ budding, and axon tracts are found in undifferentiated limb buds [57]–[59]. The ability of neurons to refine spatial domains of Wnt signaling may also be important after development for certain homeostatic functions. In mammals, Wnt signaling must be precisely regulated in specific niches to promote stem cell self-renewal and prevent tumorigenesis [60],[61]. By expressing the appropriate cognate receptors, neurons may even use the mechanism we have discovered to regulate the extracellular distributions of many other growth factors, thereby expanding the repertoire of biological processes under their control.

Although it was almost 200 years ago that Tweedy John Todd reported nerves were important for salamander limbs to regenerate after amputation [62], neurons have still not been widely demonstrated to be important for non-neuronal development. Most studies of non-behavioral effects of the nervous system have focused on the importance of whole nerves for muscle development and fracture healing [28],[63]–[67], and few mechanisms have been described for how the cellular components of nerves may exert non-behavioral effects. Notably, where examined, secretion of neurotransmitters or growth factors has always been part of the mechanism. In the salamander, Schwann cells from the sciatic nerve promote limb regeneration by releasing newt anterior gradient protein (nAG) [68]. In mammals, by secreting noradrenaline, muscarinic receptor agonists, and VEGF, nerves have been reported to modulate, respectively, hematopoietic stem cell migration, salivary gland cell proliferation, and endothelial cell differentiation into arteries [29],[69],[70]. By contrast, our work provides evidence for a non-secretory function of neurons, where, by binding of one of the oldest conserved metazoan growth factors, neurons can help organize the complex signaling patterns that direct tissue development.

The use of neurons to refine patterning by Wnts may have arisen from the ancestral functions of Wnts in establishing and patterning the primary body axis, and their subsequent use in directing neuronal migration, axon guidance, and synaptic strength [4],[56]. By additionally sequestering Wnts, neurons provide an efficient and unique mechanism to reshape initial Wnt gradients and help generate distinct body patterns. Given that the neuronal function we describe here does not involve the release of canonical neurotransmitters, but rather involves the axon outgrowth properties of neurons, our data raise intriguing questions regarding the earliest functional properties of primordial neurons. In addition to their behavioral importance, the evolution of neurons may also have improved metazoans by refining the plans of the bodies they would ultimately control.

## Materials and Methods

### Strains

*C. elegans* were cultured at 20°C [71]. Loss-of-function alleles included the following: LGII: *cwn-1(ok546)*, *let-23(sy1)*, *unc-4(e120)*, *cam-1(gm122)*, *cam-1(sa692)*, and *cam-1(ks52)*; LGIII: *ceh-10(gm120)* and *pha-1(e2123ts)*; LGIV: *lin-3(n378)* and *egl-20(n585)*; LGV: *vab-8(gm99)*, *vab-8(gm138)*, *vab-8(ev411)*, and *him-5(e1490)*. Integrated transgenes included *muIs49*, *syIs187*, *kyIs4*, *huIs60*, and *cwIs6* (contains a full-length rescuing genomic *Pcam-1::cam-1::gfp* construct; a gift from Wayne Forrester). The deletion allele *cwn-1(ok546)* was generated by the *C. elegans* Gene Knockout Facility at the Oklahoma Medical Research Foundation. See Text S1 for references.

### Vulval Induction and P3.p Fate

Vulval development was scored during the L4 stage under DIC optics using a Zeiss Axio Imager. Animals were anesthetized with 10 mM sodium azide unless otherwise indicated. The number of vulval nuclei was used to extrapolate how many vulval progenitor cells adopted vulval fates. A vulval progenitor cell generating seven or eight great granddaughters (Pn.pxxx) and no hyp7 tissue was scored as 1.0 cell induction. A vulval progenitor cell in which one daughter (Pn.px) fuses with hyp7, and the other daughter generates three or four great granddaughters was scored as 0.5 cell induction. In wild-type animals, P5.p, P6.p, and P7.p each undergo 1.0 cell induction, resulting in a total of 3.0 cell induction. Animals with more than 3.0 cell induction are overinduced, and animals with less than 3.0 cell induction are underinduced. To determine whether P3.p had become a vulval progenitor, all Pn.p (P1.p–P11.p) progeny nuclei were counted at the mid-L4 stage and used to extrapolate whether P3.p had divided. P3.p division is indicative of having become a vulval progenitor.

### Transgenics

Transgenic extrachromosomal arrays were generated as described [72]. See Text S1 for details of plasmid constructions and descriptions of transgenic arrays.

### Wnt Reporter Scoring

Wnt activity was visualized using the integrated POPTOP mCherry reporter *syIs187*. Larvae were anesthetized with 5 mM levamisole and photographed using epifluorescence and DIC on an Axio Imager Z1 microscope using AxioVision software (Zeiss). In the mCherry channel, animals were photographed with an exposure time of 1,200 ms. To set a threshold for reporter detection, fluorescent image files were adjusted to a brightness of −0.59 and a contrast setting of 1.11. For reporter scoring in P5.px–P7.px progeny, images were adjusted to a brightness of −0.50 and contrast of 1.22.

### RNA Interference

HT115 *Escherichia coli* bacteria containing RNAi clones (L4440, empty RNAi vector; K100B4.6, *cwn-1*; F38E1.7, *mom-2*) were obtained from the Ahringer Lab bacterial feeding library [73], and RNAi was performed as described [74].

### Fluorescence Microscopy and EGL-20/Wnt::protein A Detection

Some images were captured on a Nikon Eclipse T1 confocal microscope, with worms being anesthetized in 500 mM sodium azide (Figures 3A–3C, 4E–4H, 5C, and 6A). Other images were taken with a Zeiss Axio Imager Z1, with worms being anesthetized with 5 mM levamisole (Figures 4A, 4C, 4D, 5E, 7D, 7E, and 8). CAN positions were scored on a Zeiss Axio Imager Z1, with worms being anesthetized in 0.1% tricaine/1.7 mM levamisole or 5 mM levamisole. To label EGL-20/Wnt::protein A in vivo, adult animals were injected with 10 µg/ml Cy5-rabbit anti-rat IgG (Jackson) diluted in 20 mM K_3_PO_4_, 3 mM potassium citrate, 2% PEG_6000_ (pH 7.4). Animals were scored 6–10 h after injection.

## Supporting Information

Figure S1 **Mispositioning of the CAN cell bodies and foreshortening of the posterior axons do not perturb excretory cell morphology.** (A–C) Photographs of L4 animals carrying the excretory cell *bgIs312[Ppes-6::gfp]* reporter transgene. Scale bar is 100 µm. (B) Arrows point to cysts that have abnormally formed in *exc-5(lf)* mutants. In some animals, the excretory canals have truncated prematurely.
(PDF)

Figure S2 **Mutation of** ***axin/pry-1*** **strongly activates a Wnt-responsive reporter in epidermal progenitors and promotes vulval fates.** (A) Representative L3, Pn.px stage animals harboring the integrated *syIs188* Wnt reporter transgene. This integrated transgene is derived from the same extrachromosomal array found in the *syIs187* integrant. Animals were photographed with the same exposure time (1,000 ms). Asterisks denote non-epidermal progenitor cells showing Wnt-independent reporter expression. Scale bar is 10 µm. (B) Quantification of the number of animals at the Pn.px stage with detectable *syIs188* Wnt reporter activity. *p-*Values were calculated using a two-tailed Fisher's exact test. (C) Quantification of average pixel density per positive Pn.px cell. For control wild-type animals, P5.px and P6.px cells not scoring positive by visual inspection had to be used to obtain a minimum of two cells to determine mean pixel intensity. *p-*Values were calculated using a two-tailed Student's *t* test. (D) *syIs188* Wnt reporter expression in P9.p and P10.p progeny at the Pn.px stage. Upper panel, 1,000 ms exposure. Lower panel, 250 ms exposure. Scale bar is 10 µm. (E) Frequency of ectopic induction of vulval fates in *axin/pry-1* mutants. *p-*Values were calculated using a two-tailed Fisher's exact test.
(PDF)

Figure S3 ***cwn-1*** **is expressed in the CANs.** Image of a hermaphrodite expressing the *Pcwn-1::yfp* reporter in muscle and one of the pair of CAN cell bodies from the *dyEx34* extrachromosomal transgenic array. Scale bar is 10 µm.
(PDF)

Figure S4 ***vab-8*** **mutations increase Wnt-dependent HSN migration and cause P11 to P12 fate transformations.** (A) HSNs are born in the posterior of the embryo close to the EGL-20/Wnt-producing cells, and are directed anteriorly by EGL-20 to the prospective vulval region. (B–E) HSNs were visualized with the *zdIs13[Ptph-1::gfp]* transgene. The integrated *muIs49* transgene (*Pegl-20::egl-20::gfp*) introduces extra functional copies of the genomic *egl-20/wnt* locus fused to *gfp* into the genome. (F) Schematic of P11 and P12 lineages showing the unique dependence of the P12 fate on Wnt signaling. In wild-type animals, P11 does not respond to Wnt signaling and generates a large cell (P11.p) that fuses with hyp7. By contrast, P12 normally responds to LIN-3/EGF and LIN-44/Wnt to generate a smaller cell (P12.pa) that fuses with hyp12. (G) The CAN neurons help minimize ectopic induction of the P12 fate in P11. *p-*Values were calculated using a two-tailed Fisher's exact test.
(PDF)

Figure S5 **The increased proximity of epidermal progenitors to posterior Wnt sources in** ***vab-8*** **mutants is not sufficient to promote vulval fate signaling.** (A) Correlation of median CAN positions (cell bodies and posterior axon termini) with distance of posterior P8.p progeny from the anus (the approximate location of the EGL-20/Wnt-producing cells) in L3, Pn.px stage animals (CAN data are from Figure 5A and 5B). Animals were anesthetized with 0.1% tricaine/1.7 mM levamisole. *p-*Values were calculated using a two-tailed Student's *t* test. (B) Distances of P6.pp and P8.pp, respectively, from the anus in L3, Pn.px stage animals. Animals harbored the *syIs187* integrated Wnt reporter and were anesthetized with 5 mM levamisole. The *p-*Value was calculated using a two-tailed Student's *t* test. (C) Distributions of positions of CAN cell bodies and furthest posterior CAN axon termini in L3, Pn.px stage *dpy-17(lf); dpy-20(lf)* mutants. (D) Representative *dpy-17(lf); dpy-20(lf)* double mutant at the L3, Pn.px stage showing the normal position of the posterior CAN axon terminus, which extends just past P11.p. Scale bar is 20 µm. In (A), (C), and (D) animals harbored the *kyIs4[Pceh-23::gfp]* transgene to mark the CANs. (E) Frequency with which P3.p becomes a vulval progenitor. (F) Frequency with which P8.p adopts a vulval fate. In (E) and (F) *p-*Values were calculated using a two-tailed Fisher's exact test.
(PDF)

Figure S6 **The reduced posterior body volume in** ***vab-8*** **mutants is not sufficient to promote vulval fate signaling in epidermal progenitors.** (A–D) Images showing posterior surface areas for posterior body volume calculations in wild-type, *vab-8(lf)*, and *dpy-17(lf); dpy-20(lf)* mutants. Note that some *vab-8* mutants with ectopic vulval fates at P8.p had posterior surface areas similar to those of some wild-type animals (compare [A] and [B]). (E) Table of posterior body volumes for wild-type, *vab-8(lf)*, and *dpy-17(lf); dpy-20(lf)* mutants. Volumes were calculated by multiplying the mean thickness by the measured posterior surface area for each animal. *p-*Values were calculated using a two-tailed Student's *t* test. Note that the mean posterior body volumes of the total *vab-8(lf)* population, and of the *vab-8* mutants specifically having ectopic vulval fates at P8.p, were not less than those of control *dpy-17(lf); dpy-20(lf)* mutants. (F and G) Body morphology of *egfr/let-23(lf)* mutant L1 larvae within minutes of being placed on 3% agar pads with either 50 mM (F) or 400 mM (G) sodium chloride. (H) Table of whole body volumes for *egfr/let-23(lf)* mutants as described in (E). The *vab-8(lf)* allele is *gm138*. Scale bar is 100 µm.
(PDF)

Figure S7 **Mispositioning of the CAN cell bodies and foreshortening of the posterior axons do not increase** ***egl-20/wnt*** **mRNA levels.** *egl-20/wnt* mRNA transcripts were measured in pooled L1 worms by quantitative real-time PCR. Expression is plotted relative to that in wild-type animals. *muIs49* animals contain a functional, rescuing integrated *Pegl-20::egl-20::gfp* transgenic array.
(PDF)

Figure S8 **In** ***ceh-10*** **mutants, ectopic vulval induction at P8.p correlates with foreshortening of the posterior CAN axon, but not with tail withering.** Positions of uninduced P8.px cells or ectopic vulvae from induced P8.px cells relative to the anus are schematized. CAN axons were visualized with the *kyIs4[Pceh-23::gfp]* transgene in animals that were anesthetized with 0.1% tricaine/1.7 mM levamisole. Distances were measured from the middle of the uninduced P8.px cells or the middle of the ectopic vulva to the anus in L4 stage animals. M, muscle; N, neuron; R, rectal cells. Intensity of the green color is proportional to the amount of CWN-1/Wnt produced.
(PDF)

Figure S9 **CAN cell body proximity to P3.p is not correlated with P3.p becoming a vulval progenitor.** (A) *cwn-1/wnt* and *egl-20/wnt* activity are necessary for the increased frequency of P3.p becoming a vulval progenitor in *vab-8* mutants. *p-*Values were calculated using a two-tailed Fisher's exact test. (B) Table of the positions of the two CAN cell bodies and their posterior axon termini in individual *ceh-10* mutants, and whether P3.p became a vulval progenitor in these animals. The table includes only animals where at least one of the cell bodies among the pair of CANs was displaced closer to P3.p, away from the normal median position of P5.p. *p-*Value was calculated using a two-tailed Fisher's exact test. (C) Distributions of the CAN cell bodies closest and furthest to P3.p (among the pair) in animals where at least one cell body was close to P3.p, and relationship to P3.p becoming a vulval progenitor. H, head/pharyngeal region. *p-*Values were calculated using a two-tailed Mann-Whitney *U* test.
(PDF)

Figure S10 **Transgenic CAN-specific expression of Ror/CAM-1 in** ***cam-1(null)*** **mutants partially rescues the embryonic HSN overmigration phenotype.** The rescuing array is *akEx1601*. HSNs were visualized with the *zdIs13[Ptph-1::gfp]* transgene. *p-*Values were calculated using a two-tailed Mann-Whitney *U* test.
(PDF)

Figure S11 **Tail withering and reduced posterior body volumes do not account for the effects of** ***ror/cam-1*** **mutations on Wnt signaling.** (A–C) Images showing posterior surface areas for posterior body volume calculations in wild-type animals and *ror/cam-1* mutants. Scale bar is 100 µm. Note that restoration of Ror/CAM-1 specifically to the CANs can restore inhibition of vulval development without rescuing tail withering (compare [B] and [C]). (D) Table of posterior body volumes for wild-type, *ror/cam-1* mutant, and CAM-1-rescued animals. Volumes were calculated by multiplying the measured thickness and surface area for each animal. *p-*Values were calculated using a two-tailed Student's *t* test. Note that the ΔIntra *ror/cam-1* construct that lacks the entire intracellular domain, but retains the Wnt-binding extracellular domain, does not rescue the withered tail phenotype of *cam-1* mutants. Also note that *ror/cam-1* mutants with increased signaling in vulval progenitors (mean 2.68 progenitors adopting vulval fates) do not have significantly greater tail withering than mutants with no increased signaling (mean 0.70 progenitors adopting vulval fates).
(PDF)

Figure S12 **EGL-20/Wnt distribution in the posterior body of adult animals.** EGL-20/Wnt was detected with the integrated *Pegl-20::egl-20::protein A* fusion transgene (*huIs60*) and injection of Cy5-conjugated rabbit IgG (IgG-Cy5) into living adult animals (B and C). White arrows point to examples of EGL-20/Wnt punctae, and yellow arrows indicate examples of variable background autofluorescence. Scale bars are 20 µm. Numbers in the panels denote particular injected animals.
(PDF)

Table S1 **The CAN neurons inhibit vulval fate signaling.**
(DOC)

Table S2 ***vab-8*** **mutants have a mild delay in larval growth that is not as pronounced as severe osmotic stress.**
(DOC)

Table S3 **Osmotic stress and a shortened body length do not promote vulval fate signaling.**
(DOC)

Table S4 **Mutants with reduced vulval development that maintain wild-type Wnt pathway activity do not have increased sensitivity to Wnt gene activity.**
(DOC)

Text S1 **Supplementary results, supplementary materials and methods, and supplementary references.**
(DOC)

## Abbreviations

CAN: canal-associated neuron
HSN: hermaphrodite-specific neuron
P-Rvl: posterior-reversed vulval lineage
RNAi: RNA interference
