# Supplementary figures and images for: Neurons Refine the Caenorhabditis elegans Body Plan by Directing Axial Patterning by Wnts
Source: PLoS Biol. 2013 Jan 8;11(1):e1001465. doi: 10.1371/journal.pbio.1001465 (PMC3539944; doi:10.1371/journal.pbio.1001465)

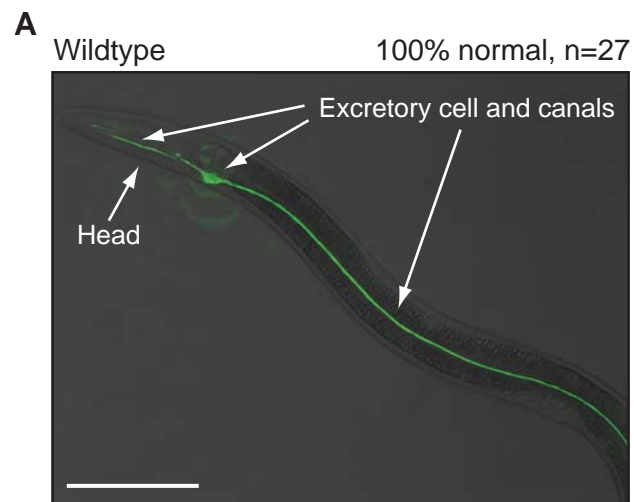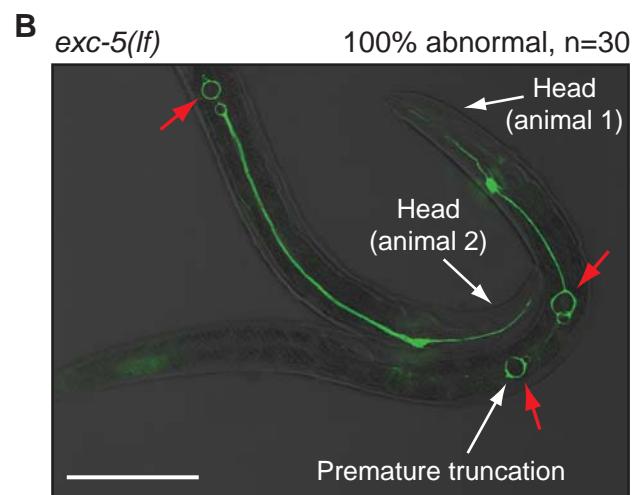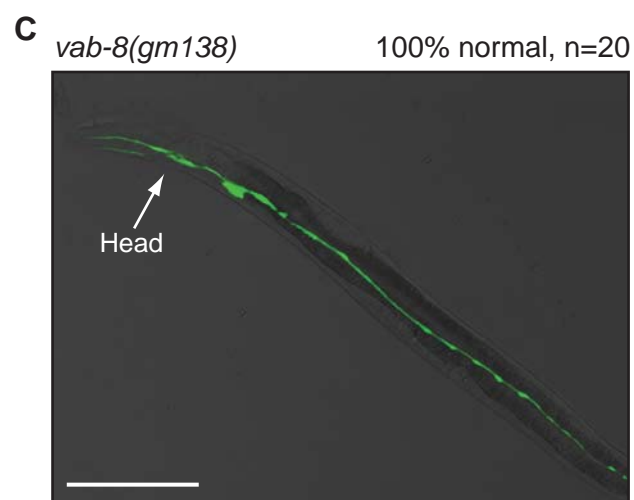

Supplement: Figure S1 — Mispositioning of the CAN cell bodies and foreshortening of the posterior axons do not perturb excretory cell morphology. (A–C) Photographs of L4 animals carrying the excretory cell bgIs312[Ppes-6::gfp] reporter transgene. Scale bar is 100 µm. (B) Arrows point to cysts that have abnormally formed in exc-5(lf) mutants. In some animals, the excretory canals have truncated prematurely. (PDF) [file pbio.1001465.s001.pdf]

**A**

Wnt reporter, wildtype

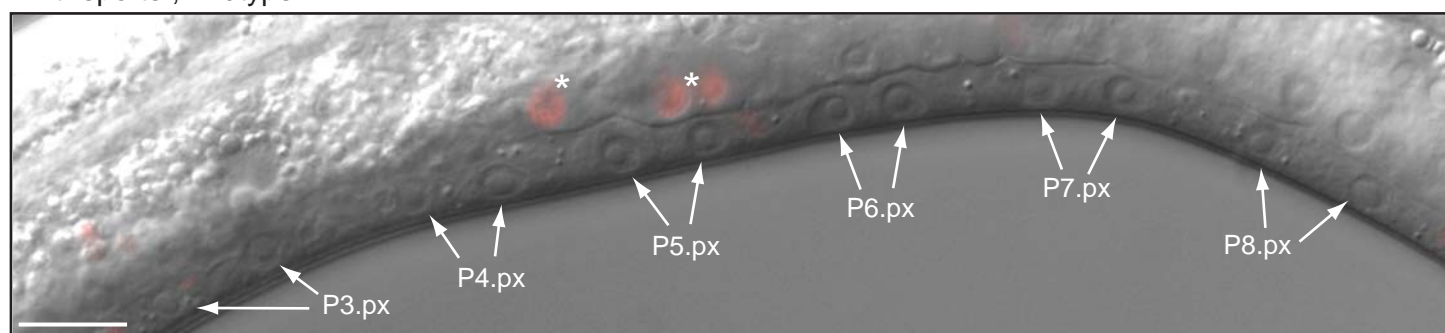Wnt reporter, *pry-1(lf)*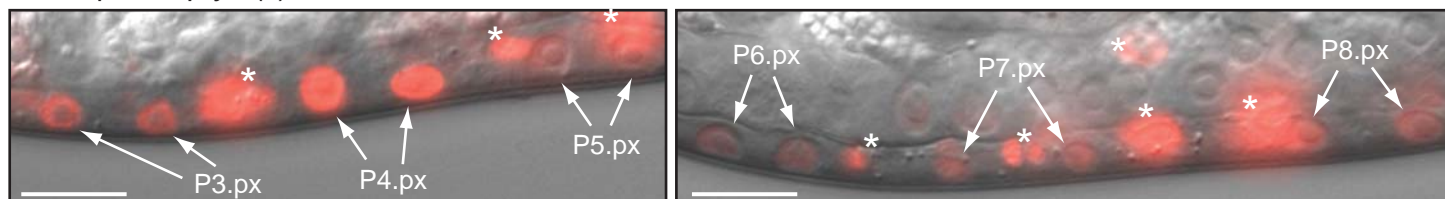**B**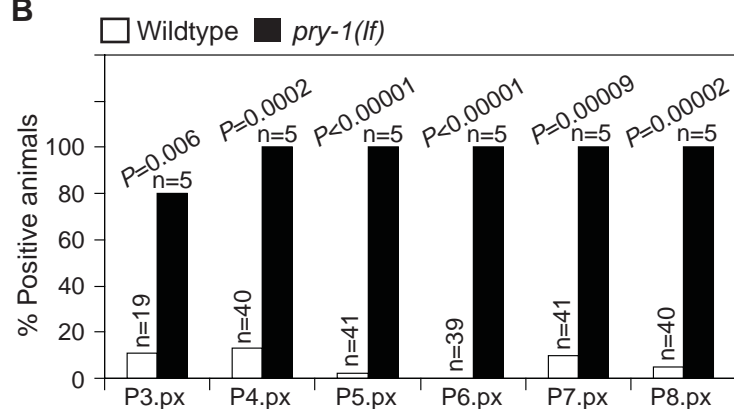**C**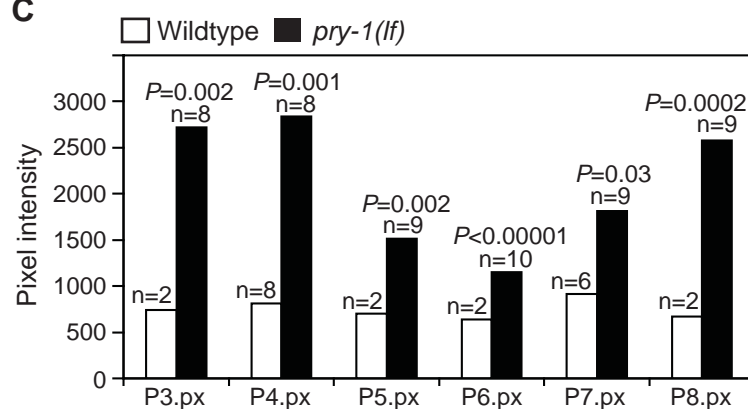**D**Wnt reporter, *pry-1(lf)*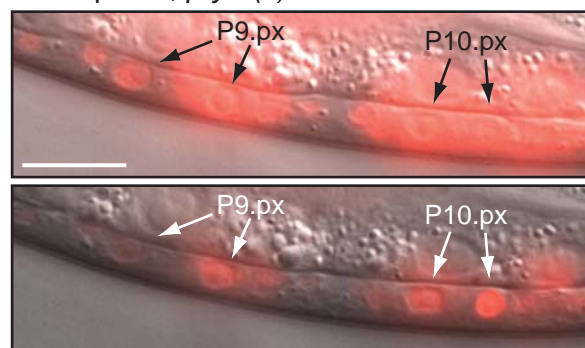**E**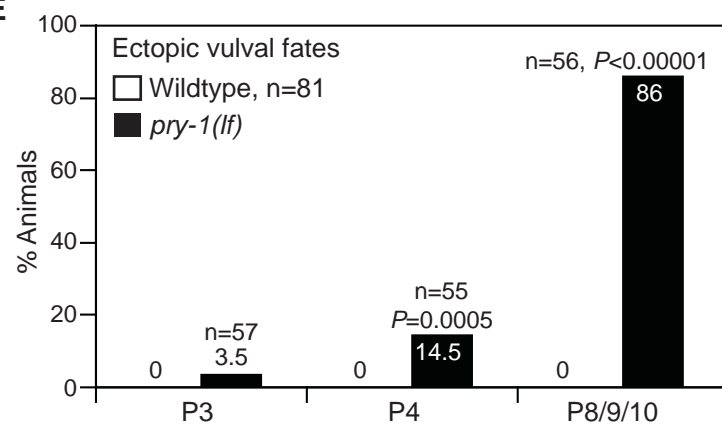

Supplement: Figure S2 — Mutation of axin/pry-1 strongly activates a Wnt-responsive reporter in epidermal progenitors and promotes vulval fates. (A) Representative L3, Pn.px stage animals harboring the integrated syIs188 Wnt reporter transgene. This integrated transgene is derived from the same extrachromosomal array found in the syIs187 integrant. Animals were photographed with the same exposure time (1,000 ms). Asterisks denote non-epidermal progenitor cells showing Wnt-independent reporter expression. Scale bar is 10 µm. (B) Quantification of the number of animals at the Pn.px stage with detectable syIs188 Wnt reporter activity. p-Values were calculated using a two-tailed Fisher's exact test. (C) Quantification of average pixel density per positive Pn.px cell. For control wild-type animals, P5.px and P6.px cells not scoring positive by visual inspection had to be used to obtain a minimum of two cells to determine mean pixel intensity. p-Values were calculated using a two-tailed Student's t test. (D) syIs188 Wnt reporter expression in P9.p and P10.p progeny at the Pn.px stage. Upper panel, 1,000 ms exposure. Lower panel, 250 ms exposure. Scale bar is 10 µm. (E) Frequency of ectopic induction of vulval fates in axin/pry-1 mutants. p-Values were calculated using a two-tailed Fisher's exact test. (PDF) [file pbio.1001465.s002.pdf]

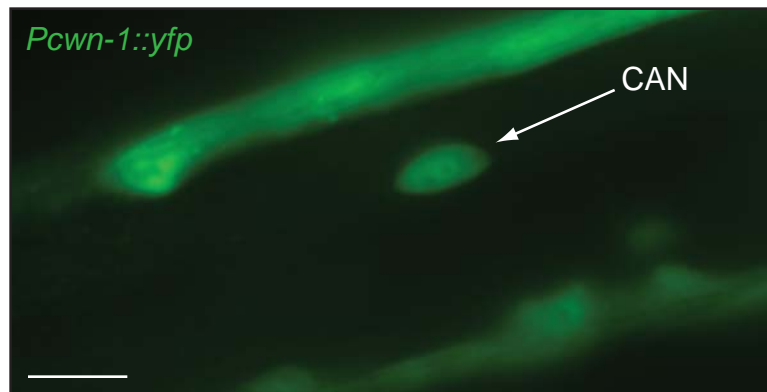

Supplement: Figure S3 — cwn-1 is expressed in the CANs. Image of a hermaphrodite expressing the Pcwn-1::yfp reporter in muscle and one of the pair of CAN cell bodies from the dyEx34 extrachromosomal transgenic array. Scale bar is 10 µm. (PDF) [file pbio.1001465.s003.pdf]

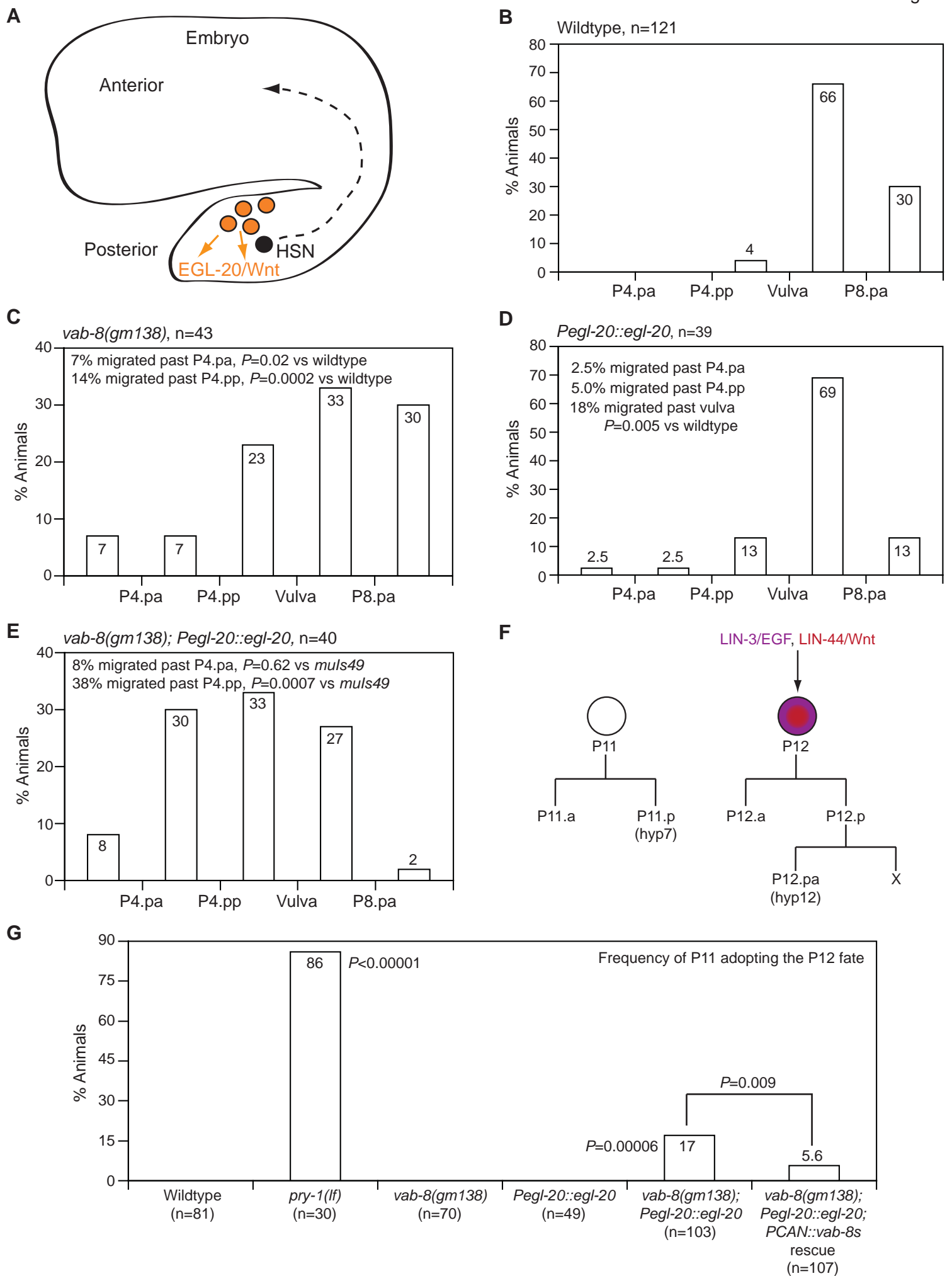

Supplement: Figure S4 — vab-8 mutations increase Wnt-dependent HSN migration and cause P11 to P12 fate transformations. (A) HSNs are born in the posterior of the embryo close to the EGL-20/Wnt-producing cells, and are directed anteriorly by EGL-20 to the prospective vulval region. (B–E) HSNs were visualized with the zdIs13[Ptph-1::gfp] transgene. The integrated muIs49 transgene (Pegl-20::egl-20::gfp) introduces extra functional copies of the genomic egl-20/wnt locus fused to gfp into the genome. (F) Schematic of P11 and P12 lineages showing the unique dependence of the P12 fate on Wnt signaling. In wild-type animals, P11 does not respond to Wnt signaling and generates a large cell (P11.p) that fuses with hyp7. By contrast, P12 normally responds to LIN-3/EGF and LIN-44/Wnt to generate a smaller cell (P12.pa) that fuses with hyp12. (G) The CAN neurons help minimize ectopic induction of the P12 fate in P11. p-Values were calculated using a two-tailed Fisher's exact test. (PDF) [file pbio.1001465.s004.pdf]

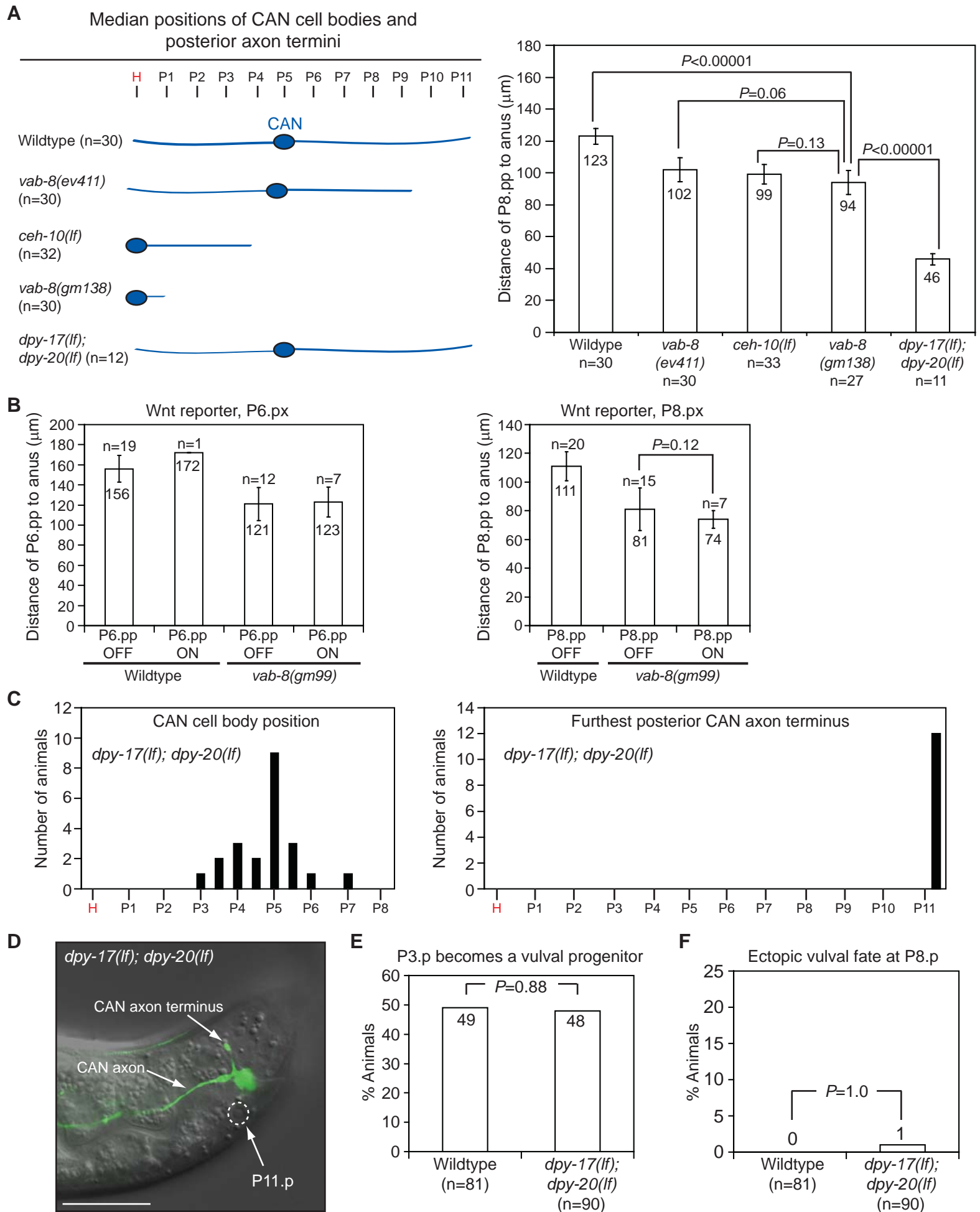

Supplement: Figure S5 — The increased proximity of epidermal progenitors to posterior Wnt sources in vab-8 mutants is not sufficient to promote vulval fate signaling. (A) Correlation of median CAN positions (cell bodies and posterior axon termini) with distance of posterior P8.p progeny from the anus (the approximate location of the EGL-20/Wnt-producing cells) in L3, Pn.px stage animals (CAN data are from Figure 5A and 5B). Animals were anesthetized with 0.1% tricaine/1.7 mM levamisole. p-Values were calculated using a two-tailed Student's t test. (B) Distances of P6.pp and P8.pp, respectively, from the anus in L3, Pn.px stage animals. Animals harbored the syIs187 integrated Wnt reporter and were anesthetized with 5 mM levamisole. The p-Value was calculated using a two-tailed Student's t test. (C) Distributions of positions of CAN cell bodies and furthest posterior CAN axon termini in L3, Pn.px stage dpy-17(lf); dpy-20(lf) mutants. (D) Representative dpy-17(lf); dpy-20(lf) double mutant at the L3, Pn.px stage showing the normal position of the posterior CAN axon terminus, which extends just past P11.p. Scale bar is 20 µm. In (A), (C), and (D) animals harbored the kyIs4[Pceh-23::gfp] transgene to mark the CANs. (E) Frequency with which P3.p becomes a vulval progenitor. (F) Frequency with which P8.p adopts a vulval fate. In (E) and (F) p-Values were calculated using a two-tailed Fisher's exact test. (PDF) [file pbio.1001465.s005.pdf]

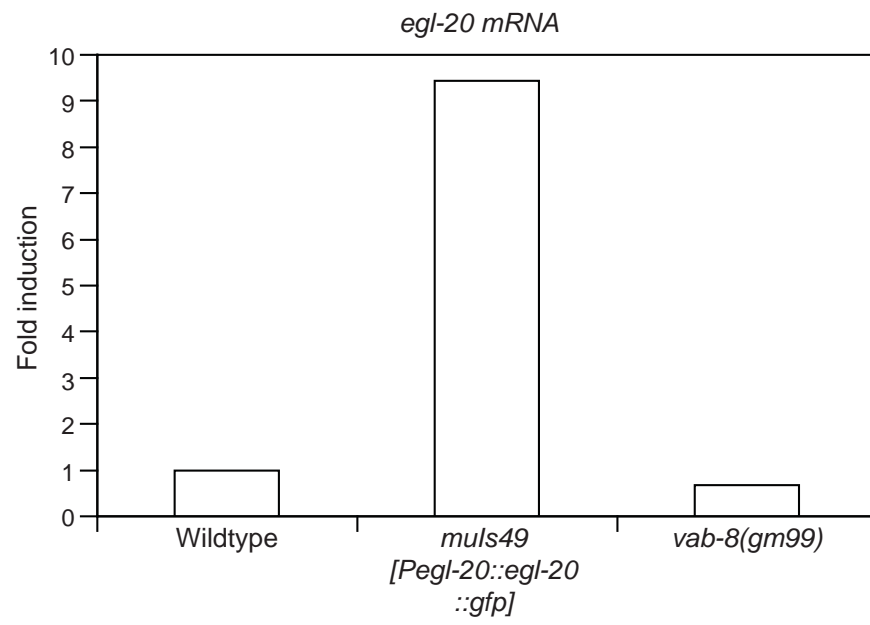

Supplement: Figure S7 — Mispositioning of the CAN cell bodies and foreshortening of the posterior axons do not increase egl-20/wnt mRNA levels. egl-20/wnt mRNA transcripts were measured in pooled L1 worms by quantitative real-time PCR. Expression is plotted relative to that in wild-type animals. muIs49 animals contain a functional, rescuing integrated Pegl-20::egl-20::gfp transgenic array. (PDF) [file pbio.1001465.s007.pdf]

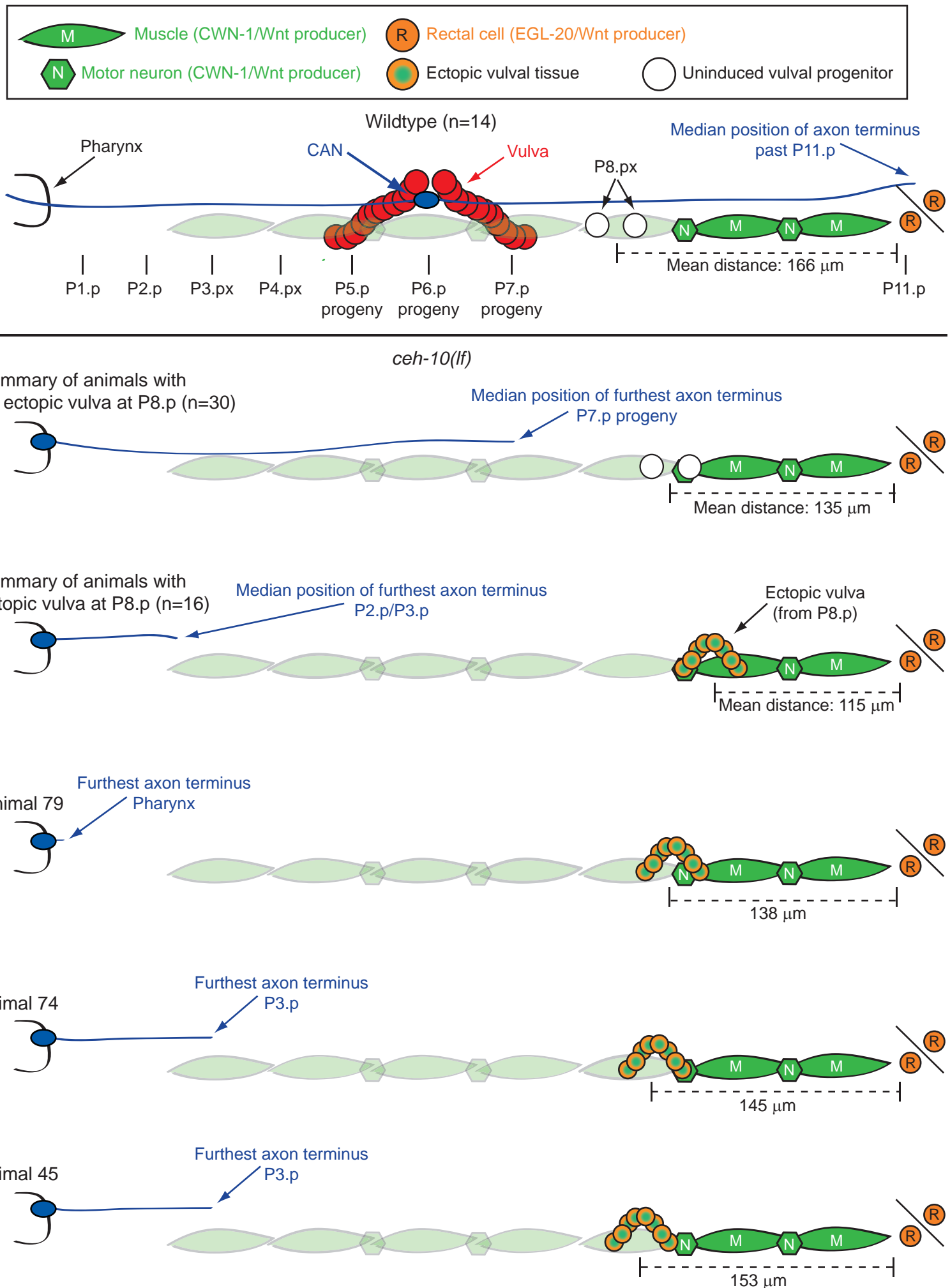

Supplement: Figure S8 — In ceh-10 mutants, ectopic vulval induction at P8.p correlates with foreshortening of the posterior CAN axon, but not with tail withering. Positions of uninduced P8.px cells or ectopic vulvae from induced P8.px cells relative to the anus are schematized. CAN axons were visualized with the kyIs4[Pceh-23::gfp] transgene in animals that were anesthetized with 0.1% tricaine/1.7 mM levamisole. Distances were measured from the middle of the uninduced P8.px cells or the middle of the ectopic vulva to the anus in L4 stage animals. M, muscle; N, neuron; R, rectal cells. Intensity of the green color is proportional to the amount of CWN-1/Wnt produced. (PDF) [file pbio.1001465.s008.pdf]

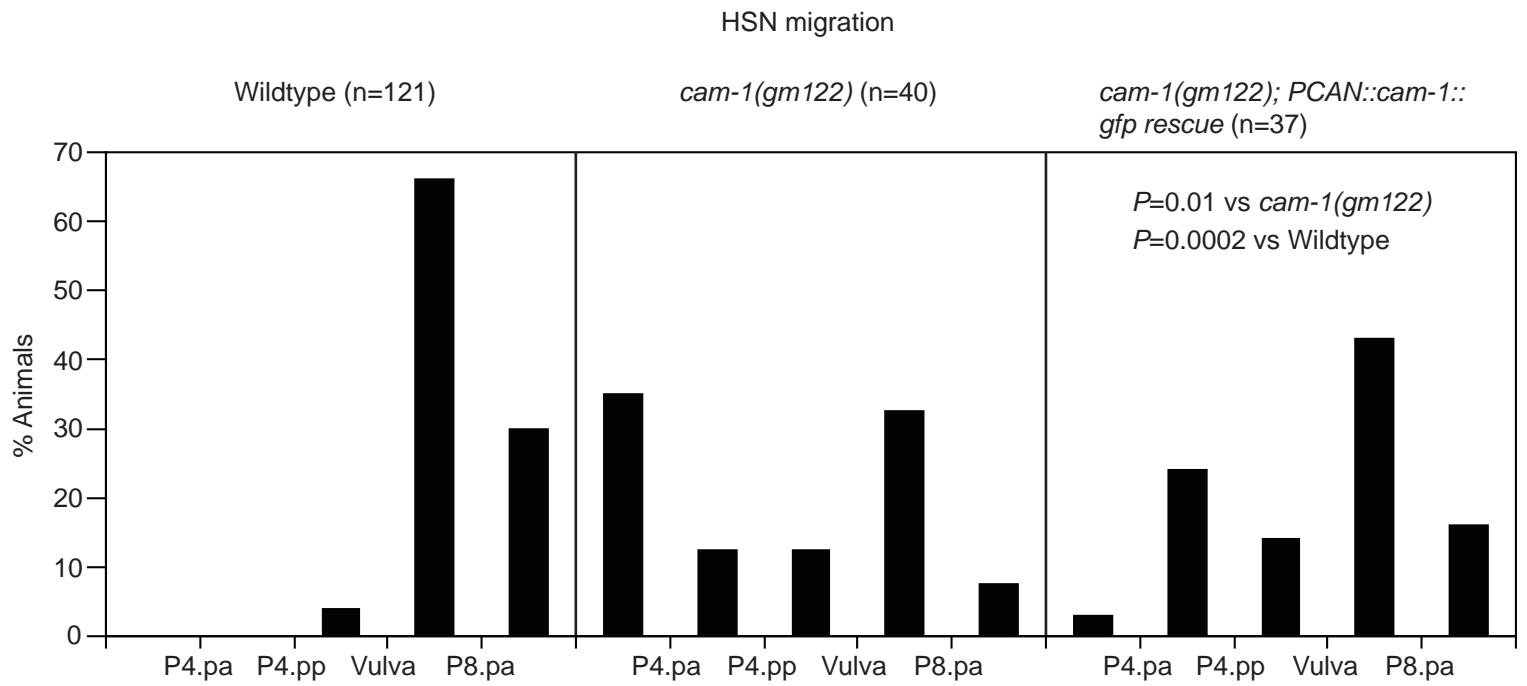

Supplement: Figure S10 — Transgenic CAN-specific expression of Ror/CAM-1 in cam-1(null) mutants partially rescues the embryonic HSN overmigration phenotype. The rescuing array is akEx1601. HSNs were visualized with the zdIs13[Ptph-1::gfp] transgene. p-Values were calculated using a two-tailed Mann-Whitney U test. (PDF) [file pbio.1001465.s010.pdf]

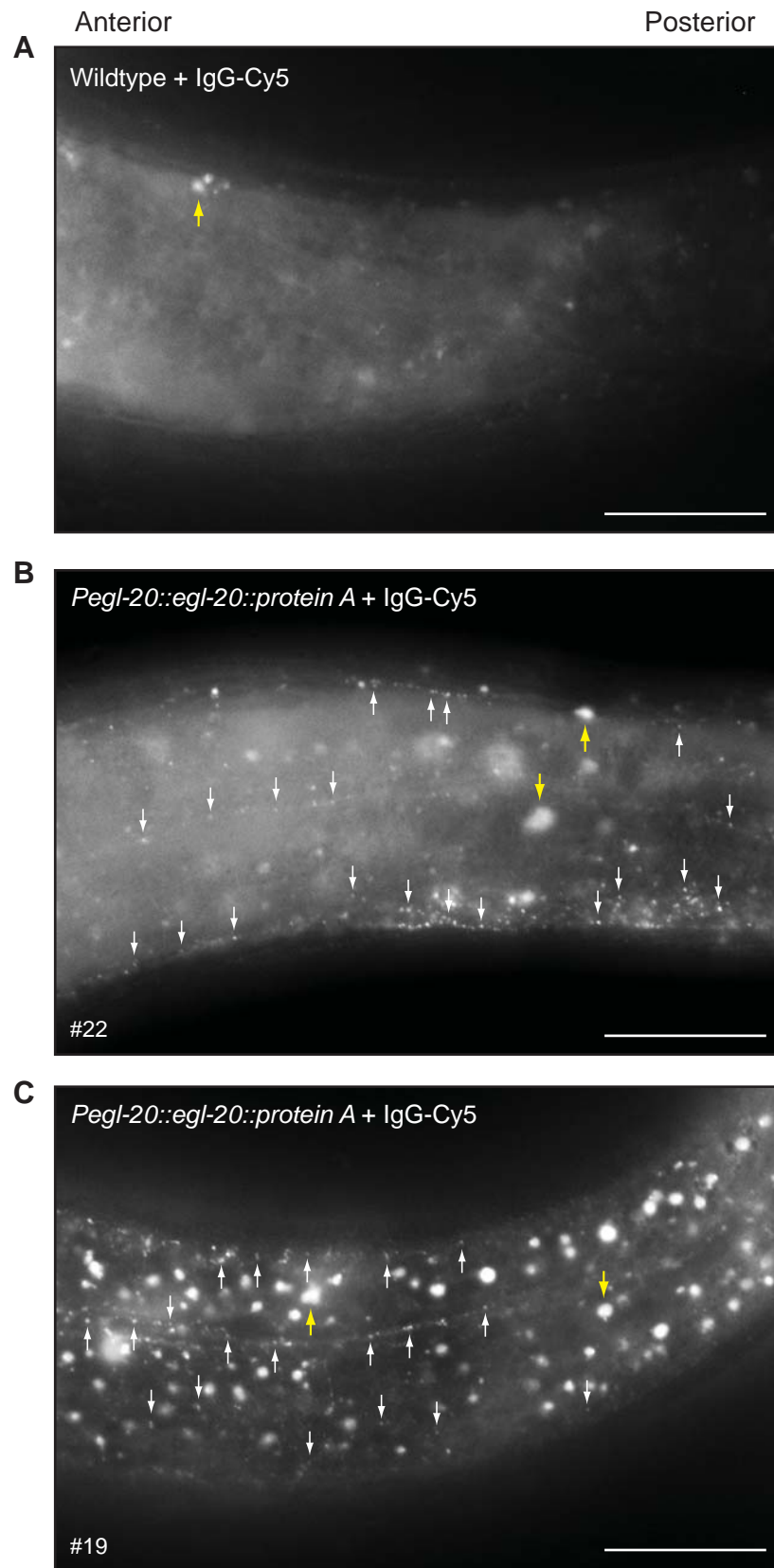

Supplement: Figure S12 — EGL-20/Wnt distribution in the posterior body of adult animals. EGL-20/Wnt was detected with the integrated Pegl-20::egl-20::protein A fusion transgene (huIs60) and injection of Cy5-conjugated rabbit IgG (IgG-Cy5) into living adult animals (B and C). White arrows point to examples of EGL-20/Wnt punctae, and yellow arrows indicate examples of variable background autofluorescence. Scale bars are 20 µm. Numbers in the panels denote particular injected animals. (PDF) [file pbio.1001465.s012.pdf]
