# Supplementary material for: Neurons Refine the Caenorhabditis elegans Body Plan by Directing Axial Patterning by Wnts
Source: PLoS Biol. 2013 Jan 8;11(1):e1001465. doi: 10.1371/journal.pbio.1001465 (PMC3539944; doi:10.1371/journal.pbio.1001465)
Supplement: Figure S6 — The reduced posterior body volume in vab-8 mutants is not sufficient to promote vulval fate signaling in epidermal progenitors. (A–D) Images showing posterior surface areas for posterior body volume calculations in wild-type, vab-8(lf), and dpy-17(lf); dpy-20(lf) mutants. Note that some vab-8 mutants with ectopic vulval fates at P8.p had posterior surface areas similar to those of some wild-type animals (compare [A] and [B]). (E) Table of posterior body volumes for wild-type, vab-8(lf), and dpy-17(lf); dpy-20(lf) mutants. Volumes were calculated by multiplying the mean thickness by the measured posterior surface area for each animal. p-Values were calculated using a two-tailed Student's t test. Note that the mean posterior body volumes of the total vab-8(lf) population, and of the vab-8 mutants specifically having ectopic vulval fates at P8.p, were not less than those of control dpy-17(lf); dpy-20(lf) mutants. (F and G) Body morphology of egfr/let-23(lf) mutant L1 larvae within minutes of being placed on 3% agar pads with either 50 mM (F) or 400 mM (G) sodium chloride. (H) Table of whole body volumes for egfr/let-23(lf) mutants as described in (E). The vab-8(lf) allele is gm138. Scale bar is 100 µm. (PDF) [file pbio.1001465.s006.pdf]

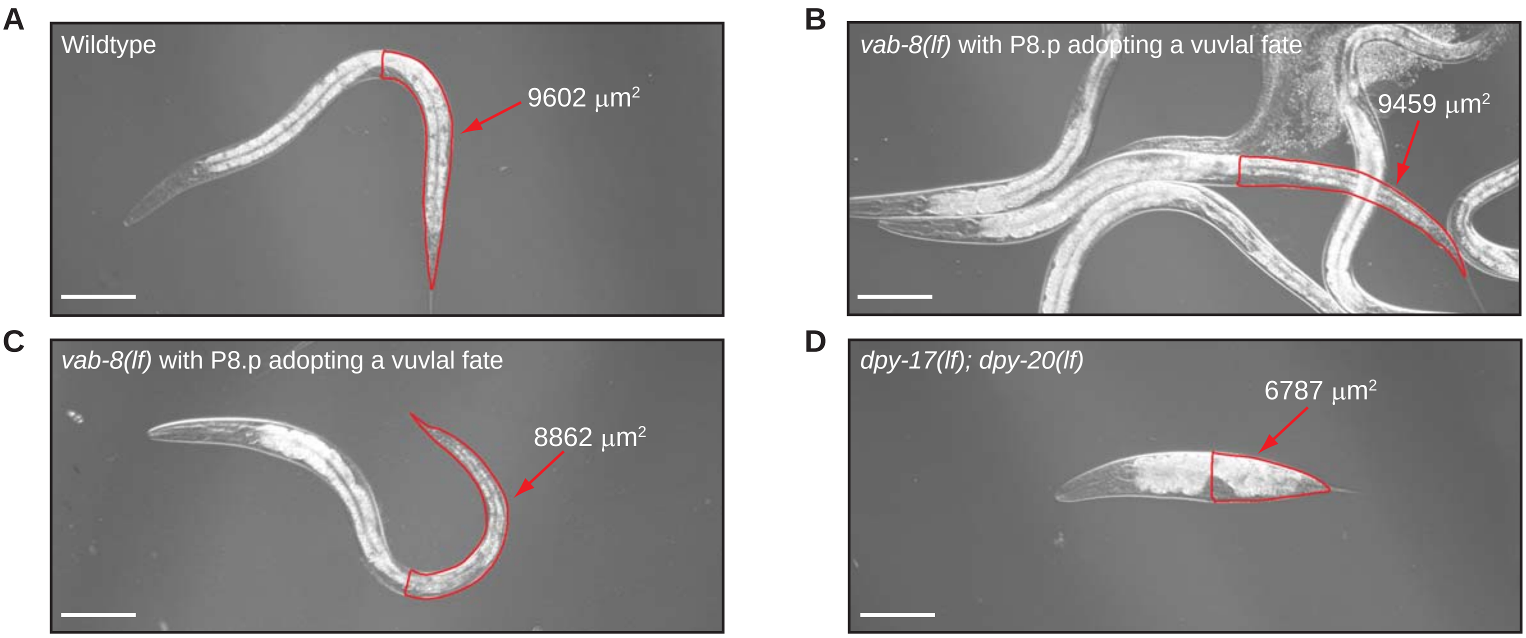

E

L4 Posterior body volumes

Genotype	Notes	Thickness (μm)	Volume (x 10^3 cubic microns)	*p*-Value (for volume)
Wildtype		33.8 ± 0.4 (n=5)	369 ± 31 (n=18)	
vab-8(lf)	Total population	30.7 ± 1.5 (n=6)	271 ± 35 (n=21)	<0.000001 vs Wildtype
vab-8(lf)	P8.p adopted a vulval fate	31.0 ± 1.0 (n=2)	258 ± 28 (n=3)	
dpy-17(lf); dpy-20(lf)		34.5 ± 2.3 (n=6)	245 ± 20 (n=17)	0.007 vs *vab-8(lf)*

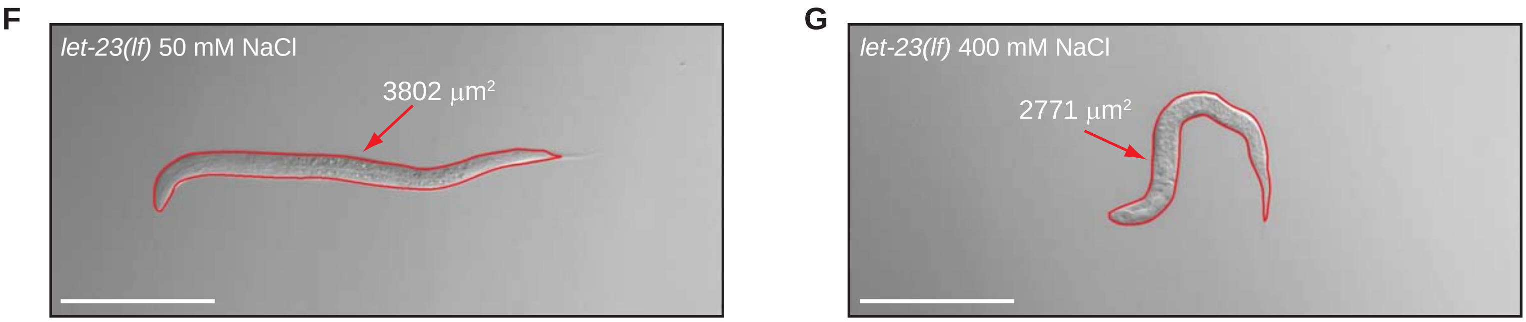

H

L1 Whole body volumes

Genotype	[NaCl]	Thickness (μm)	Volume (x 10^3 cubic microns)	*p*-Value (for volume)
let-23(lf)	50 mM	19.0 ± 0.8 (n=10)	76 ± 8 (n=19)	
let-23(lf)	400 mM	15.7 ± 1.8 (n=10)	48 ± 6 (n=22)	<0.000001 vs 50 mM NaCl
