# Supplementary material for: Neurons Refine the Caenorhabditis elegans Body Plan by Directing Axial Patterning by Wnts
Source: PLoS Biol. 2013 Jan 8;11(1):e1001465. doi: 10.1371/journal.pbio.1001465 (PMC3539944; doi:10.1371/journal.pbio.1001465)

A

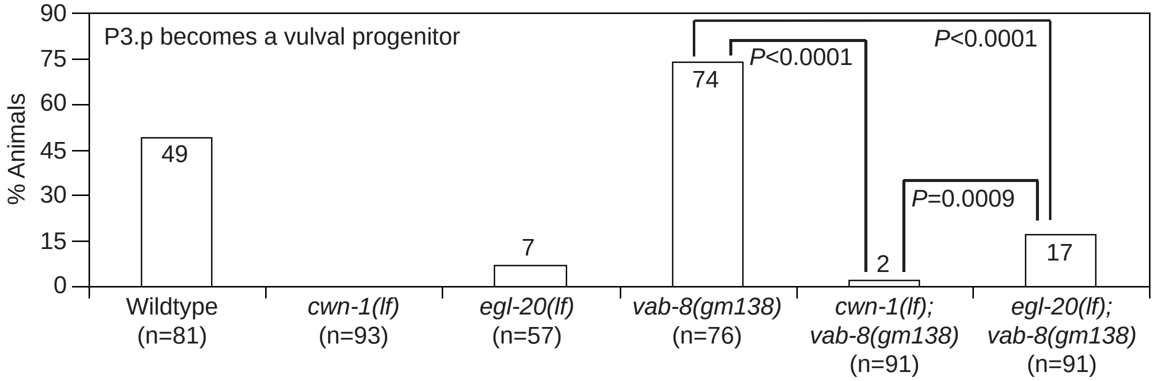

B

ceh-10 mutants where at least one of the cell bodies among the pair of CANs was close to P3.p

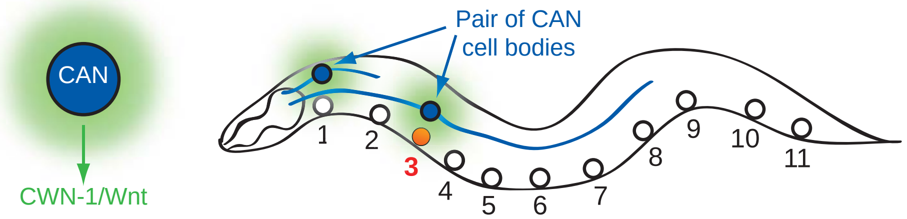

Animal	P3.p became a vulval progenitor	For the pair of CAN cell bodies: Closest body to P3.p	Furthest body from P3.p	For the pair of posterior CAN axons: Furthest terminus	Shortest terminus
92	No	P3.p	P3.p	P8.p/P9.p	P8.p/P9.p
72	No	P3.p	P2.p/P3.p	P8.p/P9.p	P8.p/P9.p
39	No	P3.p	P1.p/P2.p	P10.p/P11.p	P7.p progeny
58	No	P2.p/P3.p	P1.p/P2.p	P8.p/P9.p	P5.p progeny
82	No	P4.pa/P4.pp	P1.p/P2.p	P9.p/P10.p	P8.pa/P8.pp
38	No	P4.pp	P5.p progeny	P9.p/P10.p	P9.p/P10.p
44	Yes	P3.pa/P3.pp	Head/P1.p	P10.p/P11.p	P2.pa
90	Yes	P2.p/P3.pa	P2.p/P3.pa	P2.p/P3.pa	P2.p/P3.pa
76	Yes	P2.p/P3.pa	Head	P2.p/P3.pa	P1.p/P2.p
57	Yes	P2.p/P3.pa	Head	P2.p/P3.pa	P2.p/P3.pa
42	Yes	P2.p/P3.pa	Head	P7.p progeny	Head
68	Yes	P2.p/P3.pa	Head	P10.p/P11.p	P3.pa
67	Yes	P2.p/P3.pa	Head	P10.p/P11.p	P6.p progeny/P7.p progeny
48	Yes	P3.pp/P4.pa	P3.pp/P4.pa	P10.p/P11.p	P9.p/P10.p
59	Yes	P3.pp/P4.pa	Head	P8.pa/P8.pp	P1.p/P2.p
26	Yes	P4.pa/P4.pp	P1.p/P2.p	P9.p/P10.p	P2.p
55	Yes	P4.pa/P4.pp	Head	P2.p/P3.pa	Head

P3.p became a vulval progenitor	P3.p did not become a vulval progenitor
In 3/11 anmals, both posterior CAN axons reached P3.p (*P*=0.009)	In 6/6 anmals, both posterior CAN axons reached P3.p

C

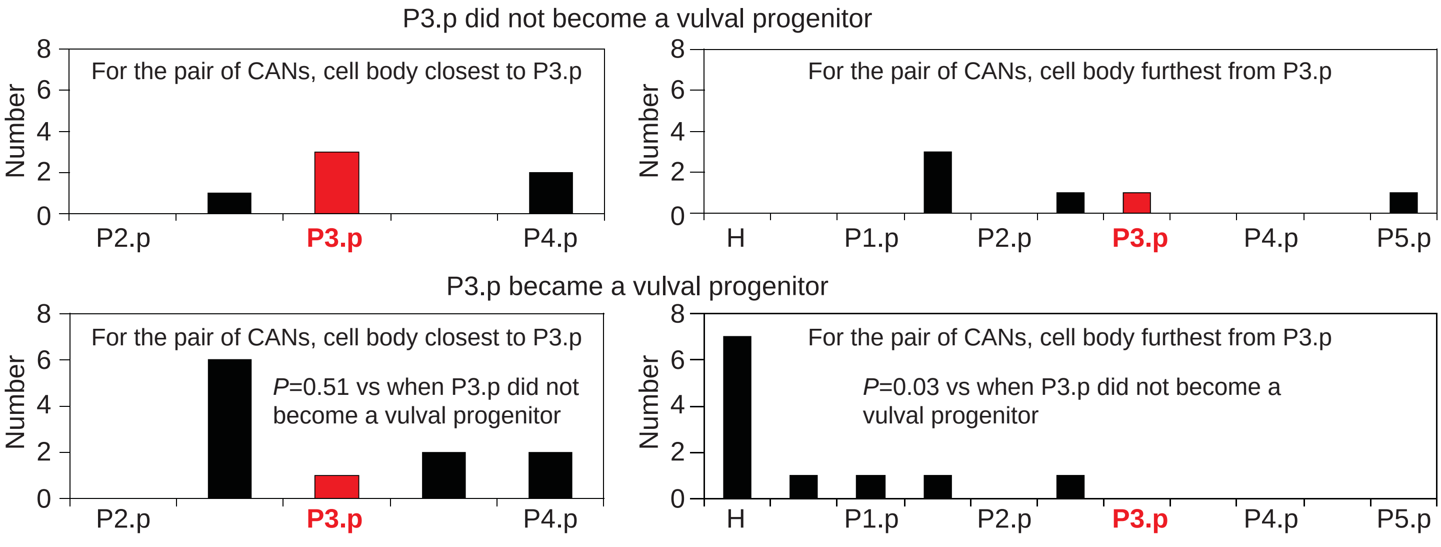

Supplement: Figure S9 — CAN cell body proximity to P3.p is not correlated with P3.p becoming a vulval progenitor. (A) cwn-1/wnt and egl-20/wnt activity are necessary for the increased frequency of P3.p becoming a vulval progenitor in vab-8 mutants. p-Values were calculated using a two-tailed Fisher's exact test. (B) Table of the positions of the two CAN cell bodies and their posterior axon termini in individual ceh-10 mutants, and whether P3.p became a vulval progenitor in these animals. The table includes only animals where at least one of the cell bodies among the pair of CANs was displaced closer to P3.p, away from the normal median position of P5.p. p-Value was calculated using a two-tailed Fisher's exact test. (C) Distributions of the CAN cell bodies closest and furthest to P3.p (among the pair) in animals where at least one cell body was close to P3.p, and relationship to P3.p becoming a vulval progenitor. H, head/pharyngeal region. p-Values were calculated using a two-tailed Mann-Whitney U test. (PDF) [file pbio.1001465.s009.pdf]
