# Supplementary material for: Neurons Refine the Caenorhabditis elegans Body Plan by Directing Axial Patterning by Wnts
Source: PLoS Biol. 2013 Jan 8;11(1):e1001465. doi: 10.1371/journal.pbio.1001465 (PMC3539944; doi:10.1371/journal.pbio.1001465)
Supplement: Figure S11 — Tail withering and reduced posterior body volumes do not account for the effects of ror/cam-1 mutations on Wnt signaling. (A–C) Images showing posterior surface areas for posterior body volume calculations in wild-type animals and ror/cam-1 mutants. Scale bar is 100 µm. Note that restoration of Ror/CAM-1 specifically to the CANs can restore inhibition of vulval development without rescuing tail withering (compare [B] and [C]). (D) Table of posterior body volumes for wild-type, ror/cam-1 mutant, and CAM-1-rescued animals. Volumes were calculated by multiplying the measured thickness and surface area for each animal. p-Values were calculated using a two-tailed Student's t test. Note that the ΔIntra ror/cam-1 construct that lacks the entire intracellular domain, but retains the Wnt-binding extracellular domain, does not rescue the withered tail phenotype of cam-1 mutants. Also note that ror/cam-1 mutants with increased signaling in vulval progenitors (mean 2.68 progenitors adopting vulval fates) do not have significantly greater tail withering than mutants with no increased signaling (mean 0.70 progenitors adopting vulval fates). (PDF) [file pbio.1001465.s011.pdf]

A B C

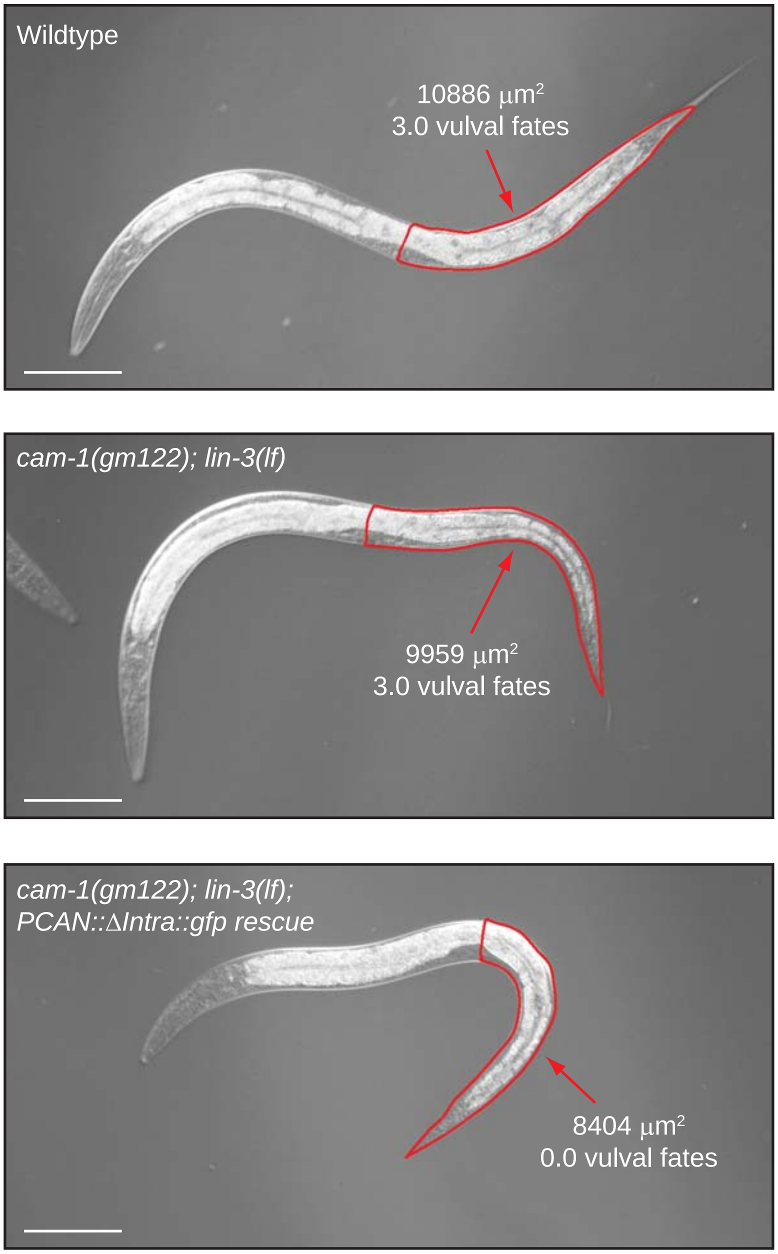

D

**Genotype**	**Notes**	**Thickness (μm)**	**Volume (x 10^3 cubic microns)**	***p*-Value (for volume)**
Wildtype		33.8 ± 0.4 (n=5)	369 ± 31 (n=18)	
cam-1(gm122); lin-3(lf)	Total population	33.5 ± 1.7 (n=21)	319 ± 46 (n=21)	0.0003 vs Wildtype<br>0.0005 vs *vab-8(gm138)*
cam-1(gm122); lin-3(lf);<br>*PCAN::ΔIntra::gfp rescue*	Total population	34.1 ± 1.0 (n=23)	327 ± 35 (n=23)	0.0002 vs Wildtype<br>0.54 vs *cam-1(gm122); lin-3(lf)*
cam-1(gm122); lin-3(lf)	Vulval fates: 0.70, n=10		324 ± 40 (n=10)	0.006 vs Wildtype
cam-1(gm122); lin-3(lf)	Vulval fates: 2.68, n=11		314 ± 52 (n=21)	0.008 vs Wildtype<br>0.62 vs siblings with 0.70 vulval fates
