# Supplementary material for: Neurons Refine the Caenorhabditis elegans Body Plan by Directing Axial Patterning by Wnts
Source: PLoS Biol. 2013 Jan 8;11(1):e1001465. doi: 10.1371/journal.pbio.1001465 (PMC3539944; doi:10.1371/journal.pbio.1001465)
Supplement: Table S1 — The CAN neurons inhibit vulval fate signaling. (DOC) [file pbio.1001465.s013.doc]

| **Genotype** | **Vulval fates^a^** | ***n*^b^** | ***p-*Value^c^** |
| --- | --- | --- | --- |
| Wildtype | 3.00 | 47 |  |
| *let-23(lf)* | 0.52 | 24 |  |
| *let-23(lf); vab-8(gm138)* | 1.95 | 20 | 0.0001 versus *let-23(lf)* |
|  |  |  |  |
| *let-23(lf); vab-8(gm138); dyEx12[Pvab-8s::vab-8s]* | 0.80 | 23 | 0.002 versus non transgenic |
| Control *dyEx12* non transgenic siblings^d^ | 2.03 | 20 |  |
| *let-23(lf); vab-8(gm138); dyEx11[Pvab-8s::vab-8s]* | 0.65 | 23 | 0.002 versus non transgenic |
| Control *dyEx11* non transgenic siblings^d^ | 1.78 | 23 |  |
|  |  |  |  |
| *let-23(lf); vab-8(gm138); dyEx22[PCAN::vab-8s]* | 0.65 | 20 | 0.0005 versus non transgenic |
| Control *dyEx22* non transgenic siblings^d^ | 1.90 | 24 |  |
|  |  |  |  |
| *let-23(lf); vab-8(gm138); dyEx23[Ppes-10::vab-8s]* | 1.77 | 22 | 0.46 versus non transgenic |
| Control *dyEx23* non transgenic siblings^d^ | 2.08 | 20 |  |
|  |  |  |  |

**Table S1.** **The CAN neurons inhibit vulval fate signaling**. ^a^Vulval fates: number of vulval progenitor cells adopting vulval fates. Wildtype is 3.00. ^b^*n*: number of animals assayed. ^c^*p-* Values were calculated using a two-tailed Student’s *t* test. ^d^Control animals were of the same genotype as the transgenic animals, but lacked the extrachromosomal transgenic array. *lf*, loss-of-function.
