# Supplementary material for: Neurons Refine the Caenorhabditis elegans Body Plan by Directing Axial Patterning by Wnts
Source: PLoS Biol. 2013 Jan 8;11(1):e1001465. doi: 10.1371/journal.pbio.1001465 (PMC3539944; doi:10.1371/journal.pbio.1001465)
Supplement: Table S2 — vab-8 mutants have a mild delay in larval growth that is not as pronounced as severe osmotic stress. (DOC) [file pbio.1001465.s014.doc]

|  |  | **Percentage of animals at or older than L4** | | | | | |
| --- | --- | --- | --- | --- | --- | --- | --- |
| **Genotype** | **[NaCl]** | **1 day** | **2 days** | **3 days** | **4 days** | **5 days** | ***n*^a^** |
|  |  |  |  |  |  |  |  |
| Wildtype | 50 mM | 0 | 100 |  |  |  | 60 |
| *vab8(gm99)* | 50 mM | 0 | 88 | 95 |  |  | 60 |
|  |  |  |  |  |  |  |  |
| *let-23(lf)* | 50 mM | 0 | 100 |  |  |  | 44 |
| *let-23(lf)* | 100 mM | 0 | 100 |  |  |  | 45 |
| *let-23(lf)* | 200 mM | 0 | 96 | 100 |  |  | 45 |
| *let-23(lf)* | 400 mM | 0 | 0 | 0 | 14 | 35 | 43 |
|  |  |  |  |  |  |  |  |

**Table S2**. ***vab-8* mutants have a mild delay in larval growth that is not as pronounced as severe osmotic stress.** L1 larvae were seeded under the indicated conditions and scored every 24 hours for the number of animals that reached or passed the L4 larval stage (end of vulval development). ^a^*n*: number of animals assayed. *lf*, loss-of-function.
