# Supplementary material for: Neurons Refine the Caenorhabditis elegans Body Plan by Directing Axial Patterning by Wnts
Source: PLoS Biol. 2013 Jan 8;11(1):e1001465. doi: 10.1371/journal.pbio.1001465 (PMC3539944; doi:10.1371/journal.pbio.1001465)
Supplement: Table S3 — Osmotic stress and a shortened body length do not promote vulval fate signaling. (DOC) [file pbio.1001465.s015.doc]

| **Genotype** | **Vulval fates^a^** | ***n*^b^** | ***p-*Value^c^** |
| --- | --- | --- | --- |
| Wildtype | 3.00 | 47 |  |
| *let-23(lf)* (50 mM NaCl-Normal salt) | 0.66 | 61 |  |
| *let-23(lf)*(100 mM NaCl) | 0.59 | 32 | 0.69 versus 50 mM NaCl |
| *let-23(lf)* (200 mM NaCl) | 0.66 | 64 | 0.96 versus 50 mM NaCl |
| *let-23(lf)* (400 mM NaCl) | 0.11 | 85 | 0.000005 versus 50 mM NaCl |
|  |  |  |  |
| *pmk-1(lf)* | 3.00 | 20 |  |
| *let-23(lf); vab-8(gm99)* | 1.85 | 24 | 0.0002 versus *let-23(lf)* |
| *let-23(lf); pmk-1(lf); vab-8(gm99)* | 2.25 | 20 | 0.34 versus *let-23(lf); vab-9(gm99)* |
| *let-23(lf); vab-8(gm138)* | 1.95 |  | 0.0001 versus *let-23(lf)* |
| *let-23(lf); pmk-1(lf); vab-8(gm138)* | 1.85 |  | 0.76 versus *let-23(lf); vab-8(gm138)* |
|  |  |  |  |
| *exc-5(lf)* | 3.00 | 21 |  |
| *let-23(lf); exc-5(lf)* | 0.70 | 23 | 0.49 versus *let-23(lf)* |
|  |  |  |  |
| *dpy-17(lf); dpy-20(lf)* | 3.08 | 90 |  |
| *let-23(lf); dpy-17(lf); dpy-20(lf)* | 0.46 | 23 | 0.76 versus *let-23(lf)* |
|  |  |  |  |

**Table S3.** **Osmotic stress and a shortened body length do not promote vulval fate signaling.** ^a^Vulval fates: number of vulval progenitor cells adopting vulval fates. Wildtype is 3.00. ^b^*n*: number of animals assayed. ^c^*p-*Values were calculated using a two-tailed Student’s *t* test. *lf*, loss-of-function.
