# Supplementary material for: Neurons Refine the Caenorhabditis elegans Body Plan by Directing Axial Patterning by Wnts
Source: PLoS Biol. 2013 Jan 8;11(1):e1001465. doi: 10.1371/journal.pbio.1001465 (PMC3539944; doi:10.1371/journal.pbio.1001465)
Supplement: Table S4 — Mutants with reduced vulval development that maintain wild-type Wnt pathway activity do not have increased sensitivity to Wnt gene activity. (DOC) [file pbio.1001465.s016.doc]

| **Genotype** | **Vulval Fates^a^** | ***n*^b^** | ***p-*Value^c^** |
| --- | --- | --- | --- |
| *sos-1(lf)* | 2.30 | 23 |  |
| *sur-6(lf)* | 3.00 | 20 |  |
| *sur-6(lf); sos-1(lf)* | 1.04 | 23 | 0.0003 versus *sos-1(lf)* |
|  |  |  |  |
| *egl-20(lf); sos-1(lf)* | 2.31 | 31 | 0.99 versus *sos-1(lf)* |
| *cwn-2(lf); sos-1(lf)* | 2.57 | 21 | 0.37 versus *sos-1(lf)* |
| *lin-44(lf); sos-1(lf)* | 2.63 | 20 | 0.22 versus *sos-1(lf)* |
| *sos-1(lf); vector RNAi* | 1.68 | 22 |  |
| *sos-1(lf); cwn-1 RNAi* | 1.59 | 22 | 0.79 versus *sos-1(lf); vector RNAi* |
| *sos-1(lf); mom-2 RNAi* | 1.73 | 22 | 0.90 versus *sos-1(lf); vector RNAi* |
|  |  |  |  |

**Table S4. Mutants with reduced vulval development that maintain wild-type Wnt pathway activity do not have increased sensitivity to Wnt gene activity.**   ^a^Vulval fates: number of vulval progenitor cells adopting vulval fates. Wildtype is 3.00. ^b^*n*: number of animals assayed. ^c^*p-*Values were calculated using a two-tailed Student’s *t* test. *lf*, loss-of-function.
