# Supplementary material for: Neurons Refine the Caenorhabditis elegans Body Plan by Directing Axial Patterning by Wnts
Source: PLoS Biol. 2013 Jan 8;11(1):e1001465. doi: 10.1371/journal.pbio.1001465 (PMC3539944; doi:10.1371/journal.pbio.1001465)
Supplement: Text S1 — Supplementary results, supplementary materials and methods, and supplementary references. (DOC) [file pbio.1001465.s017.doc]

**SUPPLEMENTARY RESULTS**

**Mispositioning of the CAN cell bodies and foreshortening of their posterior axons do not promote vulval fates by causing osmotic stress**

Since CAN axons travel close to the excretory cell canals that regulate osmotic balance , and because CAN laser ablation causes a lethal fluid-filled phenotype resembling ablation of components of the excretory system , we tested if abnormalities in CAN positioning and axon outgrowth might indirectly promote vulval fates by causing osmotic stress and/or disrupting excretory cell function. In these experiments, we focused on one representative property of *vab-8* mutations, which is the ability to increase vulval fate signaling in *egfr* pathway mutants, and hence, suppress vulvaless phenotypes. To directly test if osmotic stress is responsible for this suppression, we grew *egfr/let-23(lf)* L1 larval mutants in the presence of increasing concentrations of sodium chloride. Under normal salt conditions (50 mM NaCl), 100% of both wild-type and *egfr/let-23(lf)* mutant larvae reached or passed the L4 stage after two days (Table S2). In contrast, *vab-8* mutants showed a mild delay in growth with 88% of L1 larvae reaching the L4 stage after two days, and 95% of larvae reaching the L4 stage by three days (Table S2). Mild to moderate osmotic stresses of 100-200 mM sodium chloride did not affect larval growth rate (Table S2). However, 400 mM sodium chloride delayed larval growth much more severely than mutation of *vab-8*. After three days, when 95% of *vab-8* mutants had reached or developed past the L4 stage, none of the larvae growing in the presence of 400 mM sodium chloride reached this stage (Table S2). In fact, even after five days, only 35% of larvae growing in the presence of 400 mM sodium chloride reached the L4 stage. Based on these data, if mutation of *vab-8* causes osmotic stress, it must be less severe than that caused by 400 mM sodium chloride. However, unlike *vab-8* mutations, none of the tested salt stresses from 100-400 mM sodium chloride promoted vulval development in *egfr/let-23* mutants (Table S3). In fact, the osmotic stress associated with 400 mM sodium chloride actually had the opposite effect of *vab-8* mutations, and exacerbated the vulvaless phenotype of *egfr/let-23* mutants (Table S3).

To further rule out that *vab-8* mutations promote vulval fate signaling by affecting the excretory system, we also examined excretory cell morphology in *vab-8* mutants. In wild-type animals, excretory cell canals traveled the normal distance from the head to the posterior rectal region (Figure S1A). While 100% of control *exc-5* mutants had readily detectable canal defects including truncated canals and cysts (Figure S1B), *vab-8(gm138)* mutants resembled wild-type animals and exhibited normal excretory canals (Figure S1C). To directly test whether disruption of excretory cell function promotes vulval fates, we tested if the same *exc-5* allele that causes excretory cell defects in 100% of animals, phenocopies *vab-8* mutations and suppresses the underinduced phenotype of an *egfr/let-23(lf)* mutation. In contrast to *vab-8* mutations, the *exc-5* mutation did not suppress the underinduced phenotype of the *egfr/let-23(lf)* mutation (Table S3). Finally, the main pathway known to be activated by osmotic stress in *C. elegans* is a stress response p38 MAPK pathway, which is essential for viability during osmotic stress . If *vab-8* mutations cause osmotic stress, the p38 MAP kinase pathway might be expected to contribute to the promotion of vulval fates. However, a loss-of-function mutation in *p38mapk/pmk-1* did not block the ability of *vab-8* mutations to suppress the underinduced phenotype of an *egfr/let-23(lf)* mutation (Table S3). Collectively, these data strongly argue against the hypothesis that most of the effects of *vab-8* mutations on epidermal development are due to osmotic stress, abnormal excretory cell function, or osmotic stress-associated signaling.

**The stimulation of vulval fates observed in *vab-8(lf)* mutants is specifically sensitive to reductions in Wnt gene activity**

Primary vulval fates are normally induced by a single strong EGF signal and the redundant activities of multiple Wnts. Due to the strength of the EGF signal, no single Wnt gene is absolutely essential for vulval development (Table 2). However, we speculated that in *egfr/let-23(lf); vab-8(lf)* double mutants that have reduced EGF pathway signaling and increased Wnt signaling due to displacement of the CANs, the intermediate level of vulval development in this background would be very sensitive to the activities of individual Wnt genes. Consistent with this model, mutation or RNAi of three individual Wnt genes did not affect vulval development in an otherwise normal background, but completely suppressed vulval development in *egfr/let-23(lf); vab-8(lf)* double mutants (Table 2).

To examine the importance of the genetic background (and signaling pathway activity) for sensitivity to single gene mutations in the Wnt family, we examined *sos-1(ku231)* mutants. *sos-1(ku231)* mutants have a weak reduction in the activity of the EGF pathway Ras exchange factor, SOS-1 , and have a level of vulval development that is comparable to *egfr/let-23(lf); vab-8(lf)* double mutants (Table S4). In these mutants, the intermediate level of vulval development arises from a slight reduction in EGF pathway activity, and presumably, does not reflect changes in the normal levels of Wnt signaling. Therefore, unlike *egfr/let-23(lf); vab-8(lf)* double mutants, the *sos-1(ku231)* mutants should not be any more dependent on Wnt signaling than wild-type animals. Consistent with this model, the *sos-1(ku231)* underinduced phenotype is exacerbated by mutations that further compromise EGF pathway activity such as mutation of *pp2a-b/sur-6*, but not by individual mutations in any of the Wnt genes (Table S4). Thus, sensitivity to single gene mutations in the Wnt family may identify mutant backgrounds that are dependent on elevated Wnt signaling.

**Tail withering is not the major mechanism by which foreshortening of the posterior CAN axon promotes vulval fates**

Strong *vab-8* mutations cause a gradual post-embryonic withering of the posterior half of the animal, which is correlated with anterior displacement of the CANs . This withering is also correlated with foreshortening of the posterior CAN axon, and can be quantified as a reduction in the distance of epidermal progenitors to the anus (Figure S5A). As the median position of the posterior axon terminus shifts more anterior from wild-type animals to *vab-8(ev411)*, *ceh-10(lf)*, and *vab-8(gm138)* mutants, there is a progressive shortening of the distance of epidermal progenitors such as P8.pp to the anus. This withering could indirectly promote Wnt signaling by increasing the effective concentration of posterior-derived Wnts such as EGL-20, in the vicinity of posteriorly-shifted progenitors. To address this possibility, we first examined whether there was any correlation between the proximity of a particular Pn.px cell to the anus and activation of the *syIs187* (POPTOP) reporter. In wild-type animals and *vab-8(gm99)* mutants showing reporter activity in P6.px cells, P6.pp was not any closer to the anus than in animals without reporter activity (Figure S5B). While for P8.pp cells there was a tendency for the reporter to be more active in P8.pp cells that were closer to the anus, this tendency was not statistically significant (Figure S5B). Since the increased proximity of the vulval progenitors to the anus is also a quantitative measure of the degree of whole body withering, these data also indicate that withering, in general, is not the main reason for increased Wnt signaling in the vulval progenitors of *vab-8* mutants.

We also combined *dpy-17* and *dpy-20* mutations that affect the cuticle to create a background in which CAN axon displacement was not affected, but Pn.px cells were positioned even closer to the anus than in strong *vab-8* mutants. In twelve *dpy-17(lf); dpy-20(lf)* mutants, 23/24 posterior CAN axons terminated past P11.p (Figure S5C, D), which is similar to the distribution in wild-type animals (Fig. 5B). However, in *dpy-17(lf); dpy-20(lf)* mutants, the mean distance of P8.pp to the tail was only 46 μm, as compared to 94 μm in *vab-8(gm138)* mutants (Figure S5A). Despite the greatly increased proximity of Pn.px cells to posterior-derived EGL-20/Wnt, the *dpy-17(lf); dpy-20(lf)* background did not result in the same epidermal phenotypes as observed in *vab-8(gm99)* and *vab-8(gm138)* mutants. Simultaneous mutation of *dpy-17* and *dpy-20* did not cause a P-Rvl phenotype (0%, n=90), an increase in the incidence of P3.p becoming a vulval progenitor, ectopic vulval fates, or suppress the underinduced phenotype of *egfr/let-23(lf)* mutants (Figure S5E, S5F, Table S3).

We also considered the possibility that the reduced posterior body volume associated with tail withering might increase the concentrations of posteriorly-enriched Wnts such as EGL-20 and CWN-1, thereby increasing the strength of their signaling. To quantify the reduction in posterior body volume, we measured the posterior surface area in L4 stage animals at the end of vulval development, but when the withering phenotype was more pronounced. We then measured posterior worm thickness, and by multiplying these values, obtained an approximation of posterior body volume. Using this method, we determined that on average, *vab-8* mutants have 73% of wild-type posterior body volume (Figure S6). We then calculated the posterior body volume in our control *dpy-17(lf); dpy-20(lf)* double mutants and found that they only had 66% of normal posterior body volume. The *dpy-17(lf); dpy-20(lf)* mutant volume is significantly less than in *vab-8* mutants (Figure S6). Thus, despite having both less posterior body volume and increased proximity of epidermal progenitors to Wnt sources than in *vab-8* mutants, *dpy-17(lf); dpy-20(lf)* double mutants do not display Wnt gain-of-function phenotypes. We also determined that an osmotic stress of 400 mM sodium chloride, which did not promote vulval fate signaling, reduced total body volume even more than *vab-8* mutations to only 63% of wild-type animals (Figure S6F-H). These data rule out that physical changes affecting progenitor distance to Wnt sources or body volume are the main reasons for why *vab-8* mutants show Wnt gain-of-function phenotypes.

During the course of our studies with mutations causing tail withering, we also were able to identify animals that allowed us to separate withering from functional increases in Wnt signaling. Even in specific *vab-8* mutants with ectopic vulval fates in the posterior P8.p progenitor (and hence, elevated Wnt signaling), their posterior body volumes were still in the range of or greater than in *dpy-17(lf); dpy-20(lf)* control mutants that never have ectopic vulvae at P8.p (Figure S6E). Moreover, in at least one *vab-8* mutant with ectopic vulval fates at P8.p, the posterior body volume was actually similar to that in some wild-type animals (Figure S6A, B). We also examined individual *ceh-10* mutants for correlations between the position of posterior CAN axon termination, presence of ectopic vulvae at P8.p, and the distance of either uninduced P8.px cells or ectopic vulvae to the anus (Figure S8). In general, in *ceh-10* mutants with ectopic vulvae at P8.p, the longest posterior CAN axon was shorter than in wild-type animals, and the induced P8.p progeny were closer to the anus than the uninduced P8.p progeny in wild-type animals (115 μm vs. 135 μm, respectively). However, in three individual *ceh-10* mutants with ectopic vulvae at P8.p, the distances of the induced progeny to the anus were actually greater than the mean distance of uninduced P8.px cells to the anus in siblings. In animals 79, 74, and 45, the ectopic vulvae at P8.p were 138 μm, 145 μm, and 153 μm, respectively, from the anus, which were all greater than the mean distance of 135 μm between uninduced P8.px cells and the anus in siblings. In contrast to the absence of a strict correlation between distance of the P8.px cells to the anus and the ectopic induction of vulval fates, all three of these *ceh-10* mutants (animals 79, 74, 45) had severely foreshortened posterior CAN axons, with the final positions terminating in the head/pharyngeal region or close to the P3.px cells. In yet a third genetic background, we found that while in *ror/cam-1* mutants the tail withering phenotype is quite mild (86% of normal posterior body volume, Figure S11), the epidermal phenotypes are equally strong as in *vab-8* mutants that have more severe tail withering. Furthermore, while using suppression of the vulvaless phenotype of an *egf/lin-3(lf)* mutation as a readout for the increased Wnt signaling in *ror/cam-1* mutants, we noticed that posterior body volumes were the same regardless of whether suppression had occurred (Figure S11D). Animals with an average vulval induction of 0.70 (not suppressed) had the same posterior body volume as animals with an average induction of 2.68 (suppressed). As further evidence for morphological tail withering not being the root cause of Wnt gain-of-function phenotypes in these mutants, we found that a transgenic array expressing an intracellular domain-deleted form of Ror/CAM-1 can fully restore inhibition to vulval fate signaling, without rescuing the withered tail phenotype (Figures S11, 7E).

Finally, some of the strongest evidence arguing against CAN displacement causing Wnt gain-of-function phenotypes simply by altering tail morphology include the observations that Wnt gain-of-function phenotypes are also observed in embryos, prior to the manifestation of any tail withering phenotype. Laser ablation of the CAN precursors promotes embryonic HSN overmigration , a phenotype that is well-associated with elevated activity of the posterior-derived EGL-20/Wnt . In addition, we found that VAB-8 also acts through the CANs to minimize EGL-20/Wnt-dependent P11 to P12 fate transformations (Figure S4G). Collectively, these data provide multiple lines of evidence to indicate that post-embryonic morphological changes in the posterior body, are not the major reasons for why CAN-displacing mutations increase Wnt signaling in epidermal progenitors.

**SUPPLELEMENTARY MATERIALS AND METHODS**

**Strains**

*C. elegans* were cultured at 20ºC . Loss-of-function alleles include LGI: *lin-44(n1792)* , *sur-6(ku123)* ; LGII: *cwn-1(ok546)*, *let-23(sy1)* , *unc-4(e120)* , *cam-1(gm122)* , *cam-1(ks52)* , *cam-1(sa692)* ; LGIII: *ceh-10(gm120)* , *dpy-17(e164)* , *pha-1(e2123ts)* ; LGIV: *lin-3(n378)* , *let-60(sy95dn)* , *unc-24(e138)* , *egl-20(n585)* , *cwn-2(ok895)*, *dpy-20(e1282)* , *exc-5(rh232)* , *pmk-1(km25)* , *sfrp-1(gk554)*; LGV: *vab-8(gm99)* , *vab-8(gm138)* , *vab-8(ev411)* , *him-5(e1490)* , *sos-1(ku231)* . Integrated transgenes include *muIs49* , *syIs187* and *syIs188* , *zdIs13* , *mgIs42* , *huIs60* , and *cwIs6* [contains a full length rescuing genomic *Pcam-1::cam-1::gfp* construct; a gift from Wayne Forrester ], *kyIs4* , and *bgIs312* . The deletion alleles *cwn-1(ok546)* and *cwn-2(ok895)* were generated by *C. elegans* Gene Knockout Facility at the Oklahoma Medical Research Foundation, and the s*frp-1(gk554)* deletion was generated by *C. elegans* Reverse Genetics Core Facility at the University of British Columbia.

**Plasmids**

***vab-8* constructs.** The *vab-8s* cDNA was amplified by PCR from wild-type cDNA using a nested PCR strategy of vab8-2 5'-GCAAGCCGTTGAGTGTTGAGACAAAG-3' and vab8-3 5'-TTTCTGCAGAAGCACGTCGCCCATT G-3', followed by vab8-1 5'-ACATGCATGCAAAA ATGAGCTCTCCAACACACAACTGCCAC-3' and vab8-3. The resulting product adds a Kozak sequence and an in-frame ATG upstream of the first nucleotide of exon 9 and extends just past the PstI site in exon 13. This product, which adds 17 amino acids to the smallest product previously shown to rescue the CAN migration defect in *vab-8* mutants (D isoform) , was digested with PstI and cloned in-frame into the PstI site of the *gfp* expression vector *pPD95.81* to yield *pPD95.81vab-8s*.

The putative *vab-8s* promoter is located in the eighth intron, just upstream of exon 9. Primers vab8-7 5'-GACATGCATGCATACGTATG CAACAATGTTTGCAC-3' and vab8-8 5'-GACATGCATGCTCTGCAAGGCGCTTTTATTGG-3' were used to amplify from *dpy-20(lf); him-5(lf)* genomic DNA a 1542 bp fragment in this region that spans nucleotides 9364-10906 of cosmid C35G11. This fragment includes 1540 bp of sequence 5’ to the first nucleotide of exon 9 and has a putative TATA box at position 756 bp. The promoter fragment was blunt-end cloned into the EcoRV site of *pBS* (Stratagene) to yield *pBSvab-8s*. To make the *Pvab-8s::DsRed2* reporter construct, we first generated *pPD95.75DsRed2* by replacing the GFP gene in *pPD95.75* with the *DsRed2* gene from *pDsRed2-C1* (Clontech) using AgeI/EcoRI digestion. The *vab-8s* promoter was then digested with SphI and cloned into the SphI site of the *DsRed2* expression vector. The *vab-8s* promoter-driven *vab-8s* cDNA expression vector, *Pvab-8s::vab-8s::gfp* was constructed by transferring the *vab-8s* promoter from *pBSvab-8s* into *pPD95.81vab-8s* via Sph I digestion.

To make *Ppes-10::vab-8s::gfp*, we first amplified the *pes-10* promoter from *pPAP-10* (a gift from Susan Mango) with the primers pap10-1 5'-GACATGCATGCGGATCCAATCAATGCCTGAAAG-3' and pap10-2 5'-GACATGCATGCGGCCTGCAGGATCGATTTTTTGC-3'. This fragment contains a minimal region of the *pes-10* promoter which spans -203 to -23 relative to the start ATG. The amplified fragment was digested with SphI and cloned into SphI-digested *pUC19*. To generate *Ppes-10::vab-8s::gfp*, the *pes-10* promoter was excised from *pUC19pes-10* via SphI digestion and cloned into the SphI site of *pPD95.81vab-8s*.

***egl-20*, *cwn-1*, and CAN promoters.** The *egl-20* promoter was amplified by PCR from *Pegl-20::TAP* using the primers egl20-3 5'-TACATGCATGCTCGATGAATACGGTAGAACCAAG-3' and egl20-4 5'-GACATGCATGCTATTTCTGAAATTGAGATGTTTTAG-3'. The resulting product spans 12480-16900 of W08D2 and includes 4421 bp of sequence 5’ to the *egl-20* start ATG. The product was digested with SphI and cloned into the SphI site of *pUC19*. After cloning into *pUC19*, the promoter was excised with SphI digestion and then cloned into the SphI site of *pPD95.75DsRed2* to yield *Pegl-20::DsRed2*.

The *cwn-1* promoter was amplified from the cosmid K10B4 with the primers cwn1-3 5'-TGCACATGCATGCCGACCGCACCCCGAATTGAAGC-3' and cwn1-10 5'-CTAGTCTAGATTCATAATAATTTTCAGACTGAAAG-3'. The product contains 1941 bp of sequence 5’ to the *cwn-1* start ATG and was cloned via SphI/XbaI digestion into *pSX95.75yfp* (a gift from Shawn Xu) to yield *Pcwn-1::yfp* and into *pPD95.75DsRed2* to yield *Pcwn-1::DsRed2*.

A CAN-specific enhancer element located between -1332 and -791 relative to the *ceh-23* start ATG was amplified from *Pceh-23promL* (a gift from Oliver Hobert) using primers ceh23-3 5'-TCTGCATGCATACCATAATTGTTCAAAATATGATAG-3' and ceh23-4 5'-GCAGAACCAATGCATCAAAGCTCACAGGCTAATGTGC-3'. This fragment was digested with NsiI and cloned into the PstI site of *pPAP-10*. The fusion of the *ceh-23* CAN-specific enhancer to the minimal *pes-10* promoter was amplified from this plasmid using ceh23-2 5'-GGACATGCATGCACCTACATAATTGTTCAAAATATGATAG-3' and pap10-1 5'-GACATGCATGCGGATCCAATCAATGCCTGAAAG-3', digested with SphI, and cloned into the SphI site of *pUC19* to generate *pUCceh-23pes-10*. The *ceh-23::pes-10* promoter was then excised from the *pUC19* plasmid via SphI digestion and cloned into the SphI sites of *pPD95.81vab-8s* and *pSX95.75YFP*, yielding *PCAN::vab-8s::gfp* and *pCAN::yfp*.

***cam-1* constructs**. To construct *PCAN::cam-1::gfp*, *cam-1::gfp* was excised from *Phs::cam-1::gfp* with XbaI and ApaI digestion and cloned into the same sites in *PCAN::yfp*. To construct *pCAN::ΔIntra::gfp*, a *cam-1* fragment encoding the transmembrane domain and part of the extracellular domain was amplified from *pMini3* with the primers cam1-6 5’-GTG GAC GGT TAC CAG ACG AAG AAG-3’ and cam1-7 5’-CAGTA GG TAC CTG ATG ACG TGT CTT TTG AGA CTT C-3’. This fragment was then cloned into *pPD95.79* via SalI/KpnI digestion to generate an in-frame translational fusion with GFP. The *cam-1::gfp* fusion was then excised from *pPD95.79* via XhoI digestion and used to replace the corresponding XhoI fragment in *pCAN::cam-1::gfp*. The final construct encodes the entire CAM-1 extracellular domain and extends through the first twelve amino acids just after the end of the transmembrane domain. Due to the cloning strategy, five additional amino acids are inserted between the end of CAM-1 and the beginning of the GFP sequence.

***Pceh-23::DsRed2.*** We attempted to build a DsRed2 version of *Pceh-23::gfp* , which is strongly expressed in the CAN neurons, as well as several additional neurons in the head and tail. The *DsRed2* plasmid was constructed from *Pceh-23::snb-1::mCherry*, which was generated as follows. The *snb-1* coding region without the STOP codon was first amplified from *Punc-129::snb-1* (a gift from Peter Juo) with the primers snb1-1 5'-GATCCCCCGGGAAAAATGGACGCTCAAGGAGATGCC-3' and snb1-2 5'-GGATACCGGTTTTTCCTCCAGCCCATAAAACGATGATG-3', which added 5’ SmaI and 3’ AgeI restriction sites, and cloned into *pBS* yielding *pBSsnb-1*. The *snb-1* coding region from *pBSsnb-1* was then cloned downstream of the *ceh-23* promoter in *Pceh-23::gfp* via SmaI digestion to generate *Pceh-23::snb-1*. The *mCherry* coding region was amplified from *pmCherry* (a gift from Erik Jorgensen) with the primers mCherry-1 5'-CCATAACCGGTGTGAGCAAGG GCGAGGAGG-3’ and mCherry-2 5'-GCATTACCGGTTACTTGTACAGCTCGTCCATGC CGC-3', which introduce AgeI restriction sites into the product, and cloned into *pBS* to generate *pBSmCherry*. *mCherry* was then removed from *pBSmCherry* with AgeI digestion and cloned into the AgeI site downstream of *snb-1* in *Pceh-23::snb-1*, yielding *Pceh-23::snb-1::mCherry.* In transgenic animals, this construct recapitulated the expression pattern normally observed with *Pceh-23::gfp*. *Pceh-23::snb-1::mCherry* was then digested with SphI and SmaI, and the resulting ~7.0 kbp fragment cloned into the SphI and SmaI sites of *pPD95.75DsRed2* (see *vab-8* constructs). We generated one transgenic array with *Pceh-23::DsRed2*, *dyEx44*. However, none of these animals showed detectable *DsRed2* expression in the CAN neurons or any head or tail neurons. Thus, either due to an error in cloning, or the absence of the key *ceh-23* promoter elements in this 7.0 kbp fragment, this construct is not functional.

**Transgenics**

Transgenic extrachromosomal arrays were generated as described . Transgenic extrachromosomal arrays were generated by injecting the following mixtures of plasmids: *dyEx11*, *dyEx12* [*pBX-1*(100 ng/μl); *Pvab-8s::vab-8::gfp* (100 ng/μl); *Pmyo-3::DsRed2* (20 ng/μl)*]*; *dyEx20*, *dyEx22* [*PCAN::vab-8s::gfp* (100 ng/μl), *pBX-1* (100 ng/μl), *Pmyo-3::DsRed2* (20 ng/μl)]**;** *dyEx23*, *dyEx24* [*Ppes-10::vab-8s::gfp* (100 ng/μl), *pBX-1* (100 ng/μl), *Pmyo-3::DsRed2* (20 ng/μl)]; *dyEx34* [*Pcwn-1::yfp* (50 ng/μl), *Pmyo-3::DsRed2* (20 ng/μl), *pBX-1* (100 ng/μl)]; *dyEx44* [*PCAN::cam-1::gfp* (75 ng/μl), *Pegl-20::DsRed2* (30 ng/μl), *pBX-1* (100 ng/μl), *Pceh-23::DsRed2** (50 ng/μl)] [*Note that the *Pceh-23::DsRed2* plasmid (see Plasmid section) did not drive detectable *DsRed2* expression.]; *dyEx45* [*PCAN::ΔIntra::gfp* (75 ng/ml), *myo-2::mcherry* (a gift from Villu Maricq) (2.5 ng/ml), *pBX-1* (100 ng/ml)]; *akEx906* [*Pegl-20::DsRed2* (70 ng/μl); *PCAN::YFP* (70 ng/μl); *Pmyo-2::cfp* (10 ng/μl); *pBX-1* (100 ng/μl)];*akEx908*[*Pcwn-1::DsRed2* (75 ng/μl), *PCAN::yfp* (75 ng/μl), *pBX-1* (100 ng/μl)]; *akEx923*[*PCAN::yfp* (70 ng/μl), *Pvab-8s::DsRed2* (70 ng/μl), *pBX-1* (100 ng/μl)]**;** *akEx1601*[*pCAN::cam-1::gfp* (50 ng/μl), *pmyo-3::mCherry* (10 ng/μl), *pBX-1* (100 ng/μl), *pBS* (40 ng/μl)]**;** *syEx1024* .

**RNA interference**

HT115 *E. coli* bacteria containing RNAi clones (L4440, empty RNAi vector; K100B4.6, *cwn-1*; F38E1.7, *mom-2*) were obtained from the Ahringer bacterial feeding library , and RNAi was performed as described .

**RNA preparation and Real-Time PCR**

Gravid worms growing on 12-14 60 mm dishes were bleached for three minutes in a solution containing 0.7N sodium hydroxide and 29% commercial bleach, washed twice in M9 buffer, and the resultant eggs plated on NG plates without bacteria. After 24 hours, synchronized L1 worms were washed from the plates, lysed in RNA lysis buffer (RNAqueous –micro kit, Ambion) and snap frozen in liquid nitrogen. Once all samples had been collected, RNA was prepared according to the manufacturer’s instructions, including a DNAse treatment to remove contaminating genomic DNA. RNA yield and integrity were analyzed on the Bio-Rad Experion automated electrophoresis system using the standard sensitivity detection reagents. Samples having a 28S rRNA:18S rRNA ratio of 1.4-2.0 were used for further analysis. 100-700 ng of total RNA were used for cDNA synthesis using a High Capacity cDNA Reverse Transcription Kit (Ambion), following the manufacturer’s instructions. Real-time PCR was conducted with the equivalent of 4 ng of input RNA in 10 ul reactions containing Taqman FAST Universal Master Mix (ABI) and 0.5 ul Taqman probe (ABI) [*tbp-1*, Ce02448934_m1

; *egl-20*, Ce02458265_g1]. Cycling was performed in a Bio-Rad CFX96 Real-Time System using an initial denaturation of 95^o^C for 20 seconds, followed by 40 cycles of 95^o^C 3 seconds; 60^o^C for 30 seconds. All reactions were performed in triplicate. Fold differences were calculated by the ΔΔCt method (ΔCt=Ct_target_-Ct_tbp-1_; ΔΔCt=ΔCt_mutant_-ΔCt_wildtype_; Fold difference=2^ΔΔCt^).

**Osmotic stress**

NG plates were prepared with either standard (50 mM) or higher concentrations (100 mM, 200 mM, 400 mM) of sodium chloride. After preparation, OP50 *E. coli* were seeded onto plates and were stored for up to one week at 20^o^C before use. Gravid worms were bleached on standard NG plates without bacteria in drops of commercial bleach/4N sodium hydroxide (1:1). The next day, synchronized L1 worms were seeded onto three replicate plates of each salt concentration. Larvae were scored for vulva development at the mid-L4 stage, and were not scored beyond six days post-seeding. Osmotic stress experiments were performed twice and data were pooled from both experiments.

**Excretory cell scoring**

Excretory cell morphology was visualized using the integrated *Ppes-6::gfp* reporter *bgIs312*. L4 larvae were anesthetized in 10 mM sodium azide on 3% agar pads. Worms were examined using epifluorescence on an Olympus IX81 inverted microscope equipped with a DSU confocal spinning disk, a Rolera MGI Fast camera, 60X and 100X objective lenses, and Invivo software (MediaCybernetics). Both the lengths of the excretory cell canals and the presence or absence of cysts were scored.

**Measurement of posterior body volumes**

Body volumes were approximated by multiplying the surface area of the posterior half of the animal (from the midpoint of the vulva or the position of P6.p progeny in vulvaless animals to close to the tail tip) with the thickness of the worm. Surface area measurements were made using the outline spline tool in Axiovision (Zeiss). Worm thickness was measured midway from the center of the animal to the tail (approximately the end of the posterior gonad) on a Zeiss Axioimager Z1 microscope by using Axiovision to determine the stage position at the two extreme vertical focal planes.

**HSN position scoring**

HSN neurons were visualized using the integrated *Ptph-1::gfp* reporter *mgIs42*. Young adults were anesthetized with 5 mM levamisole. Worms were photographed as z-stacks in epifluorescence and DIC channels using an Axioimager Z1 microscope and Axiovision software (Zeiss). After stacks were captured, the positions of the two HSN cell bodies were determined relative to Pn.p nuclei and the vulval slit.

**Pn.px distance measurements**

Distances between Pn.p progeny and the EGL-20/Wnt-producing rectal cells were estimated by measuring the distance between the Pn.px cell and the anus using the curve spline measurement tool in Axiovision (Zeiss).

**SUPPLEMENTARY REFERENCES**

1. Nelson F, Riddle D (1984) Functional study of the C. elegans secretory-excretory system using laser microsurgery. Journal of Experimental Zoology 231: 45-56.

2. Forrester W, Garriga G (1997) Genes necessary for C. elegans cell and growth cone migrations. Development 124: 1831-1843.

3. Solomon A, Bandhakavi S, Jabbar S, Shah R, Beitel GJ, et al. (2004) Caenorhabditis elegans OSR-1 regulates behavioral and physiological responses to hyperosmotic environments. Genetics 167: 161-170.

4. Chang C, Hopper N, Sternberg P (2000) Caenorhabditis elegans SOS-1 is necessary for multiple Ras-mediated developmental signals. EMBO Journal 19: 3283-3294.

5. Manser J, Wood W (1990) Mutations affecting embryonic cell migrations in Caenorhabditis elegans. Developmental Genetics 11: 49-64.

6. Forrester WC, Kim C, Garriga G (2004) The Caenorhabditis elegans Ror RTK CAM-1 inhibits EGL-20/Wnt signaling in cell migration. Genetics 168: 1951-1962.

7. Pan CL, Howell JE, Clark SG, Hilliard M, Cordes S, et al. (2006) Multiple Wnts and frizzled receptors regulate anteriorly directed cell and growth cone migrations in Caenorhabditis elegans. Dev Cell 10: 367-377.

8. Zinovyeva AY, Yamamoto Y, Sawa H, Forrester WC (2008) Complex network of Wnt signaling regulates neuronal migrations during Caenorhabditis elegans development. Genetics 179: 1357-1371.

9. Jiang L, Sternberg P (1998) Interactions of EGF, Wnt and Hom-C genes specify the P12 neuroectoblast fate in C. elegans. Development 125: 2337-2347.

10. Brenner S (1974) The genetics of Caenorhabditis elegans. Genetics 77: 71-94.

11. Herman M, Vassilieva L, Horvitz H, Shaw J, Herman R (1995) The C. elegans gene lin-44, which controls the polarity of certain asymmetric cell divisions, encodes a Wnt protein and acts cell nonautonomously. Cell 83: 101-110.

12. Sieburth D, Sundaram M, Howard R, Han M (1999) A PP2A regulatory subunit positively regulates Ras-mediated signaling during Caenorhabditis elegans vulval induction. Genes & Development 13: 2562-2569.

13. Aroian R, Sternberg P (1991) Multiple functions of let-23, a Caenorhabditis elegans receptor tyrosine kinase gene required for vulval induction. Genetics 128: 251-267.

14. White J, Southgate E, Thomson J (1992) Mutations in the Caenorhabditis elegans unc-4 gene alter the synaptic input to ventral cord motor neurons. Nature 355: 838-841.

15. Forrester W, Perens E, Zallen J, Garriga G (1998) Identification of Caenorhabditis elegans genes required for neuronal differentiation and migration. Genetics 148: 151-165.

16. Koga M, Take-uchi M, Tameishi T, Ohshima Y (1999) Control of DAF-7 TGF-B expression and neuronal process development by a receptor tyrosine kinase KIN-8 in Caenorhabditis elegans. Development 126: 5387-5398.

17. Kim C, Forrester WC (2003) Functional analysis of the domains of the C elegans Ror receptor tyrosine kinase CAM-1. Dev Biol 264: 376-390.

18. Granato M, Schnabel H, Schnabel R (1994) pha-1, a selectable marker for gene-transfer in C. elegans. Nucleic Acids Research 22: 1762-1763.

19. Ferguson E, Horvitz H (1985) Identification and characterization of 22 genes that affect the vulval cell lineages of the nematode C. elegans. Genetics 110: 17-72.

20. Han M, Aroian R, Sternberg P (1990) The let-60 locus controls the switch between vulval and nonvulval cell fates in Caenorhabditis elegans. Genetics 126: 899-913.

21. Han M, Sternberg P (1991) Analysis of dominant negative mutations of the Caenorhabditis elegans let-60 ras gene. Genes & Development 5: 2188-2198.

22. Riddle D (1978) The genetics of development and behavior in C. elegans. Journal of Nematology 10: 1-16.

23. Trent C, Tsung N, Horvitz H (1983) Egg-laying defective mutants of the nematode C. elegans. Genetics 104: 619-647.

24. Hosono R, Hirahara K, Kuno S, Kurihara T (1982) Mutants of C. elegans with Dumpy and Rounded head phenotype. Journal of Experimental Zoology 224: 135-144.

25. Buechner M, Hall D, Bhatt H, Hedgecock E (1999) Cystic canal mutants in Caenorhabditis elegans are defective in the apical membrane domain of the renal (excretory) cell. Developmental Biology 214: 227-241.

26. Mizuno T, Hisamoto N, Terada T, Kondo T, Adachi M, et al. (2004) The Caenorhabditis elegans MAPK phosphatase VHP-1 mediates a novel JNK-like signaling pathway in stress response. Embo J 23: 2226-2234.

27. Wightman B, Clark S, Taskar A, Forrester W, Maricq A, et al. (1996) The C. elegans gene vab-8 guides posteriorly directed axon outgrowth and cell migration. Development 122: 671-682.

28. Hodgkin J, Horvitz H, Brenner S (1979) Nondisjunction mutants of the nematode C. elegans. Genetics 91: 67-94.

29. Whangbo J, Kenyon C (1999) A Wnt signaling system that specifies two patterns of cell migration in C. elegans. Molecular Cell 4: 851-858.

30. Green JL, Inoue T, Sternberg PW (2008) Opposing Wnt pathways orient cell polarity during organogenesis. Cell 134: 646-656.

31. Clark SG, Chiu C (2003) C. elegans ZAG-1, a Zn-finger-homeodomain protein, regulates axonal development and neuronal differentiation. Development 130: 3781-3794.

32. Sze JY, Victor M, Loer C, Shi Y, Ruvkun G (2000) Food and metabolic signalling defects in a Caenorhabditis elegans serotonin-synthesis mutant. Nature 403: 560-564.

33. Coudreuse DY, Roel G, Betist MC, Destree O, Korswagen HC (2006) Wnt gradient formation requires retromer function in Wnt-producing cells. Science 312: 921-924.

34. Forrester W, Dell M, Perens E, Garriga G (1999) A C. elegans Ror receptor tyrosine kinase regulates cell motility and asymmetric cell division. Nature 400: 881-885.

35. Zallen J, Kirch S, Bargmann C (1999) Genes required for axon pathfinding and extension in the C. elegans nerve ring. Development 126: 3679-3692.

36. Berry KL, Bulow HE, Hall DH, Hobert O (2003) A C. elegans CLIC-like protein required for intracellular tube formation and maintenance. Science 302: 2134-2137.

37. Wolf F, Hung M, Wightman B, Way J, Garriga G (1998) vab-8 is a key regulator of posteriorly directed migrations in C. elegans and encodes a novel protein with kinesin motor similarity. Neuron 20: 655-666.

38. Wenick AS, Hobert O (2004) Genomic cis-regulatory architecture and trans-acting regulators of a single interneuron-specific gene battery in C. elegans. Dev Cell 6: 757-770.

39. Mello CC, Kramer JM, Stinchcomb D, Ambros V (1991) Efficient gene transfer in C.elegans: extrachromosomal maintenance and integration of transforming sequences. Embo J 10: 3959-3970.

40. Kamath RS, Fraser AG, Dong Y, Poulin G, Durbin R, et al. (2003) Systematic functional analysis of the Caenorhabditis elegans genome using RNAi. Nature 421: 231-237.

41. Modzelewska K, Elgort MG, Huang J, Jongeward G, Lauritzen A, et al. (2007) An activating mutation in sos-1 identifies its Dbl domain as a critical inhibitor of the epidermal growth factor receptor pathway during Caenorhabditis elegans vulval development. Mol Cell Biol 27: 3695-3707.
